# Revison of *Metaplagia* Coquillett (Diptera: Tachinidae) with description of five new species from Area de Conservación Guanacaste in northwestern Costa Rica

**DOI:** 10.3897/BDJ.9.e68598

**Published:** 2021-07-29

**Authors:** AJ Fleming, D. Monty Wood, M. Alex Smith, Winnie Hallwachs, Daniel Janzen

**Affiliations:** 1 Agriculture Agri-Food Canada, Ottawa, Canada Agriculture Agri-Food Canada Ottawa Canada; 2 University of Guelph, Guelph, Canada University of Guelph Guelph Canada; 3 Department of Biology, University of Pennsylvania, Philadelphia, United States of America Department of Biology, University of Pennsylvania Philadelphia United States of America

**Keywords:** tropical rain forest, tropical dry forest, cloud forest, parasitoid flies, host-specificity, caterpillars, ACG, Dexiinae, Voriini

## Abstract

**Background:**

We revise the genus *Metaplagia* Coquillett, 1895 and describe five new species from Area de Conservación Guanacaste (ACG) in northwestern Costa Rica. All new species were reared from an ongoing inventory of wild-caught caterpillars spanning a variety of species within the family Sphingidae (Lepidoptera: Sphingidae). Our study provides a concise description of each new species using morphology, life history, molecular data and photographic documentation. In addition to the new species, the authors provide a re-description of the genus and a revised key to the species of *Metaplagia*.

**New information:**

The following five new species of *Metaplagia* are described: *Metaplagia leahdennisae* Fleming & Wood **sp. n.**, *Metaplagia lindarobinsonae* Fleming & Wood **sp. n.**, *Metaplagia paulinesaribasae* Fleming & Wood **sp. n.**, *Metaplagia robinsherwoodae* Fleming & Wood **sp. n.** and *Metaplagia svetlanakozikae* Fleming & Wood **sp. n.**

The following is proposed by Fleming & Wood as new combination of *Plagiomima* Brauer & Bergenstamm, 1891: *Plagiomima latifrons* (Reinhard, 1956) **comb. n.**

## Introduction

The tribe Voriini is distributed globally; the vast tachinid fauna of the Neotropical Region and the huge number of genera have proven to be one of the most significant hurdles to understanding the tribal boundaries surrounding the Voriini. Most attempts to understand the synapomorphies or of the tribe have, rather than answered questions, led researchers to even more questions as to what the voriines truly are. Presently, the tribe possesses several diagnostic features which the authors consider as typical to the tribe: conical head profile (longer at level of pedicel than at vibrissa); proclinate, divergent and well-developed ocellar setae; frons wide; proclinate and reclinate orbital setae present in both sexes; facial ridge bare; prosternum bare; anepimeral seta absent or poorly developed appearing hair-like; infrasquamal setae present; apical scutellar setae strong and decussate; dm-m crossvein oblique, making posterior section of M_4_ subequal to anterior section; R_4+5_ setulose at least to crossvein r-m and sometimes beyond; middorsal depression of ST1+2 reaching posterior margin; and aedeagus elongate and frequently ribbon-like (Tschorsnig 1985, Cortés and González 1989). Voriini lay flattened membranous incubated eggs directly on the cuticle of the host (Herting 1956, Guimarães 1977) . Unfortunately, some of these diagnostic features can be observed across several other taxa within the Dexiinae and, as such, our recognition of the tribe can be seen as a combination of these features used to exclude other genera and define the Voriini; much variation exists within the catch-all tribe and the "gestalt" of the tribe is still somewhat nebulous. The Voriini are a problematic tribe and still the subject of much debate. Recent work has suggested that the Voriini are a vast unifying polyphyletic clade encompassing much of the Dexiinae (Stireman et al. 2019), only further emphasizing the catch-all nature of the tribe; this evidence, however, is still the subject of discussion.

The monotypic genus *Metaplagia* Coquillett, 1895 (Dexiinae: Voriini) was initially erected for the type species *Metaplagia occidentalis* Coquillett, 1895 (Coquillett 1895). Coquillet based the new species on a single male specimen he collected in San Diego Co. California. The genus was previously known exclusively from the Nearctic; however, the data we present herein suggests the genus reaches into the Neotropics. A significant obstacle to revising a genus like *Metaplagia* is that the interspecific morphological differences are often subtle and, in many cases, variable. However, despite their morphological similarities, when examined at a genetic level, they display a great deal of interspecific variation, allowing the individual species to be further discriminated.

Our goal is to systematically revise and analyze the known members of the New World tachinid genus *Metaplagia* Coquillett and add to the existing taxa five new species from Area de Conservación Guanacaste (ACG). Our species concepts are based on differences in morphology, life history and by comparison of COI (coxI or cytochrome *c* oxidase I) gene sequences. We also provide a key to the species of *Metaplagia* inclusive of the Nearctic and Mesoamerican Regions. This paper is part of a broader effort to name and catalog all of the tachinid species collected from the ACG inventory, an effort which has already presented new information within the tribe Voriini (Fleming et al. 2017, Fleming et al. 2019). This series of taxonomic papers will represent a foundation for later, detailed ecological and behavioral accounts and studies, extending across ACG ecological groups, whole ecosystems and taxonomic assemblages much larger than a genus. All new information described here derives from the ongoing inventory of the tri-trophic relationships between caterpillars, their food plants and their parasitoids within the dry, rain and cloud forests of the terrestrial portion of ACG (Janzen et al. 2009, Janzen et al. 2011, Janzen and Hallwachs 2011, Janzen et al. 2020). This inventory started in 1978 and has yielded an unprecedented amount of invaluable ecological information on the tri-trophic relationships between parasitoids, hosts and associated food plants.

## Materials and methods

### Project aims and rearing intensity

All reared specimens were obtained from host caterpillars collected in ACG (Janzen and Hallwachs 2016). ACG's 125,000+ terrestrial hectares span the Provinces of Alajuela and Guanacaste, along the dry forested northwestern coast of Costa Rica and inland to the Caribbean lowland rain forest. ACG comprises several different biomes and intergrades, ranging from sea level up to 2,000 m. The tachinid rearing methods are described at http://janzen.bio.upenn.edu/caterpillars/methodology/how/parasitoid_husbandry.htm. Since its inception, this inventory has reared over 750,000 wild-caught ACG caterpillars. Any frequencies of parasitization reported here need to be considered against this background inventory. Comparative details of the parasitization ecology of these flies will be treated separately in later papers, in the context of the study of all parasitization rates of tachinids on ACG caterpillars, once the overall alpha taxonomy of ACG caterpillar-attacking tachinids is more complete than at present.

### Voucher specimen management

The management of voucher specimens has been detailed in previous papers in this series (Fleming et al. 2014). In brief, all caterpillars reared from the ACG efforts receive a unique voucher code in the format yy–SRNP–xxxxx. Any parasitoid emerging from a caterpillar receives the same voucher code as a record of the rearing event. If and when the parasitoid is later dealt with individually, it receives a second voucher code unique to it, in the format DHJPARxxxxxxx. These voucher codes, assigned to both host and parasitoids, may be used to obtain the individual rearing record at http://janzen.bio.upenn.edu/caterpillars/database.lasso.

To date, all DHJPARxxxxxx-coded tachinids have had one leg removed for DNA barcoding at the Biodiversity Institute of Ontario (BIO) in Guelph, ON, Canada. All successful barcodes and collateral data are first deposited in the Barcode of Life Data System (BOLD, www.boldsystems.org) (Ratnasingham and Hebert 2007) and later migrated to GenBank. Each barcoded specimen is also assigned unique accession codes from both the Barcode of Life Data System (BOLD) and GenBank, respectively.

Inventoried Tachinidae were collected under Costa Rican government research permits issued to DHJ and exported from Costa Rica to Philadelphia, en route to their final depository in the Canadian National Insect collection in Ottawa, Canada (CNC). Tachinid identifications for the inventory were done by DHJ in coordination with: a) visual inspection by AJF and DMW, b) DNA barcode sequence examination by MAS and DHJ and c) correlation with host caterpillar identifications by DHJ and WH through the inventory itself. Dates of collection, cited for each ACG specimen, are the dates of eclosion of the fly, not the date of capture of the caterpillar since the fly eclosion date is much more representative of the time when that fly species is on the wing than is the time of capture of the host caterpillar. The collector listed on the label is the parataxonomist who found the caterpillar, rather than the person who retrieved the newly-eclosed fly from its rearing container. The holotypes and paratypes of the species newly-described herein are all deposited at CNC.

### Descriptions and imaging

Species accounts and descriptions are deliberately brief and concise, complemented by a series of color photos of every species, used to illustrate the morphological differences amongst them. The morphological terminology used follows Cumming and Wood (2017). All dissections and photography were carried out following the methods detailed in Fleming et al. (2014). If only one male were available, it was designated as the holotype and not subjected to dissection.

### Interim names of undescribed host species

Names of undescribed host species follow a standardized, interim naming system used for taxonomic units considered as distinct species and identified by DNA barcodes. The interim names are given in the format "*Manduca sexta*DHJ02" or "*Manduca sexta*DHJ03", where the "species epithet" is either composed of the name of the taxonomist who identified the species and a number or the name of a species-group, followed by a code. This prevents confusion with already-described species, while maintaining traceability of each undescribed species within the ACG project.

### DNA barcoding

We generated DNA extracts from single legs using a glass fibre protocol (Ivanova et al. 2006), using the standard DNA barcode region (5’ cytochrome c oxidase I (COI) gene) for all specimens of ACG *Metaplagia*. The DNA barcodes (658 bp near the 5’ terminus of the COI gene) were amplified using general insect primers, using standard protocols for both production and quality control (Smith et al. 2006, Smith et al. 2007, Smith et al. 2008, Smith et al. 2009, Smith 2012). DNA sequences, trace files and accessions were deposited in the Barcode of Life Data System (BOLD) (Ratnasingham and Hebert 2007). Metadata (including GenBank accession codes) associated with each sequence can be consulted on BOLD by using the persistent DOI (https://doi.org/10.5883/DS-ASMETAPL).

**Figure 1.**
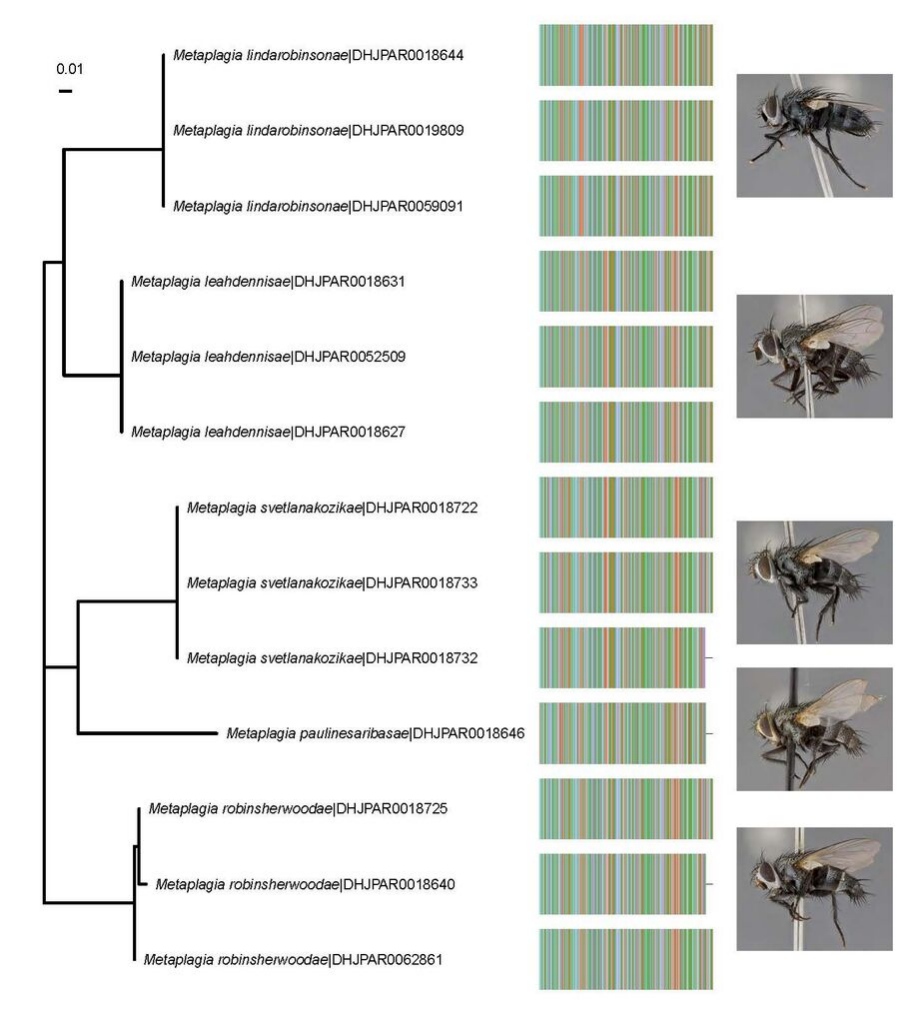
A phylogeny of the type series of *Metaplagia* species described from Area de Conservacion Guanacaste. Tip photos are the holotypes of the species.

The phylogeny (Fig. 1) was created using the Maximum Likelihood method, based on the Tamura 3-parameter model (Tamura 1992) created using holo- and paratype DNA barcode sequences. Aligned with each tip of the tree (species name|DHJPARxxxxxxx accession) is a pictorial representation of the DNA barcode of that specimen and the lateral image of the holotype for that species. The best nucleotide substitution model and the phylogeny itself were created using MEGA X (Kumar et al. 2018) and the figure created using the R (R Core Team 2019) package ggtree (Yu et al. 2017).

### Acronyms for depositories

CNC, Canadian National Collection of Insects, Arachnids and Nematodes, Ottawa, Canada

UCDC, University of California, Davis, Bohart Museum of Entomology, Davis, California, USA

USNM, United States National Museum of Natural History, Washington, D.C., USA

## Taxon treatments

### Metaplagia

Coquillett, 1895

99DD501A-FD14-559E-8E94-C11410424201

Metaplagia Coquillett, 1895: 102 [also 1895: 97]. Type species: *Metaplagia occidentalis* Coquillett, 1895, by original designation.
Metavoria Townsend, 1915: 101. Type species: *Metavoria orientalis* Townsend, 1915, by original designation. Synonymy proposed by Wood 1987: 1250, and further clarified in O'Hara and Wood 1998: 755.
Agathomyia Reinhard, 1959: 228 (preocc. by Verrall, 1901). Type species: *Agathomyia cordata* Reinhard, 1959, by original designation. Synonymy proposed by Wood 1987: 1250, and further clarified in O'Hara and Wood 1998: 763.
Anzamyia Reinhard, 1960: 103. (nomen novum for *Agathomyia*, 1959 Reinhard).
Metaplagia brevicornis Brooks, 1945: 81. Holotype male (CNC), by original designation. Type locality: Canada, Manitoba, Teulon.
Agathomyia cordata Reinhard, 1959: 229. Holotype female (UCDC) (1 female paratype in CNC), by original designation. Type locality: USA, California, Riverside County, Anza.
Metavoria facialis Reinhard, 1956: 123. Holotype female (CNC). Type locality: USA, Utah, Fruitland.
Metaplagia occidentalis Coquillett, 1895: 103. Holotype male (USNM, Type No. USNMENT01519725), by original designation. Type locality: USA, California, San Diego County.
Metavoria orientalis Townsend, 1915: 101. Holotype female (USNM, Type No. USNMENT01519726), by original designation. Type locality: USA, Virginia, Arlington.
Metaplagia Metaplagia occidentalis Coquillett, 1895

#### Description

Male, **head**: frontal vitta wide, 1/3-1/6th width of front-orbital plate; with 2–3 proclinate orbital setae and 1–2 reclinate orbital setae; ocellar setae proclinate slightly divergent; eye bare, not descending beyond the level of the vibrissa; fronto-orbital plate coloration ranging from shining silver to gold; fronto-orbital plate with short black setulae interspersed amongst frontal setae; fronto-orbital setae not extending below lower margin of pedicel, with fronto-orbital setulae sometimes extending below lower margin of pedicel; pedicel orange, with a black-dark brown post pedicel; arista bare and subequal to slightly shorter than postpedicel, distinctly-thickened on basal 1/2-2/3, ranging from dark orange to dark brown-black; lower margin of face almost level with vibrissa, not visible in profile; facial ridge bare, but with setulae along parafacial sometimes so close to facial ridge as to be confused with facial ridge setulae; palps either straight or with a slight club at apex, sparsely setulose. **Thorax**: tomentosity ranging from pale beige-gray to dark grey or silver over a black ground color; thorax black ground color tomentum of thorax ranging from pale brassy to silver grey; prosternum bare; 3–4 postpronotal setae arranged in a straight line; supra-alar setae 1–2:3; intra-alar setae 2–3:3; dorsocentral setae 3:3–4; acrostichal setae 3:3; katepisternum with 3 setae. Scutellum black ground color, with tomentum ranging from gray to pale brassy; with five pairs marginal setae; apical scutellar setae 1/2–1/3 as long as subapical scutellars, sub-erect, slightly above marginal plane; basal scutellar subequal in length to subapical setae; subapical setae straight, ranging from divergent to convergent. Legs: dark reddish-brown to black ground color; tarsal claws and pulvilli ranging from shorter than to longer than last tarsomere. Wings: slightly longer than abdomen; translucent slightly hyaline; R_1_ and R_4+5_ can be setulose, setulae of R_4+5_ ranging from node to crossvein r-m or beyond. **Abdomen**: ground color black, pale silver tomentum in varying degrees on T3–T5, ST1+2 typically glabrous; middorsal depression on ST1+2 reaching to hind margin of tergite; median marginal setae present on T3 and complete rows on T4 and T5; median discal setae absent on all tergites; sex patch absent.

**Terminalia**: posterior margin of sternite 5 with a deeply excavated and wide U-shaped (sometimes sculptured) median cleft; lateral lobes of sternite apically rounded, often with setae along caudal margin; basal section 1/5 the length of apical lobes. Epandrium often with 3–4 long, strong setae along anal edge. Cerci, in posterior view, medially separated, but parallel and often touching so as to appear fused, with a few short setae on basal half. In lateral view, bowed and sharply tapered apically. Surstylus well-developed, stout basally in lateral view, like a stout broadly rounded triangle terminating in a small knob, appearing hooked or slightly beaked apically; in posterior view, basally enlarged and apically straight.

Female as in male, except in the following aspects: **head**: tomentum of fronto-orbital plate and parafacial can sometimes differ from that of the conspecific male; fronto-orbital plate and parafacial up to 0.5x wider than in males.

#### Diagnosis

*Metaplagia* can be distinguished by the following combination of traits: head distinctly conical; males with upper frontal setae reclinate; proclinate orbital setae in both sexes; frontal setae descending below level of pedicel; both sexes with well-developed lateral vertical setae; eye bare; parafacial setulose, but not with strong stout setae, only hair-like setulae; genal dilation very slightly developed; prementum shorter than height of head with an enlarged labellum; prosternum bare; three postsutural supra-alar setae, the anteriormost reduced and much weaker than first postsutural dorsocentral seta; scutellum with four pairs of marginal setae and one pair of erect to semi-erect apical setae; one or two pairs of sub-erect discal setae on scutellum, in line with subapical setae; vein M_1_ ending separately in wing margin; anepimeral seta short, not extending beyond edge of lower calypter; wing vein R_4+5_ setulose.

#### Distribution

From Manitoba, Canada east to Newfoundland and south to Costa Rica.

#### Ecology

Within the ACG inventory, *Metaplagia* has only been reared from the Lepidoptera family Sphingidae: Lepidoptera throughout the diverse ecosystems of the research area.

#### Taxon discussion

Our present revision of *Metaplagia* increases the range of the genus, taking it from being a Nearctic endemic genus to a complete New World distribution, inclusive of both the Nearctic and Neotropical Regions.

Both *Plagiomima* Brauer & Bergenstamm, 1891 and *Metaplagia* are extremely similar genera; however, these can be very easily distinguished by the conformation of their mouthparts (Wood and Zumbado 2010). After careful examination of the holotype of *Metaplagia latifrons* Reinhard and based on the generic concepts and diagnostic features of both *Metaplagia* and *Plagiomima*, the authors hereby propose that species *Metaplagia latifrons* Reinhard, 1956 be transferred into the genus *Plagiomima*, as *Plagiomima latifrons* (Reinhard) **comb. n.** We base this taxonomic action on the similarities presented between the slender elongated mouthparts and the slender apically pointed surstyli present in *Metaplagia latifrons*, both diagnostic features observed in the genus *Plagiomima*, yet absent from the genus *Metaplagia*.

### Metaplagia leahdennisae

Fleming & Wood sp. n.

BF3CCA89-B8E6-5548-BE62-6BC7D4C75B0D

A4C3E7CC-DCCF-4EA3-B963-FA2BF3A99017

#### Materials

- **Type status:** Holotype. **Occurrence:** catalogNumber: DHJPAR0018627; recordedBy: D.H. Janzen, W. Hallwachs & Manuel Pereira; individualCount: 1; sex: Male; lifeStage: adult; occurrenceStatus: Present; preparations: Pinned; otherCatalogNumbers: ASTAI1274-07| 98-SRNP-8248| BOLD:AAE2876; **Taxon:** scientificName: Metaplagia leahdennisae; phylum: Arthropoda; class: Insecta; order: Diptera; family: Tachinidae; genus: Metaplagia; specificEpithet: leahdennisae; scientificNameAuthorship: Fleming & Wood, 2021; **Location:** continent: Central America; country: Costa Rica; countryCode: CR; stateProvince: Guanacaste; county: Sector Santa Rosa; locality: Area de Conservacion Guanacaste, Quebrada Costa Rica; verbatimElevation: 275; verbatimLatitude: 10.8274; verbatimLongitude: -85.6365; verbatimCoordinateSystem: Decimal degrees; **Identification:** identifiedBy: AJ Fleming; dateIdentified: 2021; **Event:** samplingProtocol: Reared from the larva of the Sphingidae, Agrius cingulata; verbatimEventDate: 10/Jul/98; **Record Level:** language: en; institutionCode: CNC; collectionCode: Insects; basisOfRecord: PreservedSpecimen
- **Type status:** Paratype. **Occurrence:** catalogNumber: DHJPAR0018631; recordedBy: D.H. Janzen, W. Hallwachs & Guillermo Pereira; individualCount: 1; sex: Male; lifeStage: adult; occurrenceStatus: Present; preparations: Pinned; otherCatalogNumbers: ASTAI1278-07| 98-SRNP-8346| BOLD:AAE2876; **Taxon:** scientificName: Metaplagia leahdennisae; phylum: Arthropoda; class: Insecta; order: Diptera; family: Tachinidae; genus: Metaplagia; specificEpithet: leahdennisae; scientificNameAuthorship: Fleming & Wood, 2021; **Location:** continent: Central America; country: Costa Rica; countryCode: CR; stateProvince: Guanacaste; county: Sector Santa Rosa; locality: Area de Conservacion Guanacaste, Quebrada Costa Rica; verbatimElevation: 275; verbatimLatitude: 10.8274; verbatimLongitude: -85.6365; verbatimCoordinateSystem: Decimal degrees; **Identification:** identifiedBy: AJ Fleming; dateIdentified: 2021; **Event:** samplingProtocol: Reared from the larva of the Sphingidae, Agrius cingulata; verbatimEventDate: 16/Jul/98; **Record Level:** language: en; institutionCode: CNC; collectionCode: Insects; basisOfRecord: PreservedSpecimen
- **Type status:** Paratype. **Occurrence:** catalogNumber: DHJPAR0018724; recordedBy: D.H. Janzen, W. Hallwachs & Manuel Pereira; individualCount: 1; lifeStage: adult; occurrenceStatus: Present; preparations: Pinned; otherCatalogNumbers: ASTAI1371-07| 98-SRNP-8240| BOLD:AAE2876; **Taxon:** scientificName: Metaplagia leahdennisae; phylum: Arthropoda; class: Insecta; order: Diptera; family: Tachinidae; genus: Metaplagia; specificEpithet: leahdennisae; scientificNameAuthorship: Fleming & Wood, 2021; **Location:** continent: Central America; country: Costa Rica; countryCode: CR; stateProvince: Guanacaste; county: Sector Santa Rosa; locality: Area de Conservacion Guanacaste, Quebrada Costa Rica; verbatimElevation: 275; verbatimLatitude: 10.8274; verbatimLongitude: -85.6365; verbatimCoordinateSystem: Decimal degrees; **Identification:** identifiedBy: AJ Fleming; dateIdentified: 2021; **Event:** samplingProtocol: Reared from the larva of the Sphingidae, Agrius cingulata; verbatimEventDate: 6/May/99; **Record Level:** language: en; institutionCode: CNC; collectionCode: Insects; basisOfRecord: PreservedSpecimen
- **Type status:** Paratype. **Occurrence:** catalogNumber: DHJPAR0018727; recordedBy: D.H. Janzen, W. Hallwachs & Jessica DiMauro; individualCount: 1; lifeStage: adult; occurrenceStatus: Present; preparations: Pinned; otherCatalogNumbers: ASTAI1374-07| 98-SRNP-8247| BOLD:AAE2876; **Taxon:** scientificName: Metaplagia leahdennisae; phylum: Arthropoda; class: Insecta; order: Diptera; family: Tachinidae; genus: Metaplagia; specificEpithet: leahdennisae; scientificNameAuthorship: Fleming & Wood, 2021; **Location:** continent: Central America; country: Costa Rica; countryCode: CR; stateProvince: Guanacaste; county: Sector Santa Rosa; locality: Area de Conservacion Guanacaste, Quebrada Costa Rica; verbatimElevation: 275; verbatimLatitude: 10.8274; verbatimLongitude: -85.6365; verbatimCoordinateSystem: Decimal degrees; **Identification:** identifiedBy: AJ Fleming; dateIdentified: 2021; **Event:** samplingProtocol: Reared from the larva of the Sphingidae, Agrius cingulata; verbatimEventDate: 5/May/99; **Record Level:** language: en; institutionCode: CNC; collectionCode: Insects; basisOfRecord: PreservedSpecimen
- **Type status:** Paratype. **Occurrence:** catalogNumber: DHJPAR0052501; recordedBy: D.H. Janzen, W. Hallwachs & Guillermo Pereira; individualCount: 1; lifeStage: adult; occurrenceStatus: Present; preparations: Pinned; otherCatalogNumbers: ASHYM1855-13| 13-SRNP-15519| BOLD:AAE2876; **Taxon:** scientificName: Metaplagia leahdennisae; phylum: Arthropoda; class: Insecta; order: Diptera; family: Tachinidae; genus: Metaplagia; specificEpithet: leahdennisae; scientificNameAuthorship: Fleming & Wood, 2021; **Location:** continent: Central America; country: Costa Rica; countryCode: CR; stateProvince: Guanacaste; county: Sector Santa Rosa; locality: Area de Conservacion Guanacaste, Pueblo Cuajiniquil; verbatimElevation: 5; verbatimLatitude: 10.9404; verbatimLongitude: -85.6804; verbatimCoordinateSystem: Decimal degrees; **Identification:** identifiedBy: AJ Fleming; dateIdentified: 2021; **Event:** samplingProtocol: Reared from the larva of the Sphingidae, Agrius cingulata; verbatimEventDate: 6/Jul/13; **Record Level:** language: en; institutionCode: CNC; collectionCode: Insects; basisOfRecord: PreservedSpecimen
- **Type status:** Paratype. **Occurrence:** catalogNumber: DHJPAR0052509; recordedBy: D.H. Janzen, W. Hallwachs & Guillermo Pereira; individualCount: 1; sex: Female; lifeStage: adult; occurrenceStatus: Present; preparations: Pinned; otherCatalogNumbers: ASHYM1863-13| 13-SRNP-15522| BOLD:AAE2876; **Taxon:** scientificName: Metaplagia leahdennisae; phylum: Arthropoda; class: Insecta; order: Diptera; family: Tachinidae; genus: Metaplagia; specificEpithet: leahdennisae; scientificNameAuthorship: Fleming & Wood, 2021; **Location:** continent: Central America; country: Costa Rica; countryCode: CR; stateProvince: Guanacaste; county: Sector Santa Rosa; locality: Area de Conservacion Guanacaste, Pueblo Cuajiniquil; verbatimElevation: 5; verbatimLatitude: 10.9404; verbatimLongitude: -85.6804; verbatimCoordinateSystem: Decimal degrees; **Identification:** identifiedBy: AJ Fleming; dateIdentified: 2021; **Event:** samplingProtocol: Reared from the larva of the Sphingidae, Agrius cingulata; verbatimEventDate: 4/Jul/13; **Record Level:** language: en; institutionCode: CNC; collectionCode: Insects; basisOfRecord: PreservedSpecimen
- **Type status:** Paratype. **Occurrence:** catalogNumber: DHJPAR0052512; recordedBy: D.H. Janzen, W. Hallwachs & Guillermo Pereira; individualCount: 1; lifeStage: adult; occurrenceStatus: Present; preparations: Pinned; otherCatalogNumbers: ASHYM1866-13| 13-SRNP-15521| BOLD:AAE2876; **Taxon:** scientificName: Metaplagia leahdennisae; phylum: Arthropoda; class: Insecta; order: Diptera; family: Tachinidae; genus: Metaplagia; specificEpithet: leahdennisae; scientificNameAuthorship: Fleming & Wood, 2021; **Location:** continent: Central America; country: Costa Rica; countryCode: CR; stateProvince: Guanacaste; county: Sector Santa Rosa; locality: Area de Conservacion Guanacaste, Pueblo Cuajiniquil; verbatimElevation: 5; verbatimLatitude: 10.9404; verbatimLongitude: -85.6804; verbatimCoordinateSystem: Decimal degrees; **Identification:** identifiedBy: AJ Fleming; dateIdentified: 2021; **Event:** samplingProtocol: Reared from the larva of the Sphingidae, Agrius cingulata; verbatimEventDate: 3/Jul/13; **Record Level:** language: en; institutionCode: CNC; collectionCode: Insects; basisOfRecord: PreservedSpecimen

#### Description

Male (Fig. 2), **head** (Fig. 2c, d): fronto-orbital plate wide, coloration silver with gold tomentum over uppermost 50%, appearing as pale brassy gold along upper 50% of occipital margin of eye; vitta wide, 1/3 width of fronto-orbital plate; ocellar setae lateraloclinate with a slight proclinate skew; with 3 proclinate orbital setae (middle proclinate orbital seta most often shorter and thinner than the other two) and 1 reclinate orbital seta fronto-orbital plate with short black setulae interspersed amongst frontal setae; fronto-orbital setulae extending below lower margin of the pedicel, well into the angle of parafacial; parafacial wholly silver tomentose; palps either slightly spathulate at tips, apically acutely inwardly curved, sparsely setulose along outer margins, inner margin appearing bare; arista dark brown-black. **Thorax** (Fig. 2a, b): black ground color with pale grey to brassy tomentum; 4 postpronotal setae; supra-alar setae 2:3; intra-alar setae 3:3; dorsocentral setae 3:3; acrostichal setae 3:3; katepisternum with 3 setae. Infrasquamal setae present. Scutellum black ground color, with tomentum concolorous with thorax; with five pairs marginal setae; apical scutellar setae 1/2 as long as subapical scutellars, sub-erect, arising above plane of marginal setae; basal scutellar subequal in length to subapical setae; subapical setae convergent, lateral scutellar setae can be convergent or strongly divergent. Legs: black ground color; mid tibia with 2–3 strong anteroventral setae; tarsal claws and pulvilli shorter than last tarsomere. Wings: R_1_ bare, R_4+5_ setulose from node to crossvein r-m. **Abdomen** (Fig. 2a, b): ground color black; ST1+2 glabrous black, pale silver tomentum, occupying anterior 60% of T3–T5; tomentum not extending to ventral surface of abdomen; median marginal setae present on T3 and complete rows on T4 and T5.

**Terminalia** (Fig. 3): posterior margin of sternite 5 with a deeply excavated U-shaped median cleft (Fig. 3d); lateral lobes of sternite apically rounded, with a vestiture of short setae along disc, 3x as long along caudal margin; basal section 1/5 the length of apical lobes. Cerci, in posterior view, medially separated and parallel, not often touching medially, with a few short setae on basal half (Fig. 3a). In lateral view, bowed and sharply tapered apically (Fig. 3b). Surstylus well-developed, stout basally in lateral view, like a broadly rounded triangle, appearing hooked or slightly beaked apically (Fig. 3b, c); in posterior view, basally enlarged and apically straight.

Female (Fig. 4) as in male, except in the following aspects: **head** (Fig. 4c, d): frontal vitta 1/2 width of fronto-orbital plate overall brown with a light gold sheen of tomentum; overall head of females 1/5 wider than that of males; setulae interspersed amongst frontal setae more dense than in males; 3–4 rows of parafacial setulae. Legs (Fig. 4a, b): mid-tibia with 3–4 irregularly-sized strong anteroventral setae.

#### Diagnosis

*Metaplagia leahdennisae* **sp. n.** can be distinguished from all other *Metaplagia* by the following combination of traits: fronto-orbital plate at least 50% gold, with a silver parafacial and setulae on vein R_4+5_ not extending beyond crossvein r-m. *Metaplagia leahdennisae* **sp. n.** can be separated from *Metaplagia occidentalis* by the presence of gold on the fronto-orbital plate. *Metaplagia leahdennisae* **sp. n.** is clearly distinguished by its COI sequence clustered within the Barcode Identification Number (BIN) BOLD:AAE2876.

#### Etymology

*Metaplagia leahdennisae* **sp. n.** is named in honor of Leah Dennis for her many years of coordinating and administrating a plethora of problems and events in the Academic Office of the Department of Biology, University of Pennsylvania, Philadelphia, Pennsylvania.

#### Distribution

Costa Rica, ACG, Guanacaste Province, 5–275 m elevation.

#### Ecology

*Metaplagia leahdennisae* **sp. n.** has been reared eight times from one species of Lepidoptera in the family Sphingidae: *Agrius cingulata* (Fabricius, 1775) in dry forest.

### Metaplagia lindarobinsonae

Fleming & Wood sp. n.

57B47653-B8F8-56A9-B53E-4B22D7C931E9

0F060758-EE04-4BED-8A0A-37E904D0B7C8

#### Materials

- **Type status:** Holotype. **Occurrence:** catalogNumber: DHJPAR0059091; recordedBy: D.H. Janzen, W. Hallwachs & Toby Hammer; individualCount: 1; sex: Male; lifeStage: adult; occurrenceStatus: present; preparations: pinned; otherCatalogNumbers: ACGBA5508-16| 16-SRNP-10173| BOLD:AAD5563; **Taxon:** scientificName: Metaplagia lindarobinsonae; phylum: Arthropoda; class: Insecta; order: Diptera; family: Tachinidae; genus: Metaplagia; specificEpithet: lindarobinsonae; scientificNameAuthorship: Fleming & Wood, 2021; **Location:** continent: Central America; country: Costa Rica; countryCode: CR; stateProvince: Guanacaste; county: Sector Santa Rosa; locality: Area de Conservacion Guanacaste, Area Administrativa; verbatimElevation: 295; verbatimLatitude: 10.8376; verbatimLongitude: -85.6187; verbatimCoordinateSystem: Decimal degrees; **Identification:** identifiedBy: AJ Fleming; dateIdentified: 2021; **Event:** samplingProtocol: Reared from the larva of the Sphingidae, Manduca sexta; verbatimEventDate: 15-Jun-2016; **Record Level:** language: en; institutionCode: CNC; collectionCode: Insects; basisOfRecord: PreservedSpecimen
- **Type status:** Paratype. **Occurrence:** catalogNumber: DHJPAR0018642; recordedBy: D.H. Janzen, W. Hallwachs & gusaneros; individualCount: 1; lifeStage: adult; occurrenceStatus: present; preparations: pinned; otherCatalogNumbers: ASTAI1289-07| 94-SRNP-3161| BOLD:AAD5563; **Taxon:** scientificName: Metaplagia lindarobinsonae; phylum: Arthropoda; class: Insecta; order: Diptera; family: Tachinidae; genus: Metaplagia; specificEpithet: lindarobinsonae; scientificNameAuthorship: Fleming & Wood, 2021; **Location:** continent: Central America; country: Costa Rica; countryCode: CR; stateProvince: Guanacaste; county: Sector Santa Rosa; locality: Area de Conservacion Guanacaste, Bosque San Emilio; verbatimElevation: 300; verbatimLatitude: 10.8439; verbatimLongitude: -85.6138; verbatimCoordinateSystem: Decimal degrees; **Identification:** identifiedBy: AJ Fleming; dateIdentified: 2021; **Event:** samplingProtocol: Reared from the larva of the Sphingidae, Manduca sextaDHJ03; verbatimEventDate: 21-Jun-1994; **Record Level:** language: en; institutionCode: CNC; collectionCode: Insects; basisOfRecord: PreservedSpecimen
- **Type status:** Paratype. **Occurrence:** catalogNumber: DHJPAR0018643; recordedBy: D.H. Janzen, W. Hallwachs & gusaneros; individualCount: 1; lifeStage: adult; occurrenceStatus: present; preparations: pinned; otherCatalogNumbers: ASTAI1290-07| 94-SRNP-3160| BOLD:AAD5563; **Taxon:** scientificName: Metaplagia lindarobinsonae; phylum: Arthropoda; class: Insecta; order: Diptera; family: Tachinidae; genus: Metaplagia; specificEpithet: lindarobinsonae; scientificNameAuthorship: Fleming & Wood, 2021; **Location:** continent: Central America; country: Costa Rica; countryCode: CR; stateProvince: Guanacaste; county: Sector Santa Rosa; locality: Area de Conservacion Guanacaste, Bosque San Emilio; verbatimElevation: 300; verbatimLatitude: 10.8439; verbatimLongitude: -85.6138; verbatimCoordinateSystem: Decimal degrees; **Identification:** identifiedBy: AJ Fleming; dateIdentified: 2021; **Event:** samplingProtocol: Reared from the larva of the Sphingidae, Manduca sextaDHJ03; verbatimEventDate: 30-Jun-1994; **Record Level:** language: en; institutionCode: CNC; collectionCode: Insects; basisOfRecord: PreservedSpecimen
- **Type status:** Paratype. **Occurrence:** catalogNumber: DHJPAR0018644; recordedBy: D.H. Janzen, W. Hallwachs & gusaneros; individualCount: 1; sex: Female; lifeStage: adult; occurrenceStatus: present; preparations: pinned; otherCatalogNumbers: ASTAI1291-07| 89-SRNP-173| BOLD:AAD5563; **Taxon:** scientificName: Metaplagia lindarobinsonae; phylum: Arthropoda; class: Insecta; order: Diptera; family: Tachinidae; genus: Metaplagia; specificEpithet: lindarobinsonae; scientificNameAuthorship: Fleming & Wood, 2021; **Location:** continent: Central America; country: Costa Rica; countryCode: CR; stateProvince: Guanacaste; county: Sector Pocosol; locality: Area de Conservacion Guanacaste, Quebrada Aserradero; verbatimElevation: 160; verbatimLatitude: 10.8986; verbatimLongitude: -85.5642; verbatimCoordinateSystem: Decimal degrees; **Identification:** identifiedBy: AJ Fleming; dateIdentified: 2021; **Event:** samplingProtocol: Reared from the larva of the Sphingidae, Manduca sextaDHJ03; verbatimEventDate: 22-May-1990; **Record Level:** language: en; institutionCode: CNC; collectionCode: Insects; basisOfRecord: PreservedSpecimen
- **Type status:** Paratype. **Occurrence:** catalogNumber: DHJPAR0018645; recordedBy: D.H. Janzen, W. Hallwachs & gusaneros; individualCount: 1; lifeStage: adult; occurrenceStatus: present; preparations: pinned; otherCatalogNumbers: ASTAI1292-07| 89-SRNP-173| BOLD:AAD5563; **Taxon:** scientificName: Metaplagia lindarobinsonae; phylum: Arthropoda; class: Insecta; order: Diptera; family: Tachinidae; genus: Metaplagia; specificEpithet: lindarobinsonae; scientificNameAuthorship: Fleming & Wood, 2021; **Location:** continent: Central America; country: Costa Rica; countryCode: CR; stateProvince: Guanacaste; county: Sector Pocosol; locality: Area de Conservacion Guanacaste, Quebrada Aserradero; verbatimElevation: 160; verbatimLatitude: 10.8986; verbatimLongitude: -85.5642; verbatimCoordinateSystem: Decimal degrees; **Identification:** identifiedBy: AJ Fleming; dateIdentified: 2021; **Event:** samplingProtocol: Reared from the larva of the Sphingidae, Manduca sextaDHJ03; verbatimEventDate: 22-May-1990; **Record Level:** language: en; institutionCode: CNC; collectionCode: Insects; basisOfRecord: PreservedSpecimen
- **Type status:** Paratype. **Occurrence:** catalogNumber: DHJPAR0019809; recordedBy: D.H. Janzen, W. Hallwachs & Guillermo Pereira; individualCount: 1; sex: Male; lifeStage: adult; occurrenceStatus: present; preparations: pinned; otherCatalogNumbers: ASTAB357-07| 06-SRNP-17663| BOLD:AAD5563; **Taxon:** scientificName: Metaplagia lindarobinsonae; phylum: Arthropoda; class: Insecta; order: Diptera; family: Tachinidae; genus: Metaplagia; specificEpithet: lindarobinsonae; scientificNameAuthorship: Fleming & Wood, 2021; **Location:** continent: Central America; country: Costa Rica; countryCode: CR; stateProvince: Guanacaste; county: Sector Horizontes; locality: Area de Conservacion Guanacaste, Vado Esperanza; verbatimElevation: 85; verbatimLatitude: 10.7894; verbatimLongitude: -85.551; verbatimCoordinateSystem: Decimal degrees; **Identification:** identifiedBy: AJ Fleming; dateIdentified: 2021; **Event:** samplingProtocol: Reared from the larva of the Sphingidae, Manduca sextaDHJ02; verbatimEventDate: 04-Jun-2007; **Record Level:** language: en; institutionCode: CNC; collectionCode: Insects; basisOfRecord: PreservedSpecimen
- **Type status:** Paratype. **Occurrence:** catalogNumber: DHJPAR0059089; recordedBy: D.H. Janzen, W. Hallwachs & Toby Hammer; individualCount: 1; lifeStage: adult; occurrenceStatus: present; preparations: pinned; otherCatalogNumbers: ACGBA5506-16| 16-SRNP-10173| BOLD:AAD5563; **Taxon:** scientificName: Metaplagia lindarobinsonae; phylum: Arthropoda; class: Insecta; order: Diptera; family: Tachinidae; genus: Metaplagia; specificEpithet: lindarobinsonae; scientificNameAuthorship: Fleming & Wood, 2021; **Location:** continent: Central America; country: Costa Rica; countryCode: CR; stateProvince: Guanacaste; county: Sector Santa Rosa; locality: Area de Conservacion Guanacaste, Area Administrativa; verbatimElevation: 295; verbatimLatitude: 10.8376; verbatimLongitude: -85.6187; verbatimCoordinateSystem: Decimal degrees; **Identification:** identifiedBy: AJ Fleming; dateIdentified: 2021; **Event:** samplingProtocol: Reared from the larva of the Sphingidae, Manduca sexta; verbatimEventDate: 15-Jun-2016; **Record Level:** language: en; institutionCode: CNC; collectionCode: Insects; basisOfRecord: PreservedSpecimen
- **Type status:** Paratype. **Occurrence:** catalogNumber: DHJPAR0059090; recordedBy: D.H. Janzen, W. Hallwachs & Toby Hammer; individualCount: 1; lifeStage: adult; occurrenceStatus: present; preparations: pinned; otherCatalogNumbers: ACGBA5507-16| 16-SRNP-10173| BOLD:AAD5563; **Taxon:** scientificName: Metaplagia lindarobinsonae; phylum: Arthropoda; class: Insecta; order: Diptera; family: Tachinidae; genus: Metaplagia; specificEpithet: lindarobinsonae; scientificNameAuthorship: Fleming & Wood, 2021; **Location:** continent: Central America; country: Costa Rica; countryCode: CR; stateProvince: Guanacaste; county: Sector Santa Rosa; locality: Area de Conservacion Guanacaste, Area Administrativa; verbatimElevation: 295; verbatimLatitude: 10.8376; verbatimLongitude: -85.6187; verbatimCoordinateSystem: Decimal degrees; **Identification:** identifiedBy: AJ Fleming; dateIdentified: 2021; **Event:** samplingProtocol: Reared from the larva of the Sphingidae, Manduca sexta; verbatimEventDate: 15-Jun-2016; **Record Level:** language: en; institutionCode: CNC; collectionCode: Insects; basisOfRecord: PreservedSpecimen
- **Type status:** Paratype. **Occurrence:** catalogNumber: DHJPAR0018641; recordedBy: D.H. Janzen, W. Hallwachs & gusaneros; individualCount: 1; lifeStage: adult; occurrenceStatus: present; preparations: pinned; otherCatalogNumbers: ASTAI1288-07| 94-SRNP-3160| BOLD:AAD5563; **Taxon:** scientificName: Metaplagia lindarobinsonae; phylum: Arthropoda; class: Insecta; order: Diptera; family: Tachinidae; genus: Metaplagia; specificEpithet: lindarobinsonae; scientificNameAuthorship: Fleming & Wood, 2021; **Location:** continent: Central America; country: Costa Rica; countryCode: CR; stateProvince: Guanacaste; county: Sector Santa Rosa; locality: Area de Conservacion Guanacaste, Bosque San Emilio; verbatimElevation: 300; verbatimLatitude: 10.8439; verbatimLongitude: -85.6138; verbatimCoordinateSystem: Decimal degrees; **Identification:** identifiedBy: AJ Fleming; dateIdentified: 2021; **Event:** samplingProtocol: Reared from the larva of the Sphingidae, Manduca sextaDHJ03; verbatimEventDate: 30-Jun-1994; **Record Level:** language: en; institutionCode: CNC; collectionCode: Insects; basisOfRecord: PreservedSpecimen
- **Type status:** Paratype. **Occurrence:** catalogNumber: DHJPAR0059092; recordedBy: D.H. Janzen, W. Hallwachs & Toby Hammer; individualCount: 1; lifeStage: adult; occurrenceStatus: present; preparations: pinned; otherCatalogNumbers: ACGBA5509-16| 16-SRNP-10173| BOLD:AAD5563; **Taxon:** scientificName: Metaplagia lindarobinsonae; phylum: Arthropoda; class: Insecta; order: Diptera; family: Tachinidae; genus: Metaplagia; specificEpithet: lindarobinsonae; scientificNameAuthorship: Fleming & Wood, 2021; **Location:** continent: Central America; country: Costa Rica; countryCode: CR; stateProvince: Guanacaste; county: Sector Santa Rosa; locality: Area de Conservacion Guanacaste, Area Administrativa; verbatimElevation: 295; verbatimLatitude: 10.8376; verbatimLongitude: -85.6187; verbatimCoordinateSystem: Decimal degrees; **Identification:** identifiedBy: AJ Fleming; dateIdentified: 2021; **Event:** samplingProtocol: Reared from the larva of the Sphingidae, Manduca sexta; verbatimEventDate: 15-Jun-2016; **Record Level:** language: en; institutionCode: CNC; collectionCode: Insects; basisOfRecord: PreservedSpecimen
- **Type status:** Paratype. **Occurrence:** catalogNumber: DHJPAR0059093; recordedBy: D.H. Janzen, W. Hallwachs & Toby Hammer; individualCount: 1; lifeStage: adult; occurrenceStatus: present; preparations: pinned; otherCatalogNumbers: ACGBA5510-16| 16-SRNP-10173| BOLD:AAD5563; **Taxon:** scientificName: Metaplagia lindarobinsonae; phylum: Arthropoda; class: Insecta; order: Diptera; family: Tachinidae; genus: Metaplagia; specificEpithet: lindarobinsonae; scientificNameAuthorship: Fleming & Wood, 2021; **Location:** continent: Central America; country: Costa Rica; countryCode: CR; stateProvince: Guanacaste; county: Sector Santa Rosa; locality: Area de Conservacion Guanacaste, Area Administrativa; verbatimElevation: 295; verbatimLatitude: 10.8376; verbatimLongitude: -85.6187; verbatimCoordinateSystem: Decimal degrees; **Identification:** identifiedBy: AJ Fleming; dateIdentified: 2021; **Event:** samplingProtocol: Reared from the larva of the Sphingidae, Manduca sexta; verbatimEventDate: 15-Jun-2016; **Record Level:** language: en; institutionCode: CNC; collectionCode: Insects; basisOfRecord: PreservedSpecimen
- **Type status:** Paratype. **Occurrence:** catalogNumber: DHJPAR0059094; recordedBy: D.H. Janzen, W. Hallwachs & Toby Hammer; individualCount: 1; lifeStage: adult; occurrenceStatus: present; preparations: pinned; otherCatalogNumbers: ACGBA5511-16| 16-SRNP-10173| BOLD:AAD5563; **Taxon:** scientificName: Metaplagia lindarobinsonae; phylum: Arthropoda; class: Insecta; order: Diptera; family: Tachinidae; genus: Metaplagia; specificEpithet: lindarobinsonae; scientificNameAuthorship: Fleming & Wood, 2021; **Location:** continent: Central America; country: Costa Rica; countryCode: CR; stateProvince: Guanacaste; county: Sector Santa Rosa; locality: Area de Conservacion Guanacaste, Area Administrativa; verbatimElevation: 295; verbatimLatitude: 10.8376; verbatimLongitude: -85.6187; verbatimCoordinateSystem: Decimal degrees; **Identification:** identifiedBy: AJ Fleming; dateIdentified: 2021; **Event:** samplingProtocol: Reared from the larva of the Sphingidae, Manduca sexta; verbatimEventDate: 15-Jun-2016; **Record Level:** language: en; institutionCode: CNC; collectionCode: Insects; basisOfRecord: PreservedSpecimen
- **Type status:** Paratype. **Occurrence:** catalogNumber: DHJPAR0059095; recordedBy: D.H. Janzen, W. Hallwachs & Toby Hammer; individualCount: 1; lifeStage: adult; occurrenceStatus: present; preparations: pinned; otherCatalogNumbers: ACGBA5512-16| 16-SRNP-10173| BOLD:AAD5563; **Taxon:** scientificName: Metaplagia lindarobinsonae; phylum: Arthropoda; class: Insecta; order: Diptera; family: Tachinidae; genus: Metaplagia; specificEpithet: lindarobinsonae; scientificNameAuthorship: Fleming & Wood, 2021; **Location:** continent: Central America; country: Costa Rica; countryCode: CR; stateProvince: Guanacaste; county: Sector Santa Rosa; locality: Area de Conservacion Guanacaste, Area Administrativa; verbatimElevation: 295; verbatimLatitude: 10.8376; verbatimLongitude: -85.6187; verbatimCoordinateSystem: Decimal degrees; **Identification:** identifiedBy: AJ Fleming; dateIdentified: 2021; **Event:** samplingProtocol: Reared from the larva of the Sphingidae, Manduca sexta; verbatimEventDate: 15-Jun-2016; **Record Level:** language: en; institutionCode: CNC; collectionCode: Insects; basisOfRecord: PreservedSpecimen
- **Type status:** Paratype. **Occurrence:** catalogNumber: DHJPAR0059096; recordedBy: D.H. Janzen, W. Hallwachs & Toby Hammer; individualCount: 1; lifeStage: adult; occurrenceStatus: present; preparations: pinned; otherCatalogNumbers: ACGBA5513-16| 16-SRNP-10173| BOLD:AAD5563; **Taxon:** scientificName: Metaplagia lindarobinsonae; phylum: Arthropoda; class: Insecta; order: Diptera; family: Tachinidae; genus: Metaplagia; specificEpithet: lindarobinsonae; scientificNameAuthorship: Fleming & Wood, 2021; **Location:** continent: Central America; country: Costa Rica; countryCode: CR; stateProvince: Guanacaste; county: Sector Santa Rosa; locality: Area de Conservacion Guanacaste, Area Administrativa; verbatimElevation: 295; verbatimLatitude: 10.8376; verbatimLongitude: -85.6187; verbatimCoordinateSystem: Decimal degrees; **Identification:** identifiedBy: AJ Fleming; dateIdentified: 2021; **Event:** samplingProtocol: Reared from the larva of the Sphingidae, Manduca sexta; verbatimEventDate: 15-Jun-2016; **Record Level:** language: en; institutionCode: CNC; collectionCode: Insects; basisOfRecord: PreservedSpecimen
- **Type status:** Paratype. **Occurrence:** catalogNumber: DHJPAR0059097; recordedBy: D.H. Janzen, W. Hallwachs & Toby Hammer; individualCount: 1; lifeStage: adult; occurrenceStatus: present; preparations: pinned; otherCatalogNumbers: ACGBA5514-16| 16-SRNP-10173| BOLD:AAD5563; **Taxon:** scientificName: Metaplagia lindarobinsonae; phylum: Arthropoda; class: Insecta; order: Diptera; family: Tachinidae; genus: Metaplagia; specificEpithet: lindarobinsonae; scientificNameAuthorship: Fleming & Wood, 2021; **Location:** continent: Central America; country: Costa Rica; countryCode: CR; stateProvince: Guanacaste; county: Sector Santa Rosa; locality: Area de Conservacion Guanacaste, Area Administrativa; verbatimElevation: 295; verbatimLatitude: 10.8376; verbatimLongitude: -85.6187; verbatimCoordinateSystem: Decimal degrees; **Identification:** identifiedBy: AJ Fleming; dateIdentified: 2021; **Event:** samplingProtocol: Reared from the larva of the Sphingidae, Manduca sexta; verbatimEventDate: 15-Jun-2016; **Record Level:** language: en; institutionCode: CNC; collectionCode: Insects; basisOfRecord: PreservedSpecimen
- **Type status:** Paratype. **Occurrence:** catalogNumber: DHJPAR0059098; recordedBy: D.H. Janzen, W. Hallwachs & Toby Hammer; individualCount: 1; lifeStage: adult; occurrenceStatus: present; preparations: pinned; otherCatalogNumbers: ACGBA5515-16| 16-SRNP-10173| BOLD:AAD5563; **Taxon:** scientificName: Metaplagia lindarobinsonae; phylum: Arthropoda; class: Insecta; order: Diptera; family: Tachinidae; genus: Metaplagia; specificEpithet: lindarobinsonae; scientificNameAuthorship: Fleming & Wood, 2021; **Location:** continent: Central America; country: Costa Rica; countryCode: CR; stateProvince: Guanacaste; county: Sector Santa Rosa; locality: Area de Conservacion Guanacaste, Area Administrativa; verbatimElevation: 295; verbatimLatitude: 10.8376; verbatimLongitude: -85.6187; verbatimCoordinateSystem: Decimal degrees; **Identification:** identifiedBy: AJ Fleming; dateIdentified: 2021; **Event:** samplingProtocol: Reared from the larva of the Sphingidae, Manduca sexta; verbatimEventDate: 15-Jun-2016; **Record Level:** language: en; institutionCode: CNC; collectionCode: Insects; basisOfRecord: PreservedSpecimen
- **Type status:** Paratype. **Occurrence:** catalogNumber: DHJPAR0059440; recordedBy: D.H. Janzen, W. Hallwachs & Winnie Hallwachs; individualCount: 1; lifeStage: adult; occurrenceStatus: present; preparations: pinned; otherCatalogNumbers: ACGBA5857-16| 16-SRNP-10201| BOLD:AAD5563; **Taxon:** scientificName: Metaplagia lindarobinsonae; phylum: Arthropoda; class: Insecta; order: Diptera; family: Tachinidae; genus: Metaplagia; specificEpithet: lindarobinsonae; scientificNameAuthorship: Fleming & Wood, 2021; **Location:** continent: Central America; country: Costa Rica; countryCode: CR; stateProvince: Guanacaste; county: Sector Santa Rosa; locality: Area de Conservacion Guanacaste, Area Administrativa; verbatimElevation: 295; verbatimLatitude: 10.8376; verbatimLongitude: -85.6187; verbatimCoordinateSystem: Decimal degrees; **Identification:** identifiedBy: AJ Fleming; dateIdentified: 2021; **Event:** samplingProtocol: Reared from the larva of the Sphingidae, Manduca sexta; verbatimEventDate: 22-Jun-2016; **Record Level:** language: en; institutionCode: CNC; collectionCode: Insects; basisOfRecord: PreservedSpecimen
- **Type status:** Paratype. **Occurrence:** catalogNumber: DHJPAR0060750; recordedBy: D.H. Janzen, W. Hallwachs & Toby Hammer; individualCount: 1; lifeStage: adult; occurrenceStatus: present; preparations: pinned; otherCatalogNumbers: ACGBA7171-17| 16-SRNP-10172| BOLD:AAD5563; **Taxon:** scientificName: Metaplagia lindarobinsonae; phylum: Arthropoda; class: Insecta; order: Diptera; family: Tachinidae; genus: Metaplagia; specificEpithet: lindarobinsonae; scientificNameAuthorship: Fleming & Wood, 2021; **Location:** continent: Central America; country: Costa Rica; countryCode: CR; stateProvince: Guanacaste; county: Sector Santa Rosa; locality: Area de Conservacion Guanacaste, Area Administrativa; verbatimElevation: 295; verbatimLatitude: 10.8376; verbatimLongitude: -85.6187; verbatimCoordinateSystem: Decimal degrees; **Identification:** identifiedBy: AJ Fleming; dateIdentified: 2021; **Event:** samplingProtocol: Reared from the larva of the Sphingidae, Manduca sexta; verbatimEventDate: May-2017; **Record Level:** language: en; institutionCode: CNC; collectionCode: Insects; basisOfRecord: PreservedSpecimen
- **Type status:** Paratype. **Occurrence:** catalogNumber: DHJPAR0060751; recordedBy: D.H. Janzen, W. Hallwachs & Toby Hammer; individualCount: 1; lifeStage: adult; occurrenceStatus: present; preparations: pinned; otherCatalogNumbers: ACGBA7172-17| 16-SRNP-10173| BOLD:AAD5563; **Taxon:** scientificName: Metaplagia lindarobinsonae; phylum: Arthropoda; class: Insecta; order: Diptera; family: Tachinidae; genus: Metaplagia; specificEpithet: lindarobinsonae; scientificNameAuthorship: Fleming & Wood, 2021; **Location:** continent: Central America; country: Costa Rica; countryCode: CR; stateProvince: Guanacaste; county: Sector Santa Rosa; locality: Area de Conservacion Guanacaste, Area Administrativa; verbatimElevation: 295; verbatimLatitude: 10.8376; verbatimLongitude: -85.6187; verbatimCoordinateSystem: Decimal degrees; **Identification:** identifiedBy: AJ Fleming; dateIdentified: 2021; **Event:** samplingProtocol: Reared from the larva of the Sphingidae, Manduca sexta; verbatimEventDate: 15-Jun-2016; **Record Level:** language: en; institutionCode: CNC; collectionCode: Insects; basisOfRecord: PreservedSpecimen

#### Description

Male (Fig. 5), **head** (Fig. 5c, d): fronto-orbital plate wide, coloration silver to pale grey; vitta wide, 1/3 width of fronto-orbital plate ocellar setae lateraloclinate with a slight proclinate skew; with 3–4 proclinate orbital setae (middle proclinate orbital seta most often shorter and thinner than the other two) and 1 reclinate orbital seta fronto-orbital plate with short black setulae interspersed amongst frontal setae; fronto-orbital setulae extending below lower margin of pedicel, well into angle of parafacial; parafacial wholly silver tomentose with a distinct row of parafacial setulae along inner margin of parafacial approaching facial ridge, seemingly continuous with fronto-orbital setulae; palps either slightly spathulate at tips, apically acutely inwardly curved, extremely sparsely setulose along inner margin, remainder appearing bare; arista dark brown-black. **Thorax** (Fig. 5a, b): black ground color with pale grey tomentum; 3–4 postpronotal setae; supra-alar setae 2:3; intra-alar setae 3:3; dorsocentral setae 3:3; acrostichal setae 3:3; katepisternum with 3 setae. Infrasquamal setae present. Scutellum black ground color, with tomentum concolorous with thorax; with five pairs marginal setae; apical scutellar setae 1/2 as long as subapical scutellars, sub-erect, arising above plane of marginal setae; basal scutellar subequal in length to subapical setae; subapical setae convergent, lateral scutellar setae can be convergent or strongly divergent. Legs: black ground color; mid tibia with 2–3 strong anteroventral setae; tarsal claws and pulvilli shorter than last tarsomere. Wings: R_1_ bare, R_4+5_ setulose from node up to, but not beyond, crossvein r-m. **Abdomen** (Fig. 5a, b): ground color black; ST1+2 glabrous black, pale silver tomentum occupying anterior 50% of T3–T5; tomentum not extending to ventral surface of abdomen; median marginal setae present on T3, and complete rows on T4 and T5.

**Terminalia** (Fig. 6): posterior margin of sternite 5 with a deep and narrow excavated U-shaped median cleft (Fig. 6d); lateral lobes of sternite apically rounded, with a vestiture of short setae along disc, 3x as long along caudal margin; basal section 1/3 the length of apical lobes. Cerci, in posterior view, medially separated and parallel not touching medially, with a few short setae on basal half (Fig. 6a); in lateral view, bowed and sharply tapered to a point apically (Fig. 6b, c). Surstylus well-developed, stout basally in lateral view, rounded basally and appearing hooked or slightly beaked apically; in posterior view, basally enlarged and apically straight.

Female (Fig. 7) as in male, except in the following aspects: **head** (Fig. 7c, d): fronto-orbital just under 1/2 of head width, vitta 1/3 width of fronto-orbital plate; overall head of females 1/5 wider than that of males; legs (Fig. 7b): mid-tibia with 3–4 irregularly-sized strong anteroventral setae.

#### Diagnosis

*Metaplagia lindarobinsonae* **sp. n.** can be distinguished from all other *Metaplagia* by the following combination of traits: frontal vitta indistinct around ocellar triangle, infrasquamal setae present, fronto-orbital plate at pale gray or silver, with a silver parafacial and setulae on vein R_4+5_ not extending beyond crossvein r-m, surstylus sharp at tip. *Metaplagia lindarobinsonae* **sp. n.** can be separated from *Metaplagia occidentalis* by the presence of infrasquamal setae and from *Metaplagia svetlanakozikae* **sp. n.** by the presence of a distinct row of setulae along the facial ridge. *Metaplagia lindarobinsonae* **sp. n.** is clearly distinguished by its COI sequence clustered within the Barcode Identification Number (BIN) BOLD:AAD5563.

#### Etymology

*Metaplagia lindarobinsonae* **sp. n.** is named in honor of Linda Robinson for her many years of coordinating and administrating a plethora of problems and events for the undergraduate biology teaching laboratories of the Department of Biology, University of Pennsylvania, Philadelphia, Pennsylvania.

#### Distribution

Costa Rica, ACG, Guanacaste Province, 85–300 m elevation.

#### Ecology

*Metaplagia lindarobinsonae* **sp. n.** has been reared 17 times from two species of Lepidoptera in the family Sphingidae: *Manduca sexta* (Linnaeus, 1763), *Manduca sexta*DHJ02 and *Manduca sexta*DHJ03, in dry forest.

### Metaplagia paulinesaribasae

Fleming & Wood sp. n.

51CEEF49-5D09-52A1-A386-1BB5632446EE

E421A1D5-215A-4353-BCA6-20B42098E5C6

#### Materials

- **Type status:** Holotype. **Occurrence:** catalogNumber: DHJPAR0018646; recordedBy: D.H. Janzen, W. Hallwachs; individualCount: 1; sex: Male; lifeStage: adult; occurrenceStatus: present; preparations: pinned; otherCatalogNumbers: ASTAI1293-07| 82-SRNP-382| BOLD:AAX4230; **Taxon:** scientificName: Metaplagia paulinesaribasae; phylum: Arthropoda; class: Insecta; order: Diptera; family: Tachinidae; genus: Metaplagia; specificEpithet: paulinesaribasae; scientificNameAuthorship: Fleming & Wood, 2021; **Location:** continent: Central America; country: Costa Rica; countryCode: CR; stateProvince: Guanacaste; county: Sector Santa Rosa; locality: Area de Conservacion Guanacaste, Cafetal; verbatimElevation: 280; verbatimLatitude: 10.8583; verbatimLongitude: -85.6109; verbatimCoordinateSystem: Decimal degrees; **Identification:** identifiedBy: AJ Fleming; dateIdentified: 2021; **Event:** samplingProtocol: Reared from the larva of the Sphingidae, Manduca lefeburii; verbatimEventDate: 24-Aug-1982; **Record Level:** language: en; institutionCode: CNC; collectionCode: Insects; basisOfRecord: PreservedSpecimen

#### Description

Male (Fig. 8), **head** (Fig. 8c, d): fronto-orbital plate wide, gold tomentum over its entirety; vitta wide, 1/5 width of fronto-orbital plate; ocellar setae lateraloclinate with a slight proclinate skew; with 2 proclinate orbital setae and 1 reclinate orbital seta fronto-orbital plate with short black setulae interspersed amongst frontal setae; fronto-orbital setulae extending below lower margin of the pedicel, well into angle of parafacial; parafacial wholly gold tomentose; palps either slightly spathulate at tips, apically acutely inwardly curved, 3–4 setulae along lower margin, inner margin appearing bare; arista light brown-dark orange, distinctly lighter color than postpedicel. **Thorax** (Fig. 8a, b): black ground color with pale brassy tomentum; 4 postpronotal setae; supra-alar setae 2:3; intra-alar setae 3:3; dorsocentral setae 3:3; acrostichal setae 3:3; katepisternum with 3 setae. Infrasquamal setae present. Scutellum black ground color, with tomentum concolorous with thorax; with five pairs marginal setae; apical scutellar setae 1/2 as long as subapical scutellars, sub-erect, arising above plane of marginal setae; basal scutellar subequal in length to subapical setae; subapical setae convergent, lateral scutellar setae can be convergent or strongly divergent; 2 pairs of scutellar discal setae set wider apart than subapical setae. Legs: black ground color; mid tibia with 2–3 strong anteroventral setae; tarsal claws and pulvilli shorter than last tarsomere. Wings: R_1_ bare, R_4+5_ setulose from node to crossvein r-m. **Abdomen** (Fig. 8a, b): ground color black; ST1+2 glabrous black, silver-pale brassy tomentum occupying anterior 90% of T3–T5; tomentum thin to absent along ventral surface of abdomen; median marginal setae present on T3 and complete rows on T4 and T5.

Terminalia: holotype male not dissected.

Female: unknown at this time.

#### Diagnosis

*Metaplagia paulinesaribasae* **sp. n.** can be easily distinguished from all other *Metaplagia* by the following combination of traits: both fronto-orbital and parafacial gold and setulae on vein R_4+5_ not extending beyond crossvein r-m. It is distinguishable from all other congeners by the presence of an entirely gold fronto-orbital plate and parafacial. *Metaplagia paulinesaribasae* **sp. n.** is clearly distinguished by its COI sequence clustered within the Barcode Identification Number (BIN) BOLD:AAX4230.

#### Etymology

*Metaplagia paulinesaribasae* **sp. n.** is named in honor of Pauline Saribas for her many years of coordinating and administrating a plethora of problems and events in the Academic Office of the Department of Biology, University of Pennsylvania, Philadelphia, Pennsylvania.

#### Distribution

Costa Rica, ACG, Guanacaste Province, 280 m elevation.

#### Ecology

*Metaplagia paulinesaribasae* **sp. n.** has been reared once from one species of Lepidoptera in the family Sphingidae: *Manduca lefeburii* (Guérin-Méneville, 1844), in dry forest.

### Metaplagia robinsherwoodae

Fleming & Wood sp. n.

5317B410-6580-5283-89B4-BBDF8FB9C4CE

F6B26F03-C22B-4168-A675-39D20DDFA87C

#### Materials

- **Type status:** Holotype. **Occurrence:** catalogNumber: DHJPAR0018640; recordedBy: D.H. Janzen, W. Hallwachs & gusaneros; individualCount: 1; sex: Male; lifeStage: adult; occurrenceStatus: present; preparations: pinned; otherCatalogNumbers: ASTAI1287-07| 92-SRNP-2162| BOLD:AAB3286; **Taxon:** scientificName: Metaplagia robinsherwoodae; phylum: Arthropoda; class: Insecta; order: Diptera; family: Tachinidae; genus: Metaplagia; specificEpithet: robinsherwoodae; scientificNameAuthorship: Fleming & Wood, 2021; **Location:** continent: Central America; country: Costa Rica; countryCode: CR; stateProvince: Guanacaste; county: Sector Santa Rosa; locality: Area de Conservacion Guanacaste, Cafetal; verbatimElevation: 280; verbatimLatitude: 10.8583; verbatimLongitude: -85.6109; verbatimCoordinateSystem: Decimal degrees; **Identification:** identifiedBy: AJ Fleming; dateIdentified: 2021; **Event:** samplingProtocol: Reared from the larva of the Sphingidae, Manduca rustica; verbatimEventDate: 03-Jul-1992; **Record Level:** language: en; institutionCode: CNC; collectionCode: Insects; basisOfRecord: PreservedSpecimen
- **Type status:** Paratype. **Occurrence:** catalogNumber: DHJPAR0018629; recordedBy: D.H. Janzen, W. Hallwachs & Ruth Franco; individualCount: 1; lifeStage: adult; occurrenceStatus: present; preparations: pinned; otherCatalogNumbers: ASTAI1276-07| 98-SRNP-8635| BOLD:AAB3286; **Taxon:** scientificName: Metaplagia robinsherwoodae; phylum: Arthropoda; class: Insecta; order: Diptera; family: Tachinidae; genus: Metaplagia; specificEpithet: robinsherwoodae; scientificNameAuthorship: Fleming & Wood, 2021; **Location:** continent: Central America; country: Costa Rica; countryCode: CR; stateProvince: Guanacaste; county: Sector Santa Rosa; locality: Area de Conservacion Guanacaste, Quebrada Cebollines; verbatimElevation: 270; verbatimLatitude: 10.8222; verbatimLongitude: -85.6434; verbatimCoordinateSystem: Decimal degrees; **Identification:** identifiedBy: AJ Fleming; dateIdentified: 2021; **Event:** samplingProtocol: Reared from the larva of the Sphingidae, Manduca rustica; verbatimEventDate: 24-Jul-1998; **Record Level:** language: en; institutionCode: CNC; collectionCode: Insects; basisOfRecord: PreservedSpecimen
- **Type status:** Paratype. **Occurrence:** catalogNumber: DHJPAR0018632; recordedBy: D.H. Janzen, W. Hallwachs & Guillermo Pereira; individualCount: 1; lifeStage: adult; occurrenceStatus: present; preparations: pinned; otherCatalogNumbers: ASTAI1279-07| 98-SRNP-8715| BOLD:AAB3286; **Taxon:** scientificName: Metaplagia robinsherwoodae; phylum: Arthropoda; class: Insecta; order: Diptera; family: Tachinidae; genus: Metaplagia; specificEpithet: robinsherwoodae; scientificNameAuthorship: Fleming & Wood, 2021; **Location:** continent: Central America; country: Costa Rica; countryCode: CR; stateProvince: Guanacaste; county: Sector Santa Rosa; locality: Area de Conservacion Guanacaste, Bosque San Emilio; verbatimElevation: 300; verbatimLatitude: 10.8439; verbatimLongitude: -85.6138; verbatimCoordinateSystem: Decimal degrees; **Identification:** identifiedBy: AJ Fleming; dateIdentified: 2021; **Event:** samplingProtocol: Reared from the larva of the Sphingidae, Manduca rustica; verbatimEventDate: 22-Jul-1998; **Record Level:** language: en; institutionCode: CNC; collectionCode: Insects; basisOfRecord: PreservedSpecimen
- **Type status:** Paratype. **Occurrence:** catalogNumber: DHJPAR0018633; recordedBy: D.H. Janzen, W. Hallwachs & gusaneros; individualCount: 1; lifeStage: adult; occurrenceStatus: present; preparations: pinned; otherCatalogNumbers: ASTAI1280-07| 93-SRNP-1643| BOLD:AAB3286; **Taxon:** scientificName: Metaplagia robinsherwoodae; phylum: Arthropoda; class: Insecta; order: Diptera; family: Tachinidae; genus: Metaplagia; specificEpithet: robinsherwoodae; scientificNameAuthorship: Fleming & Wood, 2021; **Location:** continent: Central America; country: Costa Rica; countryCode: CR; stateProvince: Guanacaste; county: Sector Santa Rosa; locality: Area de Conservacion Guanacaste, Area Administrativa; verbatimElevation: 295; verbatimLatitude: 10.8376; verbatimLongitude: -85.6187; verbatimCoordinateSystem: Decimal degrees; **Identification:** identifiedBy: AJ Fleming; dateIdentified: 2021; **Event:** samplingProtocol: Reared from the larva of the Sphingidae, Manduca rustica; verbatimEventDate: 03-Jul-1993; **Record Level:** language: en; institutionCode: CNC; collectionCode: Insects; basisOfRecord: PreservedSpecimen
- **Type status:** Paratype. **Occurrence:** catalogNumber: DHJPAR0018634; recordedBy: D.H. Janzen, W. Hallwachs & gusaneros; individualCount: 1; lifeStage: adult; occurrenceStatus: present; preparations: pinned; otherCatalogNumbers: ASTAI1281-07| 93-SRNP-1659| BOLD:AAB3286; **Taxon:** scientificName: Metaplagia robinsherwoodae; phylum: Arthropoda; class: Insecta; order: Diptera; family: Tachinidae; genus: Metaplagia; specificEpithet: robinsherwoodae; scientificNameAuthorship: Fleming & Wood, 2021; **Location:** continent: Central America; country: Costa Rica; countryCode: CR; stateProvince: Guanacaste; county: Sector Santa Rosa; locality: Area de Conservacion Guanacaste, Bosque San Emilio; verbatimElevation: 300; verbatimLatitude: 10.8439; verbatimLongitude: -85.6138; verbatimCoordinateSystem: Decimal degrees; **Identification:** identifiedBy: AJ Fleming; dateIdentified: 2021; **Event:** samplingProtocol: Reared from the larva of the Sphingidae, Manduca rustica; verbatimEventDate: 26-Jun-1993; **Record Level:** language: en; institutionCode: CNC; collectionCode: Insects; basisOfRecord: PreservedSpecimen
- **Type status:** Paratype. **Occurrence:** catalogNumber: DHJPAR0018635; recordedBy: D.H. Janzen, W. Hallwachs & gusaneros; individualCount: 1; lifeStage: adult; occurrenceStatus: present; preparations: pinned; otherCatalogNumbers: ASTAI1282-07| 92-SRNP-2162| BOLD:AAB3286; **Taxon:** scientificName: Metaplagia robinsherwoodae; phylum: Arthropoda; class: Insecta; order: Diptera; family: Tachinidae; genus: Metaplagia; specificEpithet: robinsherwoodae; scientificNameAuthorship: Fleming & Wood, 2021; **Location:** continent: Central America; country: Costa Rica; countryCode: CR; stateProvince: Guanacaste; county: Sector Santa Rosa; locality: Area de Conservacion Guanacaste, Cafetal; verbatimElevation: 280; verbatimLatitude: 10.8583; verbatimLongitude: -85.6109; verbatimCoordinateSystem: Decimal degrees; **Identification:** identifiedBy: AJ Fleming; dateIdentified: 2021; **Event:** samplingProtocol: Reared from the larva of the Sphingidae, Manduca rustica; verbatimEventDate: 03-Jul-1992; **Record Level:** language: en; institutionCode: CNC; collectionCode: Insects; basisOfRecord: PreservedSpecimen
- **Type status:** Paratype. **Occurrence:** catalogNumber: DHJPAR0018636; recordedBy: D.H. Janzen, W. Hallwachs & gusaneros; individualCount: 1; lifeStage: adult; occurrenceStatus: present; preparations: pinned; otherCatalogNumbers: ASTAI1283-07| 93-SRNP-1643| BOLD:AAB3286; **Taxon:** scientificName: Metaplagia robinsherwoodae; phylum: Arthropoda; class: Insecta; order: Diptera; family: Tachinidae; genus: Metaplagia; specificEpithet: robinsherwoodae; scientificNameAuthorship: Fleming & Wood, 2021; **Location:** continent: Central America; country: Costa Rica; countryCode: CR; stateProvince: Guanacaste; county: Sector Santa Rosa; locality: Area de Conservacion Guanacaste, Area Administrativa; verbatimElevation: 295; verbatimLatitude: 10.8376; verbatimLongitude: -85.6187; verbatimCoordinateSystem: Decimal degrees; **Identification:** identifiedBy: AJ Fleming; dateIdentified: 2021; **Event:** samplingProtocol: Reared from the larva of the Sphingidae, Manduca rustica; verbatimEventDate: 03-Jul-1993; **Record Level:** language: en; institutionCode: CNC; collectionCode: Insects; basisOfRecord: PreservedSpecimen
- **Type status:** Paratype. **Occurrence:** catalogNumber: DHJPAR0018637; recordedBy: D.H. Janzen, W. Hallwachs & gusaneros; individualCount: 1; lifeStage: adult; occurrenceStatus: present; preparations: pinned; otherCatalogNumbers: ASTAI1284-07| 91-SRNP-1170| BOLD:AAB3286; **Taxon:** scientificName: Metaplagia robinsherwoodae; phylum: Arthropoda; class: Insecta; order: Diptera; family: Tachinidae; genus: Metaplagia; specificEpithet: robinsherwoodae; scientificNameAuthorship: Fleming & Wood, 2021; **Location:** continent: Central America; country: Costa Rica; countryCode: CR; stateProvince: Guanacaste; county: Sector Santa Rosa; locality: Area de Conservacion Guanacaste, Bosque Humedo; verbatimElevation: 290; verbatimLatitude: 10.8514; verbatimLongitude: -85.608; verbatimCoordinateSystem: Decimal degrees; **Identification:** identifiedBy: AJ Fleming; dateIdentified: 2021; **Event:** samplingProtocol: Reared from the larva of the Sphingidae, Manduca rustica; verbatimEventDate: 28-Jul-1991; **Record Level:** language: en; institutionCode: CNC; collectionCode: Insects; basisOfRecord: PreservedSpecimen
- **Type status:** Paratype. **Occurrence:** catalogNumber: DHJPAR0018638; recordedBy: D.H. Janzen, W. Hallwachs & gusaneros; individualCount: 1; lifeStage: adult; occurrenceStatus: present; preparations: pinned; otherCatalogNumbers: ASTAI1285-07| 90-SRNP-647| BOLD:AAB3286; **Taxon:** scientificName: Metaplagia robinsherwoodae; phylum: Arthropoda; class: Insecta; order: Diptera; family: Tachinidae; genus: Metaplagia; specificEpithet: robinsherwoodae; scientificNameAuthorship: Fleming & Wood, 2021; **Location:** continent: Central America; country: Costa Rica; countryCode: CR; stateProvince: Guanacaste; county: Sector Santa Rosa; locality: Area de Conservacion Guanacaste, Area Administrativa; verbatimElevation: 295; verbatimLatitude: 10.8376; verbatimLongitude: -85.6187; verbatimCoordinateSystem: Decimal degrees; **Identification:** identifiedBy: AJ Fleming; dateIdentified: 2021; **Event:** samplingProtocol: Reared from the larva of the Sphingidae, Manduca rustica; verbatimEventDate: 04-Nov-1990; **Record Level:** language: en; institutionCode: CNC; collectionCode: Insects; basisOfRecord: PreservedSpecimen
- **Type status:** Paratype. **Occurrence:** catalogNumber: DHJPAR0018639; recordedBy: D.H. Janzen, W. Hallwachs & gusaneros; individualCount: 1; lifeStage: adult; occurrenceStatus: present; preparations: pinned; otherCatalogNumbers: ASTAI1286-07| 92-SRNP-2162| BOLD:AAB3286; **Taxon:** scientificName: Metaplagia robinsherwoodae; phylum: Arthropoda; class: Insecta; order: Diptera; family: Tachinidae; genus: Metaplagia; specificEpithet: robinsherwoodae; scientificNameAuthorship: Fleming & Wood, 2021; **Location:** continent: Central America; country: Costa Rica; countryCode: CR; stateProvince: Guanacaste; county: Sector Santa Rosa; locality: Area de Conservacion Guanacaste, Cafetal; verbatimElevation: 280; verbatimLatitude: 10.8583; verbatimLongitude: -85.6109; verbatimCoordinateSystem: Decimal degrees; **Identification:** identifiedBy: AJ Fleming; dateIdentified: 2021; **Event:** samplingProtocol: Reared from the larva of the Sphingidae, Manduca rustica; verbatimEventDate: 03-Jul-1992; **Record Level:** language: en; institutionCode: CNC; collectionCode: Insects; basisOfRecord: PreservedSpecimen
- **Type status:** Paratype. **Occurrence:** catalogNumber: DHJPAR0018628; recordedBy: D.H. Janzen, W. Hallwachs & Guillermo Pereira; individualCount: 1; lifeStage: adult; occurrenceStatus: present; preparations: pinned; otherCatalogNumbers: ASTAI1275-07| 98-SRNP-9161| BOLD:AAB3286; **Taxon:** scientificName: Metaplagia robinsherwoodae; phylum: Arthropoda; class: Insecta; order: Diptera; family: Tachinidae; genus: Metaplagia; specificEpithet: robinsherwoodae; scientificNameAuthorship: Fleming & Wood, 2021; **Location:** continent: Central America; country: Costa Rica; countryCode: CR; stateProvince: Guanacaste; county: Sector Santa Rosa; locality: Area de Conservacion Guanacaste, Area Administrativa; verbatimElevation: 295; verbatimLatitude: 10.8376; verbatimLongitude: -85.6187; verbatimCoordinateSystem: Decimal degrees; **Identification:** identifiedBy: AJ Fleming; dateIdentified: 2021; **Event:** samplingProtocol: Reared from the larva of the Sphingidae, Manduca rustica; verbatimEventDate: 31-Oct-1998; **Record Level:** language: en; institutionCode: CNC; collectionCode: Insects; basisOfRecord: PreservedSpecimen
- **Type status:** Paratype. **Occurrence:** catalogNumber: DHJPAR0018713; recordedBy: D.H. Janzen, W. Hallwachs & Daniel H. Janzen; individualCount: 1; lifeStage: adult; occurrenceStatus: present; preparations: pinned; otherCatalogNumbers: ASTAI1360-07| 98-SRNP-8704| BOLD:AAB3286; **Taxon:** scientificName: Metaplagia robinsherwoodae; phylum: Arthropoda; class: Insecta; order: Diptera; family: Tachinidae; genus: Metaplagia; specificEpithet: robinsherwoodae; scientificNameAuthorship: Fleming & Wood, 2021; **Location:** continent: Central America; country: Costa Rica; countryCode: CR; stateProvince: Guanacaste; county: Sector Santa Rosa; locality: Area de Conservacion Guanacaste, Area Administrativa; verbatimElevation: 295; verbatimLatitude: 10.8376; verbatimLongitude: -85.6187; verbatimCoordinateSystem: Decimal degrees; **Identification:** identifiedBy: AJ Fleming; dateIdentified: 2021; **Event:** samplingProtocol: Reared from the larva of the Sphingidae, Manduca rustica; verbatimEventDate: 07-May-1999; **Record Level:** language: en; institutionCode: CNC; collectionCode: Insects; basisOfRecord: PreservedSpecimen
- **Type status:** Paratype. **Occurrence:** catalogNumber: DHJPAR0018714; recordedBy: D.H. Janzen, W. Hallwachs & Guillermo Pereira; individualCount: 1; lifeStage: adult; occurrenceStatus: present; preparations: pinned; otherCatalogNumbers: ASTAI1361-07| 98-SRNP-8687| BOLD:AAB3286; **Taxon:** scientificName: Metaplagia robinsherwoodae; phylum: Arthropoda; class: Insecta; order: Diptera; family: Tachinidae; genus: Metaplagia; specificEpithet: robinsherwoodae; scientificNameAuthorship: Fleming & Wood, 2021; **Location:** continent: Central America; country: Costa Rica; countryCode: CR; stateProvince: Guanacaste; county: Sector Santa Rosa; locality: Area de Conservacion Guanacaste, Quebrada Cebollines; verbatimElevation: 270; verbatimLatitude: 10.8222; verbatimLongitude: -85.6434; verbatimCoordinateSystem: Decimal degrees; **Identification:** identifiedBy: AJ Fleming; dateIdentified: 2021; **Event:** samplingProtocol: Reared from the larva of the Sphingidae, Manduca rustica; verbatimEventDate: 08-May-1999; **Record Level:** language: en; institutionCode: CNC; collectionCode: Insects; basisOfRecord: PreservedSpecimen
- **Type status:** Paratype. **Occurrence:** catalogNumber: DHJPAR0018715; recordedBy: D.H. Janzen, W. Hallwachs & Elieth Cantillano; individualCount: 1; lifeStage: adult; occurrenceStatus: present; preparations: pinned; otherCatalogNumbers: ASTAI1362-07| 98-SRNP-8707| BOLD:AAB3286; **Taxon:** scientificName: Metaplagia robinsherwoodae; phylum: Arthropoda; class: Insecta; order: Diptera; family: Tachinidae; genus: Metaplagia; specificEpithet: robinsherwoodae; scientificNameAuthorship: Fleming & Wood, 2021; **Location:** continent: Central America; country: Costa Rica; countryCode: CR; stateProvince: Guanacaste; county: Sector Santa Rosa; locality: Area de Conservacion Guanacaste, Bosque San Emilio; verbatimElevation: 300; verbatimLatitude: 10.8439; verbatimLongitude: -85.6138; verbatimCoordinateSystem: Decimal degrees; **Identification:** identifiedBy: AJ Fleming; dateIdentified: 2021; **Event:** samplingProtocol: Reared from the larva of the Sphingidae, Manduca rustica; verbatimEventDate: 08-May-1999; **Record Level:** language: en; institutionCode: CNC; collectionCode: Insects; basisOfRecord: PreservedSpecimen
- **Type status:** Paratype. **Occurrence:** catalogNumber: DHJPAR0018716; recordedBy: D.H. Janzen, W. Hallwachs & Ruth Franco; individualCount: 1; lifeStage: adult; occurrenceStatus: present; preparations: pinned; otherCatalogNumbers: ASTAI1363-07| 98-SRNP-8815| BOLD:AAB3286; **Taxon:** scientificName: Metaplagia robinsherwoodae; phylum: Arthropoda; class: Insecta; order: Diptera; family: Tachinidae; genus: Metaplagia; specificEpithet: robinsherwoodae; scientificNameAuthorship: Fleming & Wood, 2021; **Location:** continent: Central America; country: Costa Rica; countryCode: CR; stateProvince: Guanacaste; county: Sector Santa Rosa; locality: Area de Conservacion Guanacaste, Cafetal; verbatimElevation: 280; verbatimLatitude: 10.8583; verbatimLongitude: -85.6109; verbatimCoordinateSystem: Decimal degrees; **Identification:** identifiedBy: AJ Fleming; dateIdentified: 2021; **Event:** samplingProtocol: Reared from the larva of the Sphingidae, Manduca rustica; verbatimEventDate: 08-May-1999; **Record Level:** language: en; institutionCode: CNC; collectionCode: Insects; basisOfRecord: PreservedSpecimen
- **Type status:** Paratype. **Occurrence:** catalogNumber: DHJPAR0018717; recordedBy: D.H. Janzen, W. Hallwachs & Manuel Pereira; individualCount: 1; lifeStage: adult; occurrenceStatus: present; preparations: pinned; otherCatalogNumbers: ASTAI1364-07| 98-SRNP-8789| BOLD:AAB3286; **Taxon:** scientificName: Metaplagia robinsherwoodae; phylum: Arthropoda; class: Insecta; order: Diptera; family: Tachinidae; genus: Metaplagia; specificEpithet: robinsherwoodae; scientificNameAuthorship: Fleming & Wood, 2021; **Location:** continent: Central America; country: Costa Rica; countryCode: CR; stateProvince: Guanacaste; county: Sector Santa Rosa; locality: Area de Conservacion Guanacaste, Chiringon; verbatimElevation: 250; verbatimLatitude: 10.8388; verbatimLongitude: -85.601; verbatimCoordinateSystem: Decimal degrees; **Identification:** identifiedBy: AJ Fleming; dateIdentified: 2021; **Event:** samplingProtocol: Reared from the larva of the Sphingidae, Manduca rustica; verbatimEventDate: 08-May-1999; **Record Level:** language: en; institutionCode: CNC; collectionCode: Insects; basisOfRecord: PreservedSpecimen
- **Type status:** Paratype. **Occurrence:** catalogNumber: DHJPAR0018718; recordedBy: D.H. Janzen, W. Hallwachs & Adrian Guadamuz; individualCount: 1; lifeStage: adult; occurrenceStatus: present; preparations: pinned; otherCatalogNumbers: ASTAI1365-07| 98-SRNP-9068| BOLD:AAB3286; **Taxon:** scientificName: Metaplagia robinsherwoodae; phylum: Arthropoda; class: Insecta; order: Diptera; family: Tachinidae; genus: Metaplagia; specificEpithet: robinsherwoodae; scientificNameAuthorship: Fleming & Wood, 2021; **Location:** continent: Central America; country: Costa Rica; countryCode: CR; stateProvince: Guanacaste; county: Sector Santa Rosa; locality: Area de Conservacion Guanacaste, Cafetal; verbatimElevation: 280; verbatimLatitude: 10.8583; verbatimLongitude: -85.6109; verbatimCoordinateSystem: Decimal degrees; **Identification:** identifiedBy: AJ Fleming; dateIdentified: 2021; **Event:** samplingProtocol: Reared from the larva of the Sphingidae, Manduca rustica; verbatimEventDate: 07-May-1999; **Record Level:** language: en; institutionCode: CNC; collectionCode: Insects; basisOfRecord: PreservedSpecimen
- **Type status:** Paratype. **Occurrence:** catalogNumber: DHJPAR0018719; recordedBy: D.H. Janzen, W. Hallwachs & Guillermo Pereira; individualCount: 1; lifeStage: adult; occurrenceStatus: present; preparations: pinned; otherCatalogNumbers: ASTAI1366-07| 98-SRNP-8893| BOLD:AAB3286; **Taxon:** scientificName: Metaplagia robinsherwoodae; phylum: Arthropoda; class: Insecta; order: Diptera; family: Tachinidae; genus: Metaplagia; specificEpithet: robinsherwoodae; scientificNameAuthorship: Fleming & Wood, 2021; **Location:** continent: Central America; country: Costa Rica; countryCode: CR; stateProvince: Guanacaste; county: Sector Santa Rosa; locality: Area de Conservacion Guanacaste, Laguna Escondida; verbatimElevation: 285; verbatimLatitude: 10.8487; verbatimLongitude: -85.6305; verbatimCoordinateSystem: Decimal degrees; **Identification:** identifiedBy: AJ Fleming; dateIdentified: 2021; **Event:** samplingProtocol: Reared from the larva of the Sphingidae, Manduca rustica; verbatimEventDate: 08-May-1999; **Record Level:** language: en; institutionCode: CNC; collectionCode: Insects; basisOfRecord: PreservedSpecimen
- **Type status:** Paratype. **Occurrence:** catalogNumber: DHJPAR0018720; recordedBy: D.H. Janzen, W. Hallwachs & Ruth Franco; individualCount: 1; lifeStage: adult; occurrenceStatus: present; preparations: pinned; otherCatalogNumbers: ASTAI1367-07| 98-SRNP-8635| BOLD:AAB3286; **Taxon:** scientificName: Metaplagia robinsherwoodae; phylum: Arthropoda; class: Insecta; order: Diptera; family: Tachinidae; genus: Metaplagia; specificEpithet: robinsherwoodae; scientificNameAuthorship: Fleming & Wood, 2021; **Location:** continent: Central America; country: Costa Rica; countryCode: CR; stateProvince: Guanacaste; county: Sector Santa Rosa; locality: Area de Conservacion Guanacaste, Quebrada Cebollines; verbatimElevation: 270; verbatimLatitude: 10.8222; verbatimLongitude: -85.6434; verbatimCoordinateSystem: Decimal degrees; **Identification:** identifiedBy: AJ Fleming; dateIdentified: 2021; **Event:** samplingProtocol: Reared from the larva of the Sphingidae, Manduca rustica; verbatimEventDate: 24-Jul-1998; **Record Level:** language: en; institutionCode: CNC; collectionCode: Insects; basisOfRecord: PreservedSpecimen
- **Type status:** Paratype. **Occurrence:** catalogNumber: DHJPAR0018725; recordedBy: D.H. Janzen, W. Hallwachs & Guillermo Pereira; individualCount: 1; sex: Male; lifeStage: adult; occurrenceStatus: present; preparations: pinned; otherCatalogNumbers: ASTAI1372-07| 98-SRNP-9161| BOLD:AAB3286; **Taxon:** scientificName: Metaplagia robinsherwoodae; phylum: Arthropoda; class: Insecta; order: Diptera; family: Tachinidae; genus: Metaplagia; specificEpithet: robinsherwoodae; scientificNameAuthorship: Fleming & Wood, 2021; **Location:** continent: Central America; country: Costa Rica; countryCode: CR; stateProvince: Guanacaste; county: Sector Santa Rosa; locality: Area de Conservacion Guanacaste, Area Administrativa; verbatimElevation: 295; verbatimLatitude: 10.8376; verbatimLongitude: -85.6187; verbatimCoordinateSystem: Decimal degrees; **Identification:** identifiedBy: AJ Fleming; dateIdentified: 2021; **Event:** samplingProtocol: Reared from the larva of the Sphingidae, Manduca rustica; verbatimEventDate: 31-Oct-1998; **Record Level:** language: en; institutionCode: CNC; collectionCode: Insects; basisOfRecord: PreservedSpecimen
- **Type status:** Paratype. **Occurrence:** catalogNumber: DHJPAR0018726; recordedBy: D.H. Janzen, W. Hallwachs & Manuel Pereira; individualCount: 1; lifeStage: adult; occurrenceStatus: present; preparations: pinned; otherCatalogNumbers: ASTAI1373-07| 98-SRNP-9080| BOLD:AAB3286; **Taxon:** scientificName: Metaplagia robinsherwoodae; phylum: Arthropoda; class: Insecta; order: Diptera; family: Tachinidae; genus: Metaplagia; specificEpithet: robinsherwoodae; scientificNameAuthorship: Fleming & Wood, 2021; **Location:** continent: Central America; country: Costa Rica; countryCode: CR; stateProvince: Guanacaste; county: Sector Santa Rosa; locality: Area de Conservacion Guanacaste, Cafetal; verbatimElevation: 280; verbatimLatitude: 10.8583; verbatimLongitude: -85.6109; verbatimCoordinateSystem: Decimal degrees; **Identification:** identifiedBy: AJ Fleming; dateIdentified: 2021; **Event:** samplingProtocol: Reared from the larva of the Sphingidae, Manduca rustica; verbatimEventDate: 28-Mar-1999; **Record Level:** language: en; institutionCode: CNC; collectionCode: Insects; basisOfRecord: PreservedSpecimen
- **Type status:** Paratype. **Occurrence:** catalogNumber: DHJPAR0018728; recordedBy: D.H. Janzen, W. Hallwachs & Guillermo Pereira; individualCount: 1; lifeStage: adult; occurrenceStatus: present; preparations: pinned; otherCatalogNumbers: ASTAI1375-07| 98-SRNP-8715| BOLD:AAB3286; **Taxon:** scientificName: Metaplagia robinsherwoodae; phylum: Arthropoda; class: Insecta; order: Diptera; family: Tachinidae; genus: Metaplagia; specificEpithet: robinsherwoodae; scientificNameAuthorship: Fleming & Wood, 2021; **Location:** continent: Central America; country: Costa Rica; countryCode: CR; stateProvince: Guanacaste; county: Sector Santa Rosa; locality: Area de Conservacion Guanacaste, Bosque San Emilio; verbatimElevation: 300; verbatimLatitude: 10.8439; verbatimLongitude: -85.6138; verbatimCoordinateSystem: Decimal degrees; **Identification:** identifiedBy: AJ Fleming; dateIdentified: 2021; **Event:** samplingProtocol: Reared from the larva of the Sphingidae, Manduca rustica; verbatimEventDate: 22-Jul-1998; **Record Level:** language: en; institutionCode: CNC; collectionCode: Insects; basisOfRecord: PreservedSpecimen
- **Type status:** Paratype. **Occurrence:** catalogNumber: DHJPAR0018729; recordedBy: D.H. Janzen, W. Hallwachs & Manuel Pereira; individualCount: 1; lifeStage: adult; occurrenceStatus: present; preparations: pinned; otherCatalogNumbers: ASTAI1376-07| 98-SRNP-8789| BOLD:AAB3286; **Taxon:** scientificName: Metaplagia robinsherwoodae; phylum: Arthropoda; class: Insecta; order: Diptera; family: Tachinidae; genus: Metaplagia; specificEpithet: robinsherwoodae; scientificNameAuthorship: Fleming & Wood, 2021; **Location:** continent: Central America; country: Costa Rica; countryCode: CR; stateProvince: Guanacaste; county: Sector Santa Rosa; locality: Area de Conservacion Guanacaste, Chiringon; verbatimElevation: 250; verbatimLatitude: 10.8388; verbatimLongitude: -85.601; verbatimCoordinateSystem: Decimal degrees; **Identification:** identifiedBy: AJ Fleming; dateIdentified: 2021; **Event:** samplingProtocol: Reared from the larva of the Sphingidae, Manduca rustica; verbatimEventDate: 08-May-1999; **Record Level:** language: en; institutionCode: CNC; collectionCode: Insects; basisOfRecord: PreservedSpecimen
- **Type status:** Paratype. **Occurrence:** catalogNumber: DHJPAR0018730; recordedBy: D.H. Janzen, W. Hallwachs & Elieth Cantillano; individualCount: 1; lifeStage: adult; occurrenceStatus: present; preparations: pinned; otherCatalogNumbers: ASTAI1377-07| 98-SRNP-8707| BOLD:AAB3286; **Taxon:** scientificName: Metaplagia robinsherwoodae; phylum: Arthropoda; class: Insecta; order: Diptera; family: Tachinidae; genus: Metaplagia; specificEpithet: robinsherwoodae; scientificNameAuthorship: Fleming & Wood, 2021; **Location:** continent: Central America; country: Costa Rica; countryCode: CR; stateProvince: Guanacaste; county: Sector Santa Rosa; locality: Area de Conservacion Guanacaste, Bosque San Emilio; verbatimElevation: 300; verbatimLatitude: 10.8439; verbatimLongitude: -85.6138; verbatimCoordinateSystem: Decimal degrees; **Identification:** identifiedBy: AJ Fleming; dateIdentified: 2021; **Event:** samplingProtocol: Reared from the larva of the Sphingidae, Manduca rustica; verbatimEventDate: 08-May-1999; **Record Level:** language: en; institutionCode: CNC; collectionCode: Insects; basisOfRecord: PreservedSpecimen
- **Type status:** Paratype. **Occurrence:** catalogNumber: DHJPAR0018731; recordedBy: D.H. Janzen, W. Hallwachs & Ruth Franco; individualCount: 1; lifeStage: adult; occurrenceStatus: present; preparations: pinned; otherCatalogNumbers: ASTAI1378-07| 98-SRNP-8815| BOLD:AAB3286; **Taxon:** scientificName: Metaplagia robinsherwoodae; phylum: Arthropoda; class: Insecta; order: Diptera; family: Tachinidae; genus: Metaplagia; specificEpithet: robinsherwoodae; scientificNameAuthorship: Fleming & Wood, 2021; **Location:** continent: Central America; country: Costa Rica; countryCode: CR; stateProvince: Guanacaste; county: Sector Santa Rosa; locality: Area de Conservacion Guanacaste, Cafetal; verbatimElevation: 280; verbatimLatitude: 10.8583; verbatimLongitude: -85.6109; verbatimCoordinateSystem: Decimal degrees; **Identification:** identifiedBy: AJ Fleming; dateIdentified: 2021; **Event:** samplingProtocol: Reared from the larva of the Sphingidae, Manduca rustica; verbatimEventDate: 15-Apr-1999; **Record Level:** language: en; institutionCode: CNC; collectionCode: Insects; basisOfRecord: PreservedSpecimen
- **Type status:** Paratype. **Occurrence:** catalogNumber: DHJPAR0060291; recordedBy: D.H. Janzen, W. Hallwachs & Tanner Frank; individualCount: 1; lifeStage: adult; occurrenceStatus: present; preparations: pinned; otherCatalogNumbers: ACGBA6712-17| 16-SRNP-10177| BOLD:AAB3286; **Taxon:** scientificName: Metaplagia robinsherwoodae; phylum: Arthropoda; class: Insecta; order: Diptera; family: Tachinidae; genus: Metaplagia; specificEpithet: robinsherwoodae; scientificNameAuthorship: Fleming & Wood, 2021; **Location:** continent: Central America; country: Costa Rica; countryCode: CR; stateProvince: Guanacaste; county: Sector Santa Rosa; locality: Area de Conservacion Guanacaste, Area Administrativa; verbatimElevation: 295; verbatimLatitude: 10.8376; verbatimLongitude: -85.6187; verbatimCoordinateSystem: Decimal degrees; **Identification:** identifiedBy: AJ Fleming; dateIdentified: 2021; **Event:** samplingProtocol: Reared from the larva of the Sphingidae, Manduca rustica; **Record Level:** language: en; institutionCode: CNC; collectionCode: Insects; basisOfRecord: PreservedSpecimen
- **Type status:** Paratype. **Occurrence:** catalogNumber: DHJPAR0062861; recordedBy: D.H. Janzen, W. Hallwachs & Erasmo Coronado; individualCount: 1; sex: Female; lifeStage: adult; occurrenceStatus: present; preparations: pinned; otherCatalogNumbers: ACGBA9195-18| 17-SRNP-10052| BOLD:AAB3286; **Taxon:** scientificName: Metaplagia robinsherwoodae; phylum: Arthropoda; class: Insecta; order: Diptera; family: Tachinidae; genus: Metaplagia; specificEpithet: robinsherwoodae; scientificNameAuthorship: Fleming & Wood, 2021; **Location:** continent: Central America; country: Costa Rica; countryCode: CR; stateProvince: Guanacaste; county: Sector Santa Rosa; locality: Area de Conservacion Guanacaste, Area Administrativa; verbatimElevation: 295; verbatimLatitude: 10.8376; verbatimLongitude: -85.6187; verbatimCoordinateSystem: Decimal degrees; **Identification:** identifiedBy: AJ Fleming; dateIdentified: 2021; **Event:** samplingProtocol: Reared from the larva of the Sphingidae, Manduca rustica; verbatimEventDate: 09-Jun-2018; **Record Level:** language: en; institutionCode: CNC; collectionCode: Insects; basisOfRecord: PreservedSpecimen

#### Description

Male (Fig. 9), **head** (Fig. 9c, d): fronto-orbital plate wide, coloration silver with gold tomentum over uppermost 50%, appearing as pale brassy gold along upper 25% of occipital margin of eye; frontal vitta wide, 1/5 width of fronto-orbital plate almost entirely covered in gold tomentum; ocellar setae strongly proclinate slightly divergent; with 3 proclinate orbital setae (middle proclinate orbital seta most often almost hair-like reduced) and 1 reclinate orbital seta fronto-orbital plate with only a sparse few short black setulae interspersed amongst frontal setae; sparse fronto-orbital setulae extending below lower margin of the pedicel (very few), these not intermingled with those in angle of parafacial; parafacial wholly silver tomentose with a single row of parafacial setulae mid-way along parafacial near facial ridge; palps slightly spathulate at tips almost oar-shaped, apically acutely inwardly curved, setulose along outer margins, inner margin appearing bare; arista dark brown-black. **Thorax** (Fig. 9a, b): black ground color with pale grey with a slightly brassy tinged tomentum, laterally this color transitions to a darkened grey tomentum; 4–5 postpronotal setae; supra-alar setae 2:3; intra-alar setae 3:3; dorsocentral setae 3:3-4; acrostichal setae 3:3; katepisternum with 3 setae. Infrasquamal setae absent. Scutellum black ground color, with tomentum concolorous with disc of thorax; with four pairs marginal setae; apical scutellar setae absent; basal scutellar 2/3 length of subapical setae; subapical setae convergent, lateral scutellar setae strongly divergent. Legs: black ground color; mid tibia with 2–3 strong anteroventral setae; tarsal claws and pulvilli shorter than last tarsomere. Wings: R_1_ bare, R_4+5_ setulose from node slightly beyond crossvein r-m. **Abdomen** (Fig. 9a, b): ground color black; ST1+2 glabrous black, pale silver tomentum occupying anterior 60% of T3–T5; tomentum extending to ventral surface of abdomen on T4 and T5; median marginal setae present on T3 and complete rows on T4 and T5.

**Terminalia** (Fig. 10): posterior margin of sternite 5 with a deeply excavated and sculptured U-shaped median cleft (Fig. 10d); lateral lobes of sternite apically rounded, widened at mid-point and tapering again towards base, with a vestiture of short setae along disc, 3x as long along caudal margin; basal section slightly less than 1/5 the length of apical lobes. Cerci, in posterior view, medially separated, with a few short setae along entire length, setae lengthening posteriorly towards anal operculum (Fig. 10a). In lateral view, bowed and sharply tapered apically (Fig. 10b, c). Surstylus well-developed, stout basally in lateral view, like a broadly rounded triangle, appearing like a rounded ellipsoid leaf shape; in posterior view, basally enlarged and apically straight.

Female (Fig. 11) as in male, except in the following aspects: **head** (Fig. 11c, d): fronto-orbital just under 1/2 of head width, vitta 1/4 width of fronto-orbital plate; ocellar setae lateraloclinate; post-pedicel slightly orange along basal 1/5. Legs (Fig. 11b): mid-tibia with 3–4 irregularly-sized strong anteroventral setae.

#### Diagnosis

*Metaplagia robinsherwoodae* **sp. n.** can be distinguished from all other *Metaplagia* by the following combination of traits: fronto-orbital plate at least 75% gold, with a silver parafacial and setulae on vein R_4+5_ extending well beyond crossvein r-m. *Metaplagia robinsherwoodae* **sp. n.** can be separated from *M. leahdennisae and M. paulinesaribasae* by the presence of setulae on R_4+5_ extending well beyond crossvein r-m and it is separated from *M. facialis* and *M. cordata* by the presence of gold on its fronto-orbital plate. *Metaplagia robinsherwoodae* **sp. n.** is clearly distinguished by its COI sequence clustered within the Barcode Identification Number (BIN) BOLD:AAB3286.

#### Etymology

*Metaplagia robinsherwoodae* **sp. n.** is named in honor of Robin Sherwood for her many years of coordinating and administrating a plethora of problems and events in the Academic Office of the Department of Biology, University of Pennsylvania, Philadelphia, Pennsylvania.

#### Distribution

Costa Rica, ACG, Guanacaste Province, 250–300 m elevation.

#### Ecology

*Metaplagia robinsherwoodae* **sp. n.** has been reared 28 times from one species of Lepidoptera in the family Sphingidae: *Manduca rustica* (Fabricius, 1775), in dry forest.

### Metaplagia svetlanakozikae

Fleming & Wood sp. n.

D1AF1488-89B6-54D6-BE31-48C1E43F3C37

42C752D8-B95A-4450-B7D2-0AEB10008472

#### Materials

- **Type status:** Holotype. **Occurrence:** catalogNumber: DHJPAR0018732; recordedBy: D.H. Janzen, W. Hallwachs & Guillermo Pereira; individualCount: 1; sex: Male; lifeStage: adult; occurrenceStatus: present; preparations: pinned; otherCatalogNumbers: ASTAI1379-07| 01-SRNP-13896| BOLD:AAD5456; **Taxon:** scientificName: Metaplagia svetlanakozikae; phylum: Arthropoda; class: Insecta; order: Diptera; family: Tachinidae; genus: Metaplagia; specificEpithet: svetlanakozikae; scientificNameAuthorship: Fleming & Wood, 2021; **Location:** continent: Central America; country: Costa Rica; countryCode: CR; stateProvince: Guanacaste; county: Sector Santa Rosa; locality: Area de Conservacion Guanacaste, Vado Rio Calera; verbatimElevation: 10; verbatimLatitude: 10.8027; verbatimLongitude: -85.6742; verbatimCoordinateSystem: Decimal degrees; **Identification:** identifiedBy: AJ Fleming; dateIdentified: 2021; **Event:** samplingProtocol: Reared from the larva of the Sphingidae, Agrius cingulata; verbatimEventDate: 12-Nov-2001; **Record Level:** language: en; institutionCode: CNC; collectionCode: Insects; basisOfRecord: PreservedSpecimen
- **Type status:** Paratype. **Occurrence:** catalogNumber: DHJPAR0018721; recordedBy: D.H. Janzen, W. Hallwachs & Manuel Pereira; individualCount: 1; lifeStage: adult; occurrenceStatus: present; preparations: pinned; otherCatalogNumbers: ASTAI1368-07| 98-SRNP-8103| BOLD:AAD5456; **Taxon:** scientificName: Metaplagia svetlanakozikae; phylum: Arthropoda; class: Insecta; order: Diptera; family: Tachinidae; genus: Metaplagia; specificEpithet: svetlanakozikae; scientificNameAuthorship: Fleming & Wood, 2021; **Location:** continent: Central America; country: Costa Rica; countryCode: CR; stateProvince: Guanacaste; county: Sector Santa Rosa; locality: Area de Conservacion Guanacaste, Quebrada Costa Rica; verbatimElevation: 275; verbatimLatitude: 10.8274; verbatimLongitude: -85.6365; verbatimCoordinateSystem: Decimal degrees; **Identification:** identifiedBy: AJ Fleming; dateIdentified: 2021; **Event:** samplingProtocol: Reared from the larva of the Sphingidae, Agrius cingulata; verbatimEventDate: 06-May-1999; **Record Level:** language: en; institutionCode: CNC; collectionCode: Insects; basisOfRecord: PreservedSpecimen
- **Type status:** Paratype. **Occurrence:** catalogNumber: DHJPAR0018722; recordedBy: D.H. Janzen, W. Hallwachs & Guillermo Pereira; individualCount: 1; sex: Female; lifeStage: adult; occurrenceStatus: present; preparations: pinned; otherCatalogNumbers: ASTAI1369-07| 98-SRNP-8104| BOLD:AAD5456; **Taxon:** scientificName: Metaplagia svetlanakozikae; phylum: Arthropoda; class: Insecta; order: Diptera; family: Tachinidae; genus: Metaplagia; specificEpithet: svetlanakozikae; scientificNameAuthorship: Fleming & Wood, 2021; **Location:** continent: Central America; country: Costa Rica; countryCode: CR; stateProvince: Guanacaste; county: Sector Santa Rosa; locality: Area de Conservacion Guanacaste, Quebrada Costa Rica; verbatimElevation: 275; verbatimLatitude: 10.8274; verbatimLongitude: -85.6365; verbatimCoordinateSystem: Decimal degrees; **Identification:** identifiedBy: AJ Fleming; dateIdentified: 2021; **Event:** samplingProtocol: Reared from the larva of the Sphingidae, Agrius cingulata; verbatimEventDate: 08-Jun-1998; **Record Level:** language: en; institutionCode: CNC; collectionCode: Insects; basisOfRecord: PreservedSpecimen
- **Type status:** Paratype. **Occurrence:** catalogNumber: DHJPAR0018723; recordedBy: D.H. Janzen, W. Hallwachs & Manuel Pereira; individualCount: 1; lifeStage: adult; occurrenceStatus: present; preparations: pinned; otherCatalogNumbers: ASTAI1370-07| 98-SRNP-8248| BOLD:AAD5456; **Taxon:** scientificName: Metaplagia svetlanakozikae; phylum: Arthropoda; class: Insecta; order: Diptera; family: Tachinidae; genus: Metaplagia; specificEpithet: svetlanakozikae; scientificNameAuthorship: Fleming & Wood, 2021; **Location:** continent: Central America; country: Costa Rica; countryCode: CR; stateProvince: Guanacaste; county: Sector Santa Rosa; locality: Area de Conservacion Guanacaste, Quebrada Costa Rica; verbatimElevation: 275; verbatimLatitude: 10.8274; verbatimLongitude: -85.6365; verbatimCoordinateSystem: Decimal degrees; **Identification:** identifiedBy: AJ Fleming; dateIdentified: 2021; **Event:** samplingProtocol: Reared from the larva of the Sphingidae, Agrius cingulata; verbatimEventDate: 10-Jul-1998; **Record Level:** language: en; institutionCode: CNC; collectionCode: Insects; basisOfRecord: PreservedSpecimen
- **Type status:** Paratype. **Occurrence:** catalogNumber: DHJPAR0018630; recordedBy: D.H. Janzen, W. Hallwachs & Guillermo Pereira; individualCount: 1; lifeStage: adult; occurrenceStatus: present; preparations: pinned; otherCatalogNumbers: ASTAI1277-07| 98-SRNP-8104| BOLD:AAE2876; **Taxon:** scientificName: Metaplagia svetlanakozikae; phylum: Arthropoda; class: Insecta; order: Diptera; family: Tachinidae; genus: Metaplagia; specificEpithet: svetlanakozikae; scientificNameAuthorship: Fleming & Wood, 2021; **Location:** continent: Central America; country: Costa Rica; countryCode: CR; stateProvince: Guanacaste; county: Sector Santa Rosa; locality: Area de Conservacion Guanacaste, Quebrada Costa Rica; verbatimElevation: 275; verbatimLatitude: 10.8274; verbatimLongitude: -85.6365; verbatimCoordinateSystem: Decimal degrees; **Identification:** identifiedBy: AJ Fleming; dateIdentified: 2021; **Event:** samplingProtocol: Reared from the larva of the Sphingidae, Agrius cingulata; verbatimEventDate: 08-Jun-1998; **Record Level:** language: en; institutionCode: CNC; collectionCode: Insects; basisOfRecord: PreservedSpecimen
- **Type status:** Paratype. **Occurrence:** catalogNumber: DHJPAR0018733; recordedBy: D.H. Janzen, W. Hallwachs & gusaneros; individualCount: 1; sex: Male; lifeStage: adult; occurrenceStatus: present; preparations: pinned; otherCatalogNumbers: ASTAI1380-07| 01-SRNP-14594| BOLD:AAD5456; **Taxon:** scientificName: Metaplagia svetlanakozikae; phylum: Arthropoda; class: Insecta; order: Diptera; family: Tachinidae; genus: Metaplagia; specificEpithet: svetlanakozikae; scientificNameAuthorship: Fleming & Wood, 2021; **Location:** continent: Central America; country: Costa Rica; countryCode: CR; stateProvince: Guanacaste; county: Sector Santa Rosa; locality: Area de Conservacion Guanacaste, Cafetal; verbatimElevation: 280; verbatimLatitude: 10.8583; verbatimLongitude: -85.6109; verbatimCoordinateSystem: Decimal degrees; **Identification:** identifiedBy: AJ Fleming; dateIdentified: 2021; **Event:** samplingProtocol: Reared from the larva of the Sphingidae, Agrius cingulata; verbatimEventDate: 29-May-2002; **Record Level:** language: en; institutionCode: CNC; collectionCode: Insects; basisOfRecord: PreservedSpecimen
- **Type status:** Paratype. **Occurrence:** catalogNumber: DHJPAR0018734; recordedBy: D.H. Janzen, W. Hallwachs & Guillermo Pereira; individualCount: 1; lifeStage: adult; occurrenceStatus: present; preparations: pinned; otherCatalogNumbers: ASTAI1381-07| 01-SRNP-13896| BOLD:AAD5456; **Taxon:** scientificName: Metaplagia svetlanakozikae; phylum: Arthropoda; class: Insecta; order: Diptera; family: Tachinidae; genus: Metaplagia; specificEpithet: svetlanakozikae; scientificNameAuthorship: Fleming & Wood, 2021; **Location:** continent: Central America; country: Costa Rica; countryCode: CR; stateProvince: Guanacaste; county: Sector Santa Rosa; locality: Area de Conservacion Guanacaste, Vado Rio Calera; verbatimElevation: 10; verbatimLatitude: 10.8027; verbatimLongitude: -85.6742; verbatimCoordinateSystem: Decimal degrees; **Identification:** identifiedBy: AJ Fleming; dateIdentified: 2021; **Event:** samplingProtocol: Reared from the larva of the Sphingidae, Agrius cingulata; verbatimEventDate: 12-Nov-2001; **Record Level:** language: en; institutionCode: CNC; collectionCode: Insects; basisOfRecord: PreservedSpecimen
- **Type status:** Paratype. **Occurrence:** catalogNumber: DHJPAR0018735; recordedBy: D.H. Janzen, W. Hallwachs & Guillermo Pereira; individualCount: 1; lifeStage: adult; occurrenceStatus: present; preparations: pinned; otherCatalogNumbers: ASTAI1382-07| 01-SRNP-13896| BOLD:AAD5456; **Taxon:** scientificName: Metaplagia svetlanakozikae; phylum: Arthropoda; class: Insecta; order: Diptera; family: Tachinidae; genus: Metaplagia; specificEpithet: svetlanakozikae; scientificNameAuthorship: Fleming & Wood, 2021; **Location:** continent: Central America; country: Costa Rica; countryCode: CR; stateProvince: Guanacaste; county: Sector Santa Rosa; locality: Area de Conservacion Guanacaste, Vado Rio Calera; verbatimElevation: 10; verbatimLatitude: 10.8027; verbatimLongitude: -85.6742; verbatimCoordinateSystem: Decimal degrees; **Identification:** identifiedBy: AJ Fleming; dateIdentified: 2021; **Event:** samplingProtocol: Reared from the larva of the Sphingidae, Agrius cingulata; verbatimEventDate: 12-Nov-2001; **Record Level:** language: en; institutionCode: CNC; collectionCode: Insects; basisOfRecord: PreservedSpecimen

#### Description

Male (Fig. 12), **head** (Fig. 12c, d): fronto-orbital plate wide, coloration silver with pale brassy tomentum over uppermost 10%, appearing as pale brassy gold along upper 25% of occipital margin of eye; vitta wide, 1/3 width of fronto-orbital plate; ocellar setae lateraloclinate with a slight proclinate skew; with 3 proclinate orbital setae (middle proclinate orbital seta shorter and thinner than the other two) and 1 reclinate (often outwardly lateraloclinate) orbital seta; fronto-orbital plate with a sparse vestiture of short black setulae interspersed amongst frontal setae; fronto-orbital setulae not extending below pedicel; parafacial wholly silver tomentose, upper 1/3 of parafacial with small grouping of short black setulae in line with frontal setae, but separated from fronto-orbital plate; palps very slightly spathulate at tips, apically acutely inwardly curved, sparsely setulose along ventral surface, inner and upper surfaces appearing bare; arista dark brown-black. **Thorax** (Fig. 12a, b): black ground color with pale grey tomentum, brassy tones can occur under certain light; 4 postpronotal setae, anteriormost seta thin and weak almost hair-like; supra-alar setae 2:3; intra-alar setae 3:3; dorsocentral setae 3:3; acrostichal setae 3:3; katepisternum with 3 setae. Infrasquamal setae present. Scutellum black ground color, with tomentum concolorous with thorax; with five pairs marginal setae; apical scutellar setae 1/2 as long as subapical scutellars, sub-erect, arising above plane of marginal setae; basal scutellar 2/3 length of subapical setae; subapical setae convergent, lateral scutellar setae can be convergent or strongly divergent. Legs: black ground color; mid tibia with 2–3 strong anteroventral setae; tarsal claws and pulvilli as long as last tarsomere. Wings: R_1_ bare, R_4+5_ setulose from node to crossvein r-m. **Abdomen** (Fig. 12a, b): ground color black; ST1+2 glabrous black, pale silver tomentum occupying anterior 75% of T3 and T4, T5 pale brassy-grey tomentose over entirety; tomentum extending to ventral surface of abdomen, but not completely; median marginal setae present on T3 and complete rows on T4 and T5.

**Terminalia** (Fig. 13): posterior margin of sternite 5 with a deeply excavated U-shaped median cleft (Fig. 13d); lateral lobes of sternite apically rounded, with a vestiture of short setae along disc, 2x as long along caudal margin; basal section 1/2 the length of apical lobes. Cerci, in posterior view, sharply pointed and slightly medially separated and parallel, almost touching medially, with a few short setae on basal half (Fig. 13a). In lateral view, curved and sharply tapered apically (Fig. 13b). Surstylus well-developed, stout basally in lateral view, broadly rounded; in posterior view, basally enlarged and apically straight.

Female (Fig. 14) as in male, except in the following aspects: **head** (Fig. 14c, d): fronto-orbital wider than males, just over 1/2 of head width entirely bright silver tomentose, frontal vitta 2/5 width of fronto-orbital plate; overall head of females 1.5x wider than that of males; legs (Fig. 14b): mid-tibia with 3–4 irregularly-sized strong anteroventral setae.

#### Diagnosis

*Metaplagia svetlanakozikae* **sp. n.** can be distinguished from all other *Metaplagia* by the following combination of traits: frontal vitta indistinct around ocellar triangle, infrasquamal setae present, fronto-orbital plate at pale gray or silver, with a silver parafacial and setulae on vein R_4+5_ not extending beyond crossvein r-m, surstylus rounded at tip. *Metaplagia svetlanakozikae* **sp. n.** can be separated from *Metaplagia occidentalis* by the presence of infrasquamal setae and from *Metaplagia lindarobinsonae* **sp. n.** by the presence of a small circular formation of setulae on the parafacial and not distinct row of setulae along the facial ridge. *Metaplagia svetlanakozikae* **sp. n.** is clearly distinguished by its COI sequence clustered within the Barcode Identification Number (BIN) BOLD:AAD5456.

#### Etymology

*Metaplagia svetlanakozikae* **sp. n.** is named in honor of Svetlana Kozik for her years of coordinating and administrating a plethora of problems and events for the undergraduate biology teaching laboratories of the Department of Biology, University of Pennsylvania, Philadelphia, Pennsylvania.

#### Distribution

Costa Rica, ACG, Guanacaste Province, 10–280 m elevation.

#### Ecology

*Metaplagia svetlanakozikae* **sp. n.** has been reared seven times from one species of Lepidoptera in the family Sphingidae: *Agrius cingulata*, in dry forest.

## Identification Keys

### Key to the Metaplagia of North and Mesoamerica

**Table:** 

1	Setulae present on wing vein R_4+5_ not extending beyond crossvein r-m	2
–	Setulae present on wing vein R_4+5_ extending beyond crossvein r-m	8
2	Fronto-orbital plate and parafacial entirely and uniformly gold	* paulinesaribasae * **sp. n.**
–	Fronto-orbital plate at most with some gold present and parafacial not gold	3
3	Fronto-orbital plate bearing some gold	* leahdennisae * **sp. n.**
–	Fronto-orbital plate pale grey or silver (can include pale brassy) distinctly not gold	4
4	Infrasquamal setae absent	*occidentalis* Coquillett, 1895
–	Infrasquamal setae present	5
5	Frontal vitta indistinct around ocellar triangle, not reaching occiput	6
–	Frontal vitta wide and prominent extending around ocellar triangle to occiput	7
6	Parafacial setulae descending along parafacial in a row near facial ridge; surstylus sharp at tip	* lindarobinsonae * **sp. n.**
–	Parafacial setulae present as a small circular grouping along upper 1/3 of parafacial, not in a row near facial ridge; surstylus rounded at tip	* svetlanakozikae * **sp. n.**
7	Postpedicel just over 3 times as long as pedicel; claws and pulvilli shorter than last tarsomere	*brevicornis* Brooks, 1945
–	Postpedicel 2 times as long as pedicel; claws and pulvilli longer than last tarsomere	*orientalis* Townsend, 1915
8	Fronto-orbital plate gold on at least upper two thirds	* robinsherwoodae * **sp. n.**
–	Fronto-orbital plate entirely silver	9
9	Wing vein R_1_ bare throughout	*facialis* Reinhard, 1956
–	Wing vein R_1_ setulose throughout	*cordata* Reinhard, 1960

## Supplementary Material

XML Treatment for Metaplagia

XML Treatment for Metaplagia leahdennisae

XML Treatment for Metaplagia lindarobinsonae

XML Treatment for Metaplagia paulinesaribasae

XML Treatment for Metaplagia robinsherwoodae

XML Treatment for Metaplagia svetlanakozikae

## Figures and Tables

**Figure 2a.**
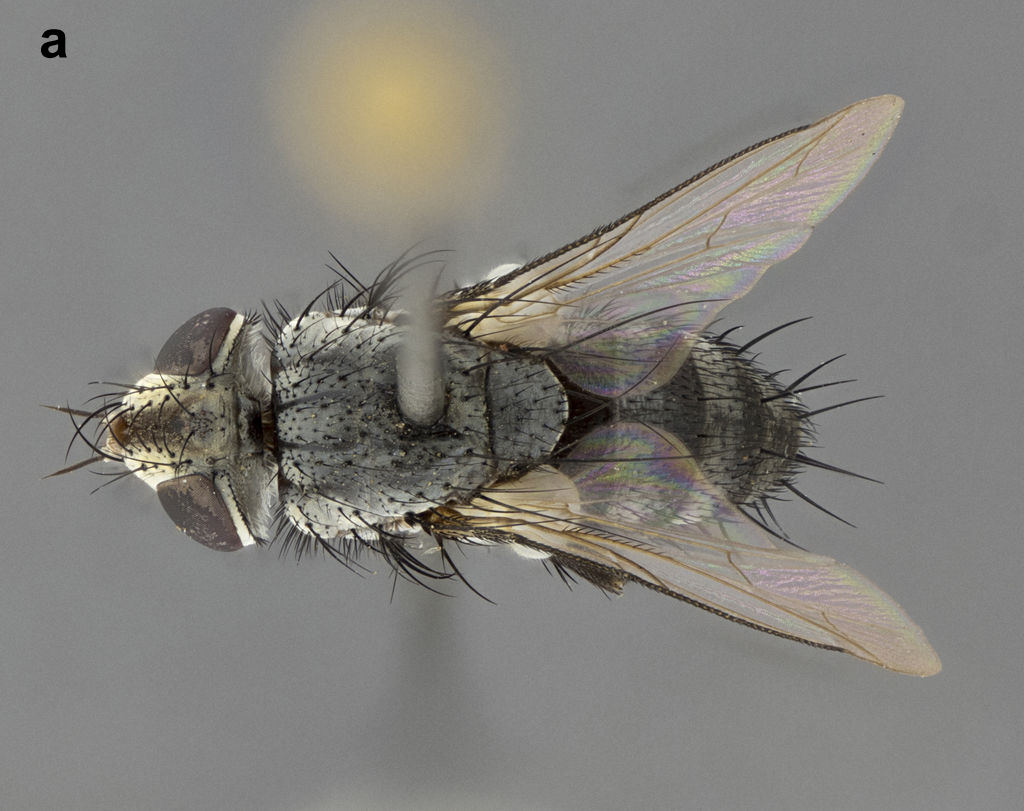
dorsal view

**Figure 2b.**
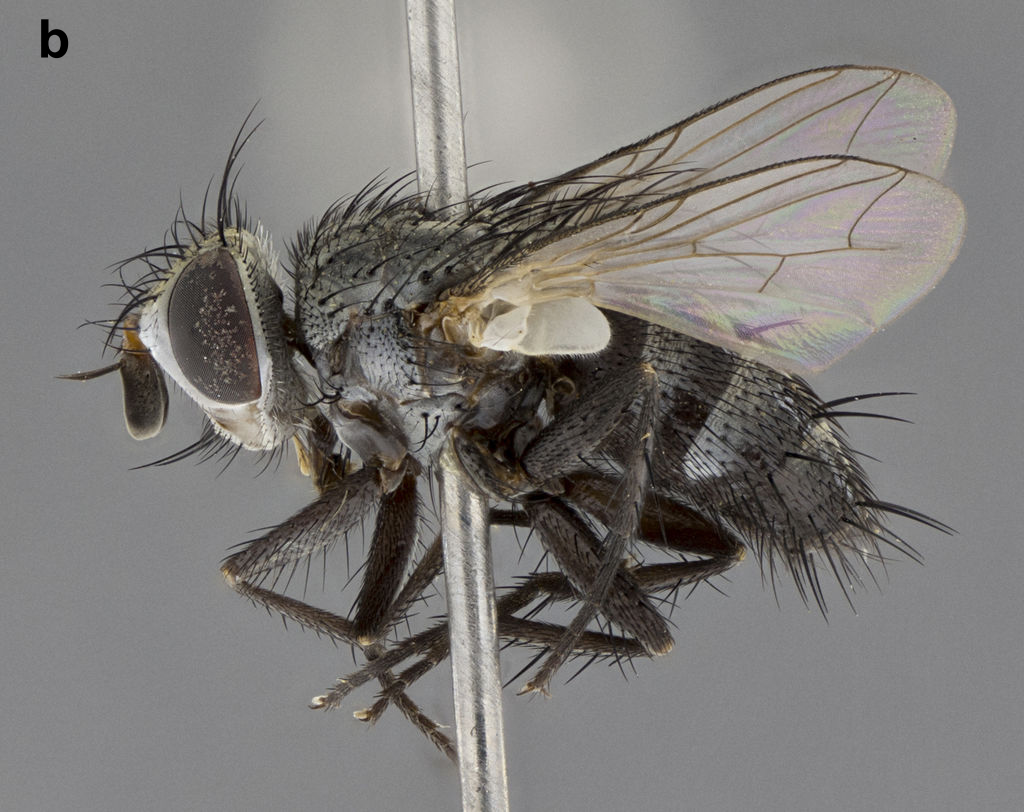
lateral view

**Figure 2c.**
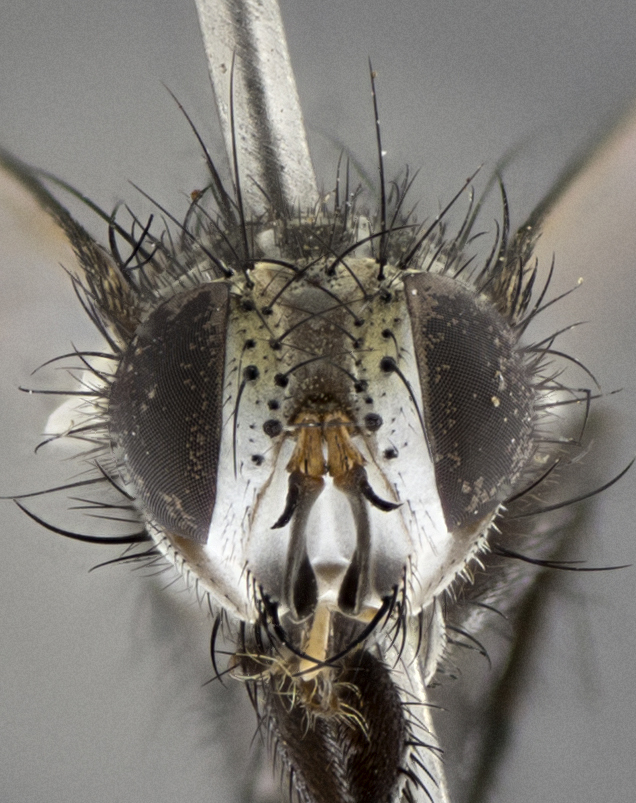
frontal view

**Figure 2d.**
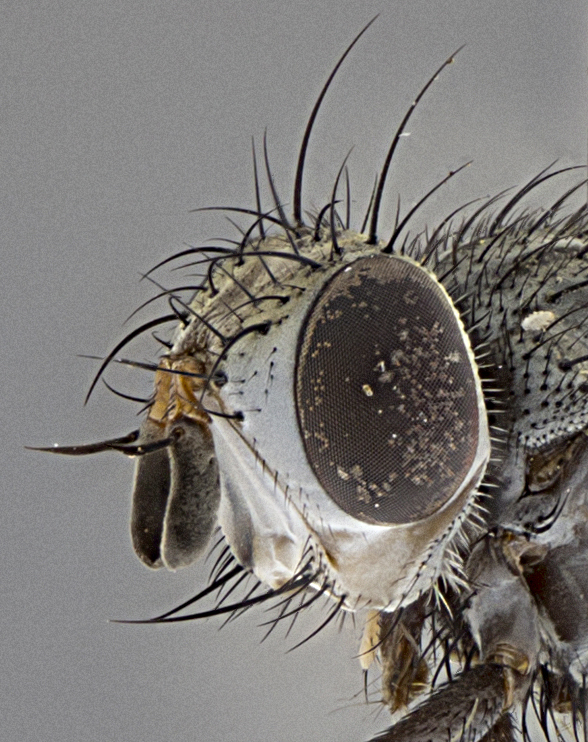
three quarters view

**Figure 3a.**
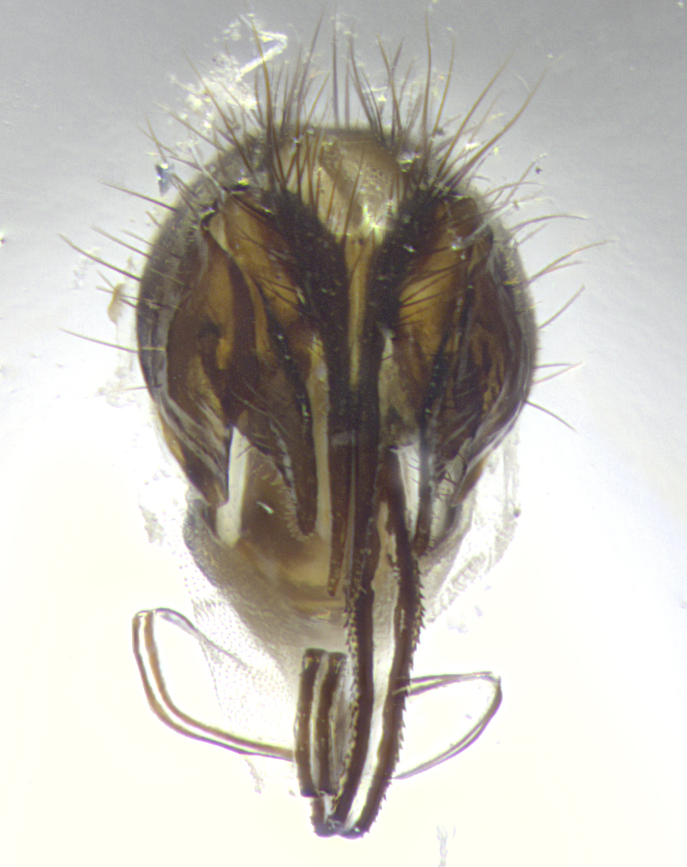
caudal view

**Figure 3b.**
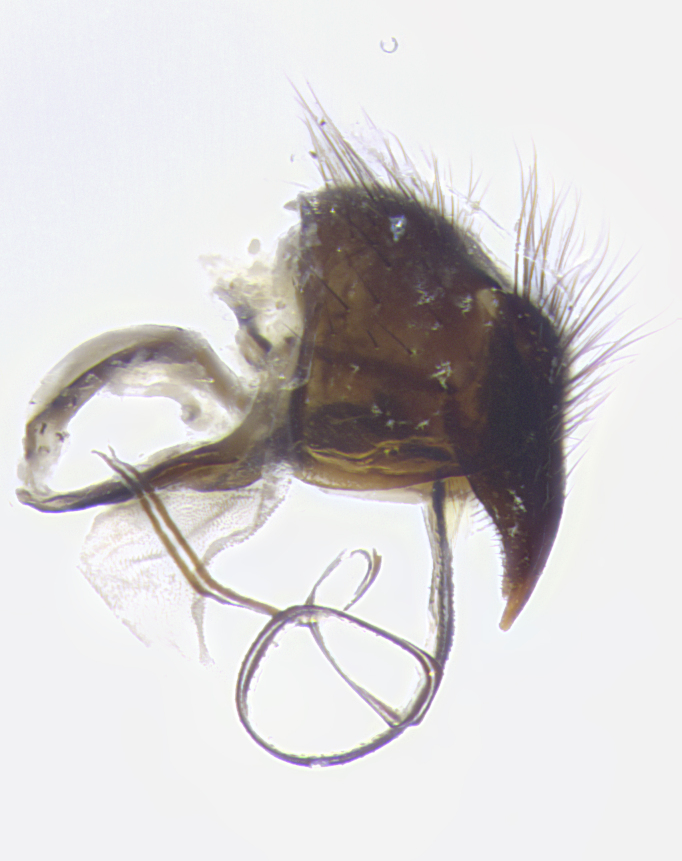
lateral view

**Figure 3c.**
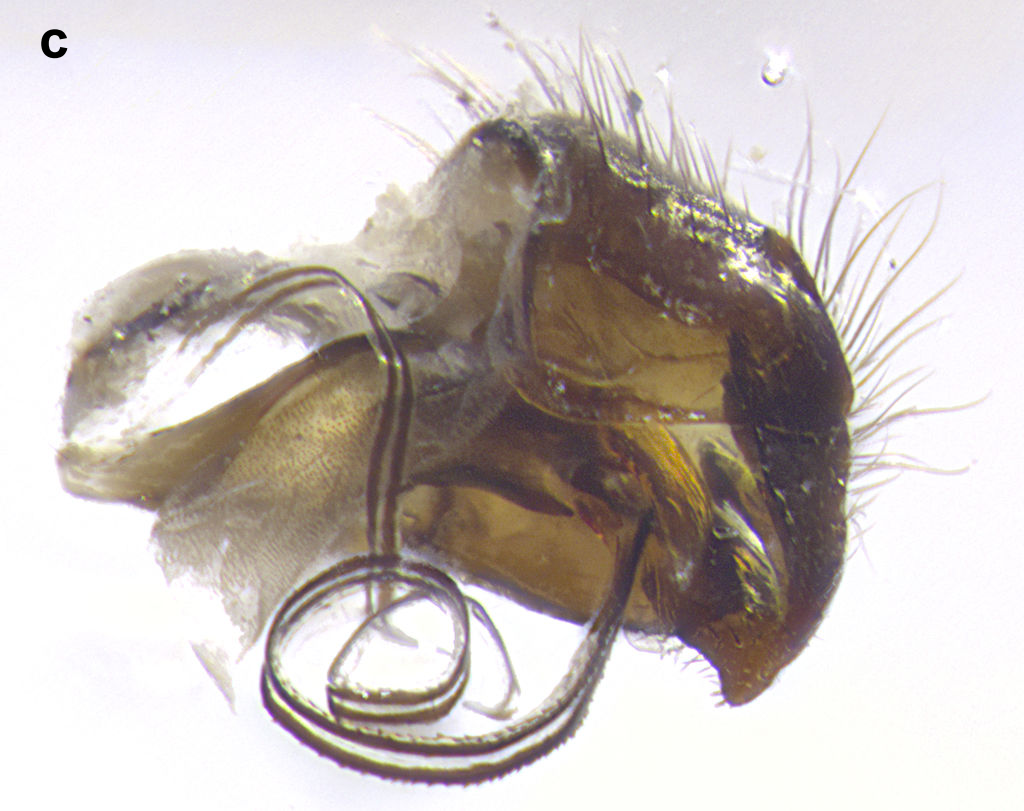
oblique view

**Figure 3d.**
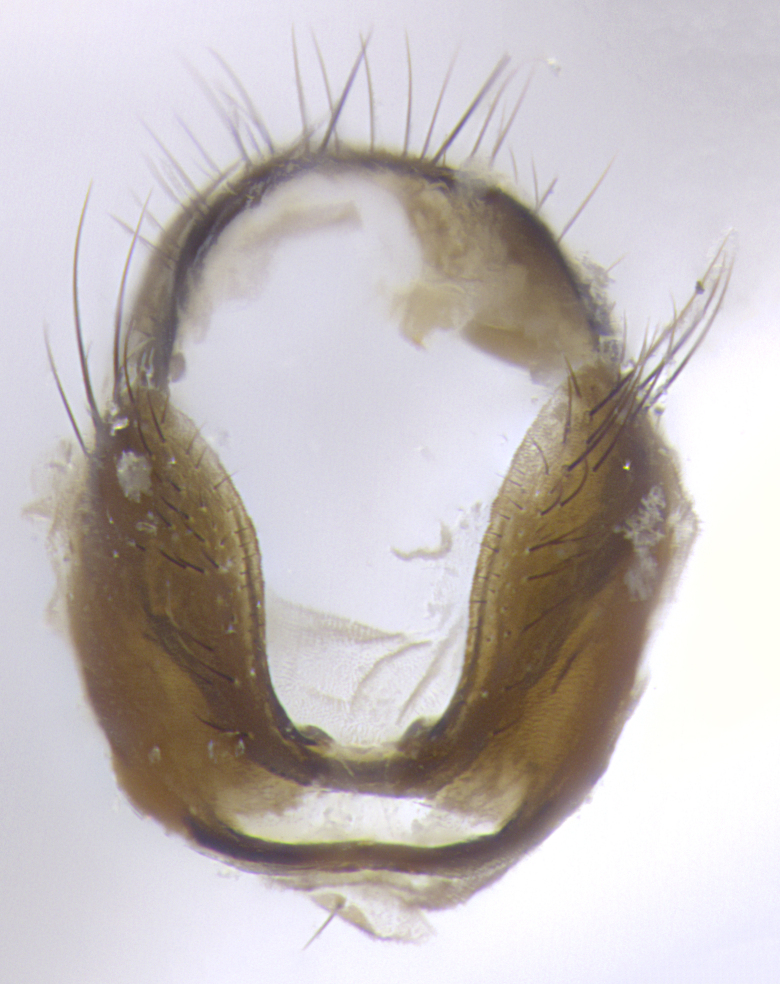
sternite 5, ventral view

**Figure 4a.**
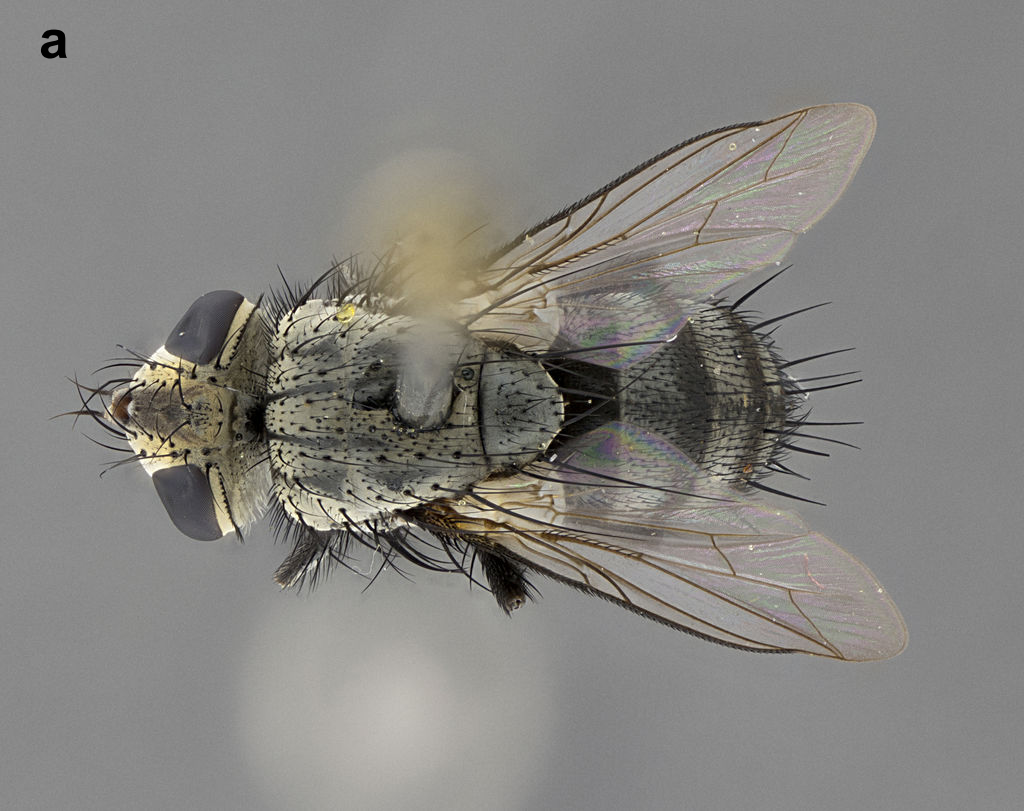
dorsal view

**Figure 4b.**
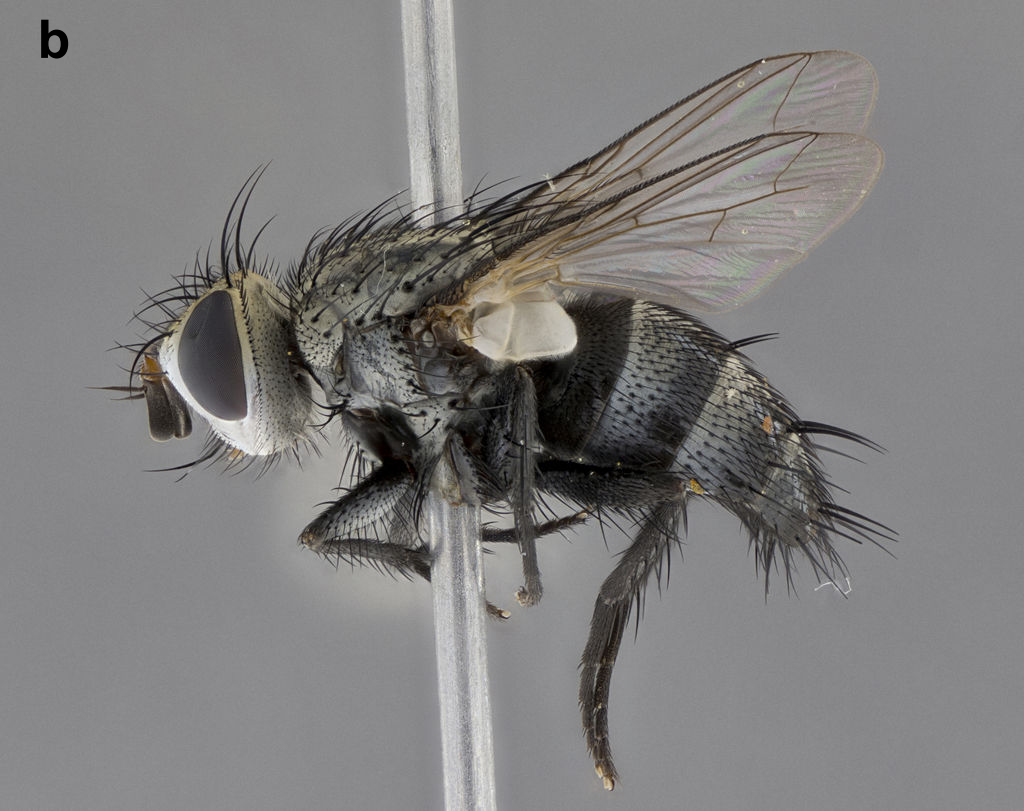
lateral view

**Figure 4c.**
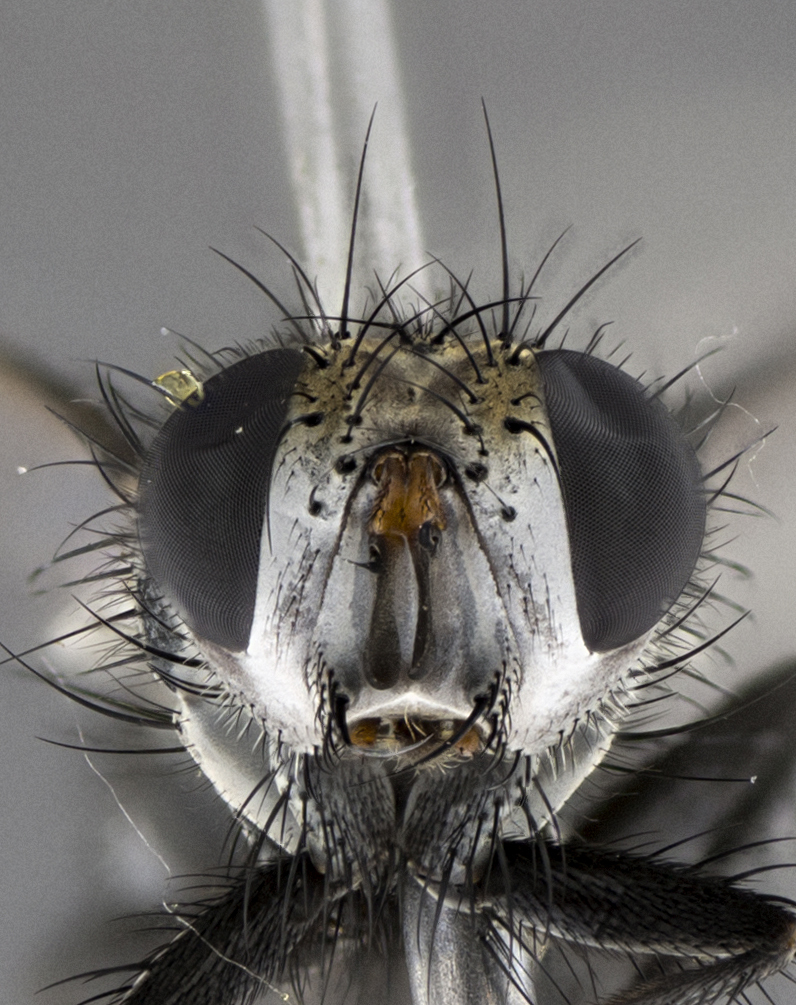
frontal view

**Figure 4d.**
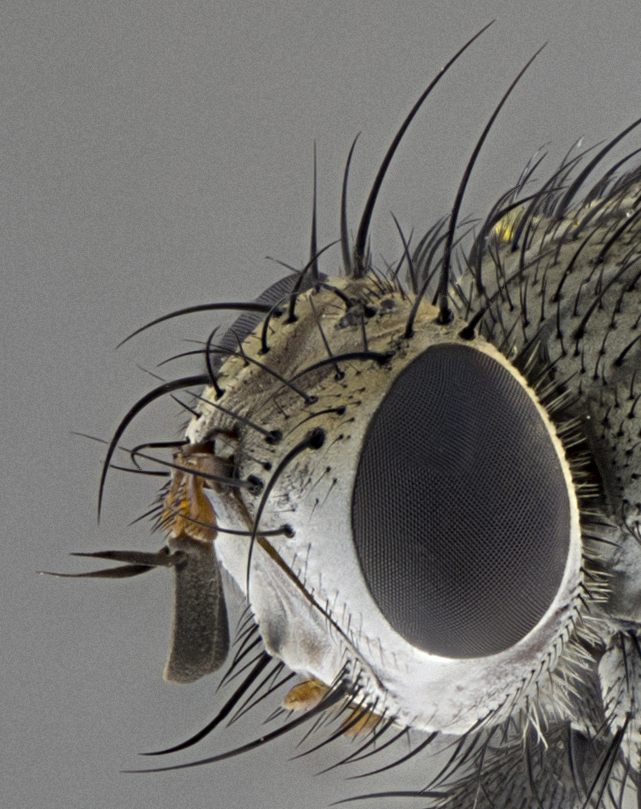
three quarters view

**Figure 5a.**
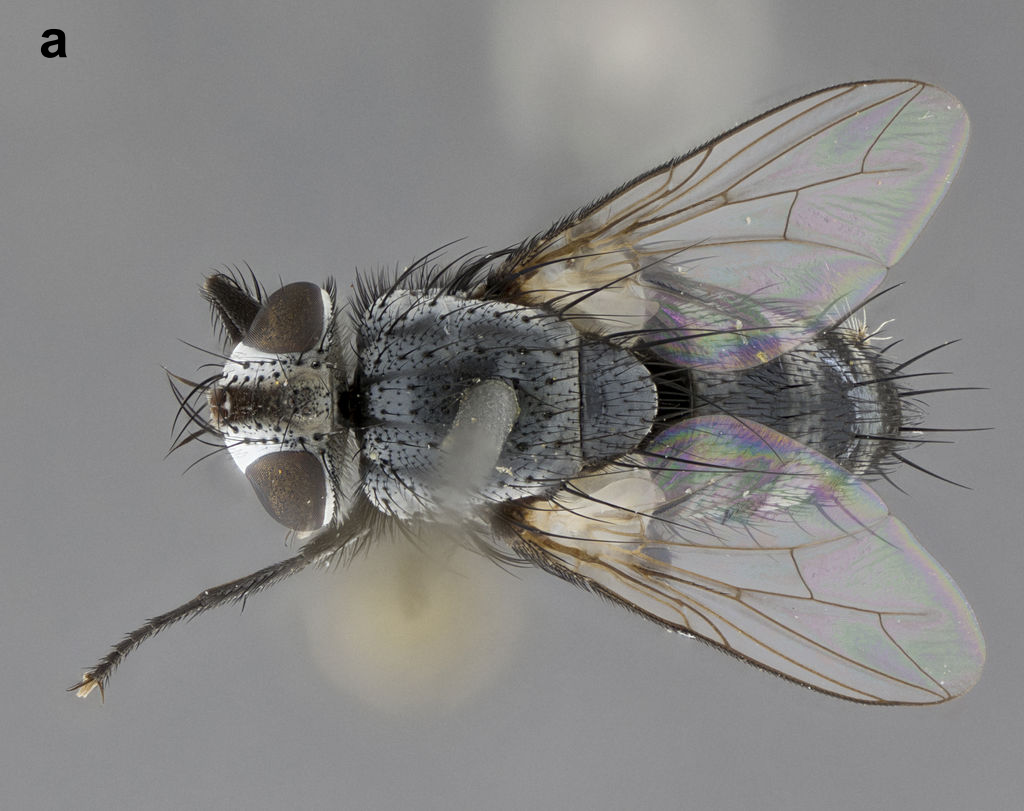
dorsal view

**Figure 5b.**
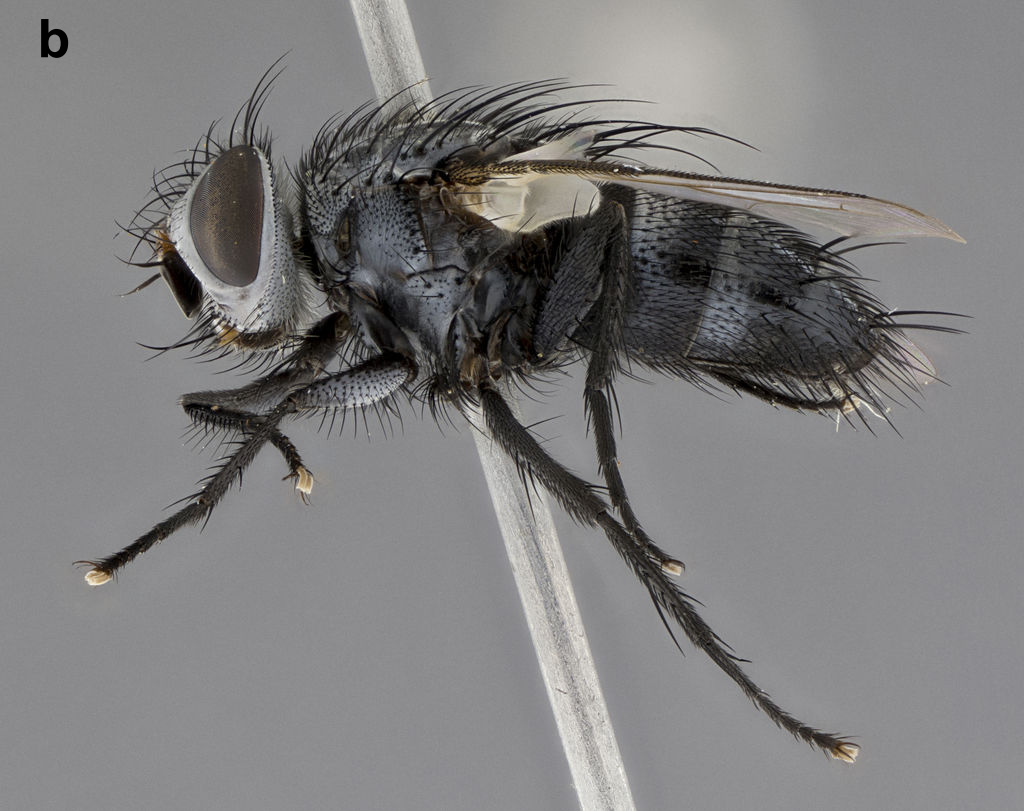
lateral view

**Figure 5c.**
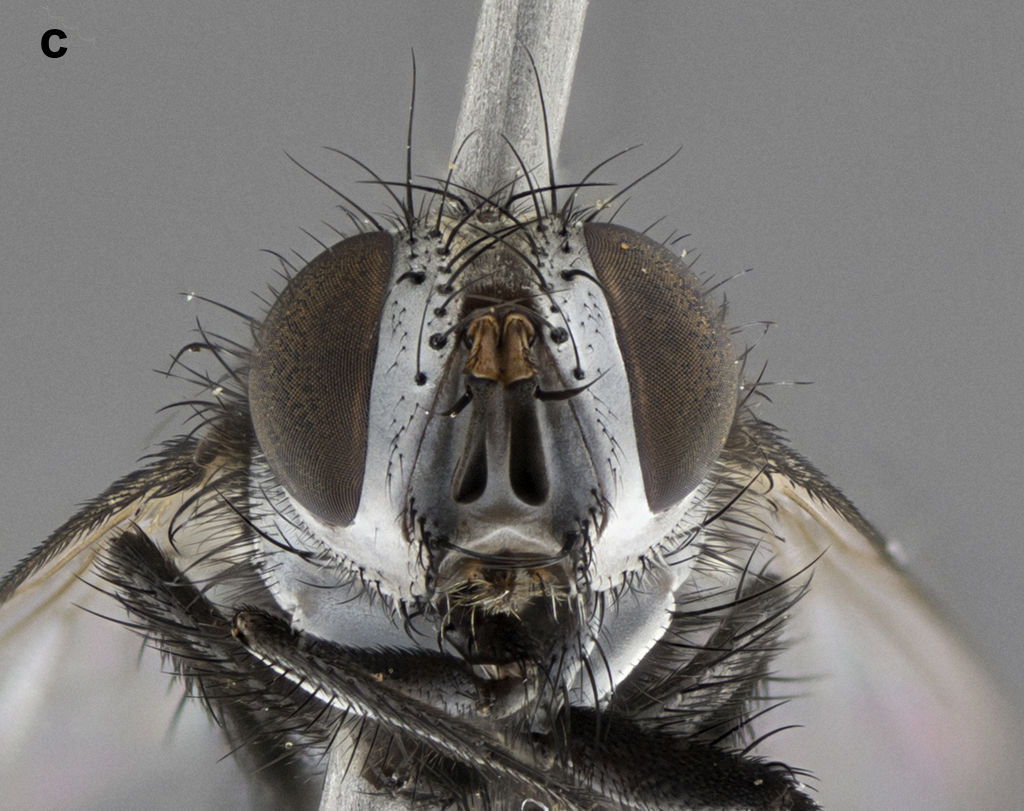
frontal view

**Figure 5d.**
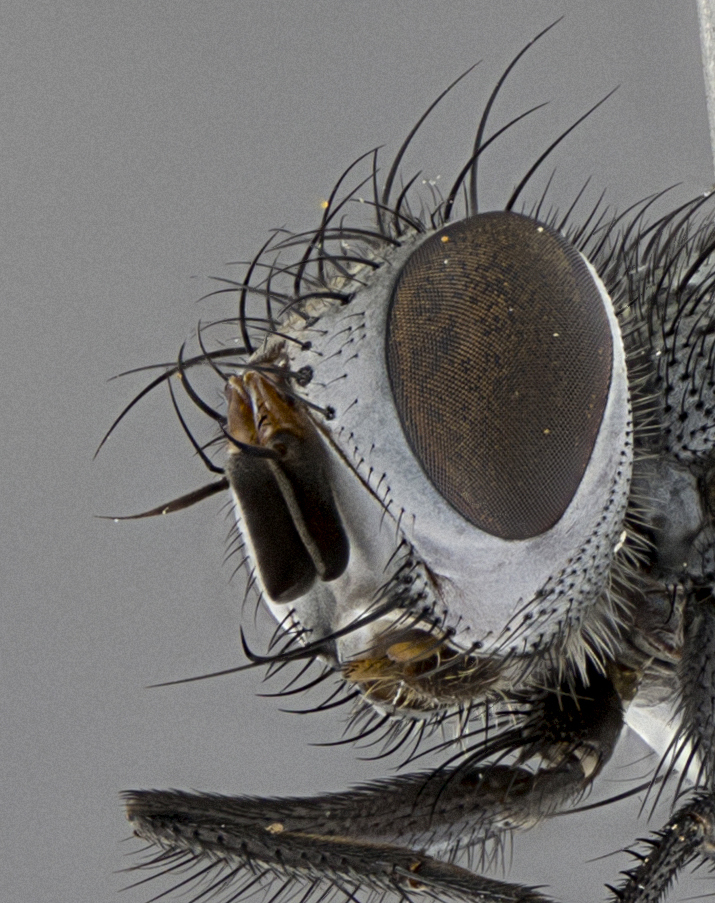
three quarters view

**Figure 6a.**
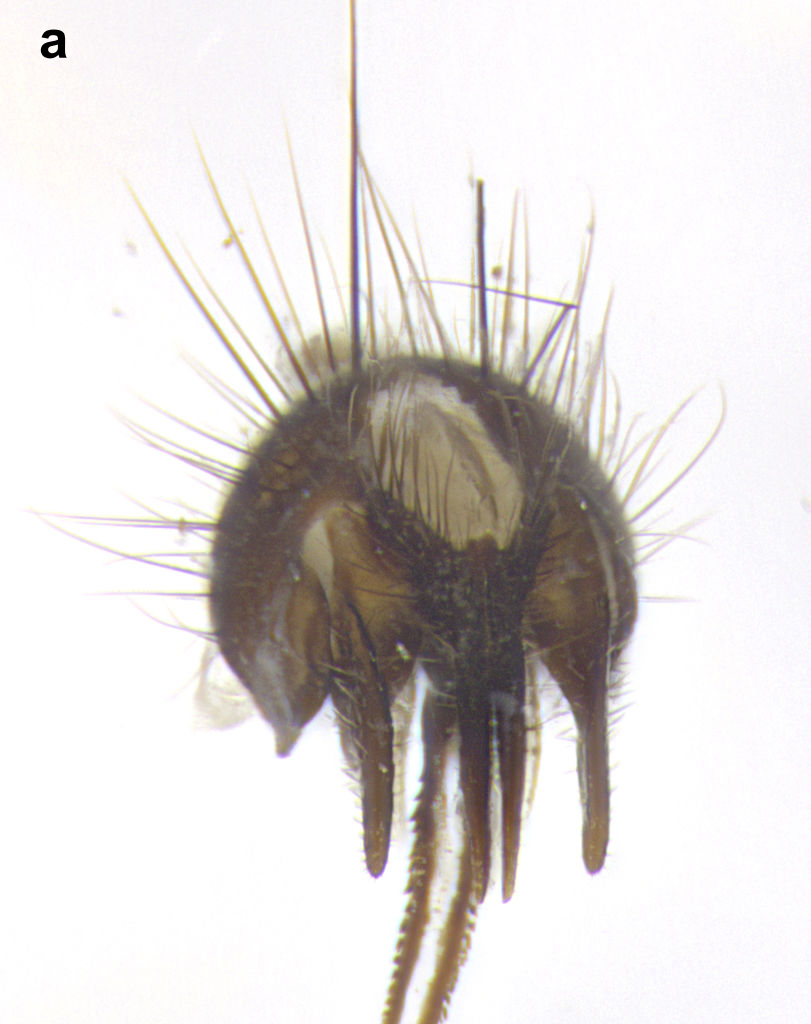
caudal view

**Figure 6b.**
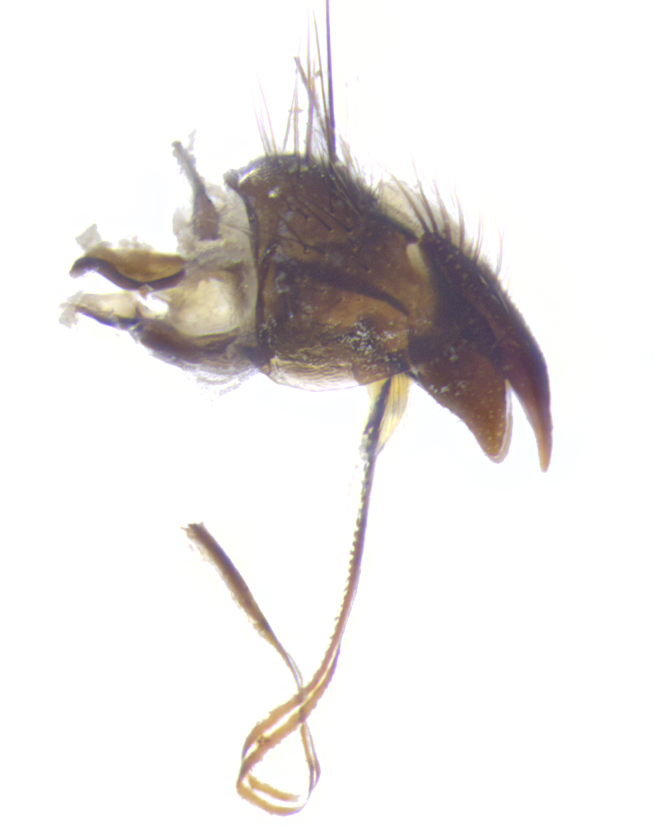
lateral view

**Figure 6c.**
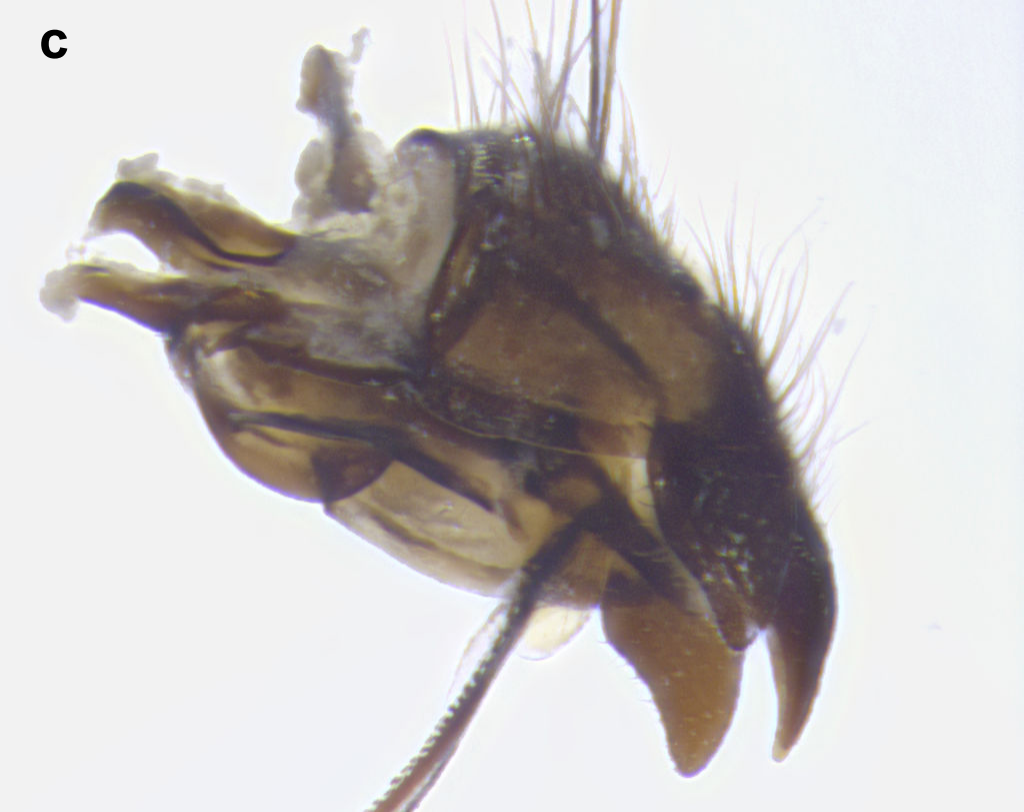
oblique view

**Figure 6d.**
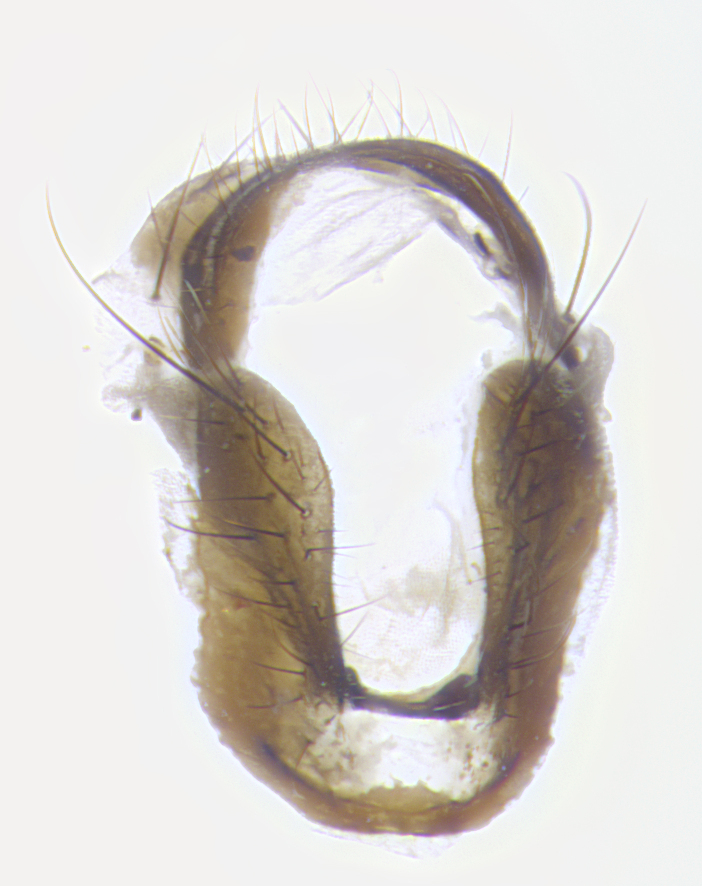
sternite 5, ventral view

**Figure 7a.**
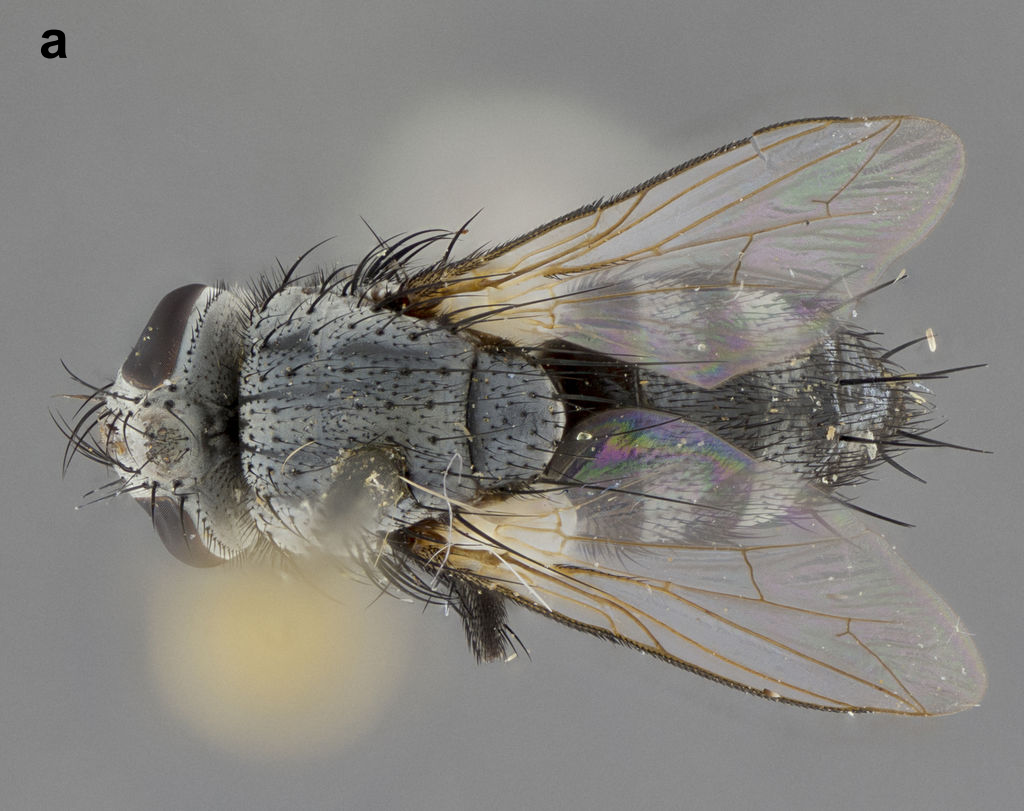
dorsal view

**Figure 7b.**
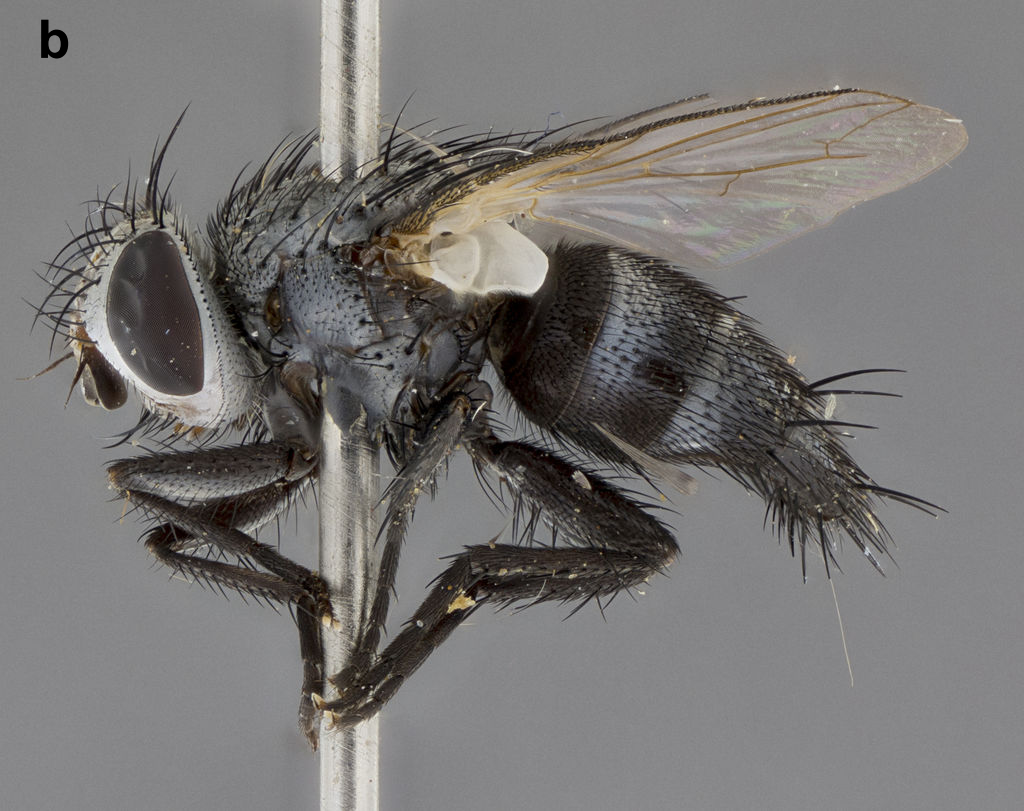
lateral view

**Figure 7c.**
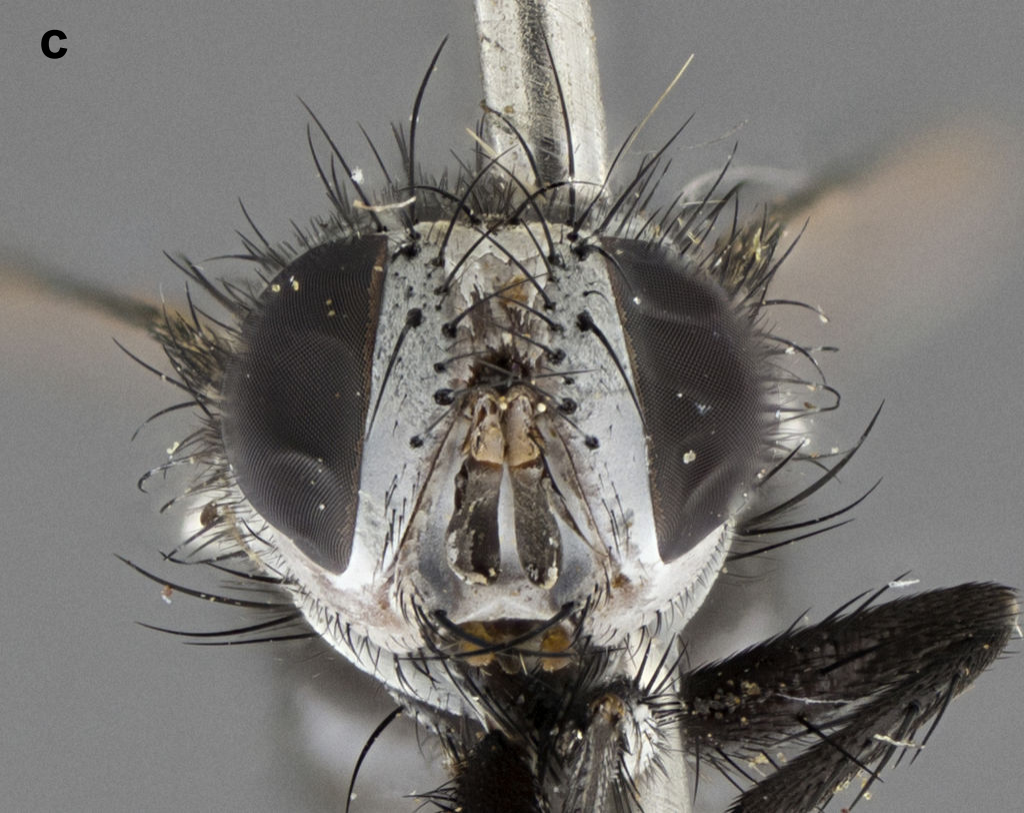
frontal view

**Figure 7d.**
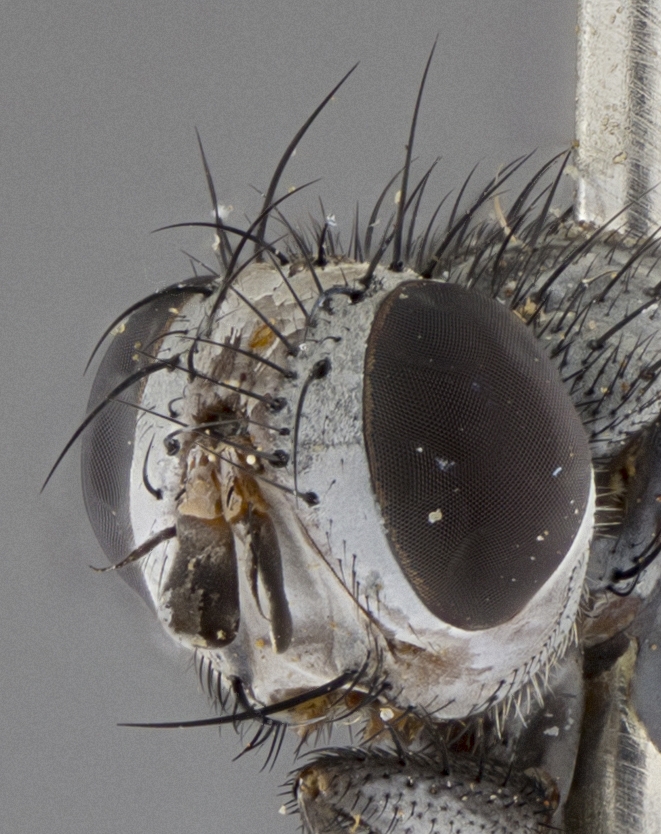
three quarters view

**Figure 8a.**
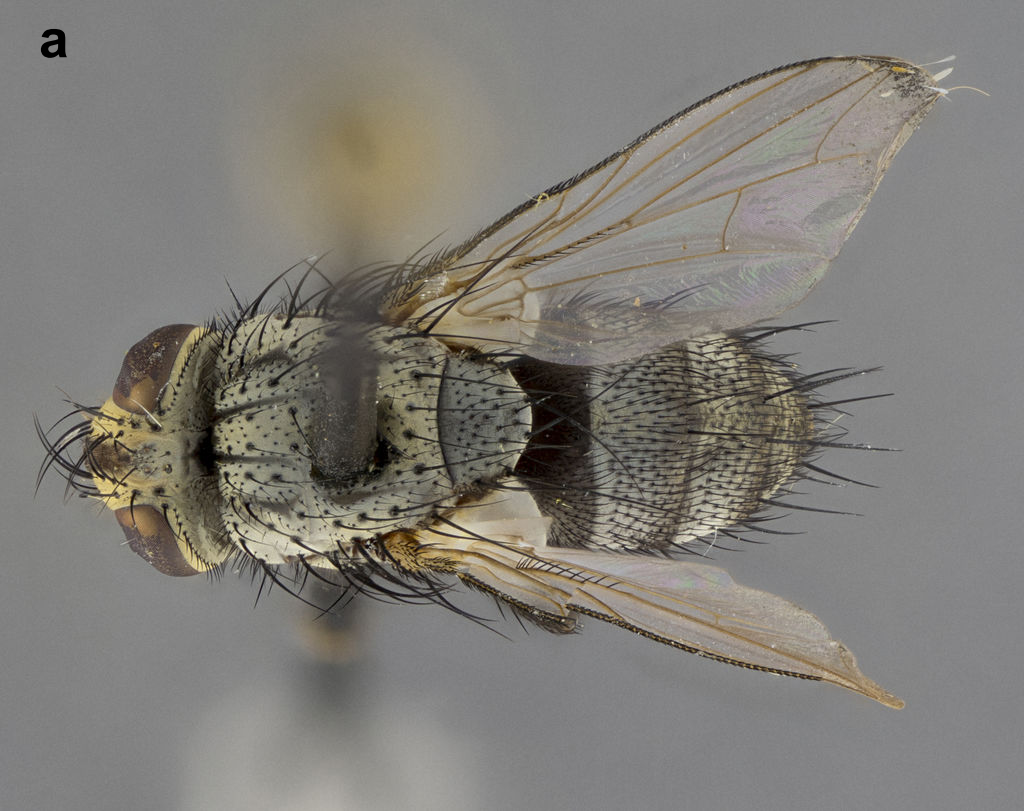
dorsal view

**Figure 8b.**
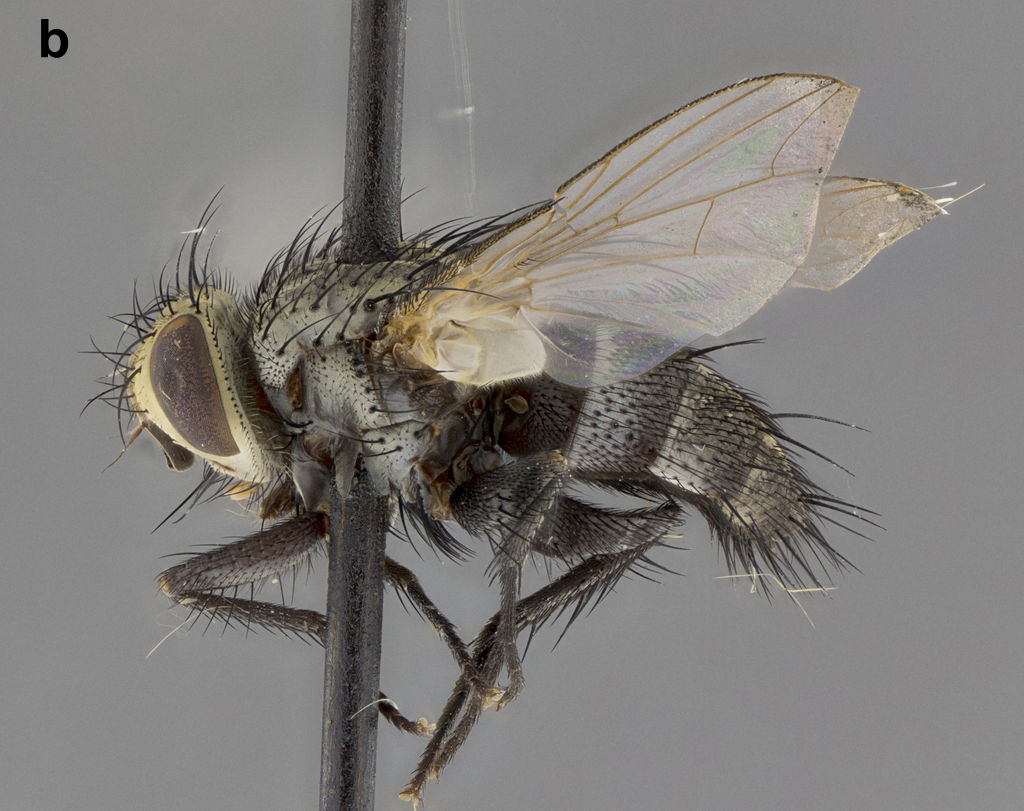
lateral view

**Figure 8c.**
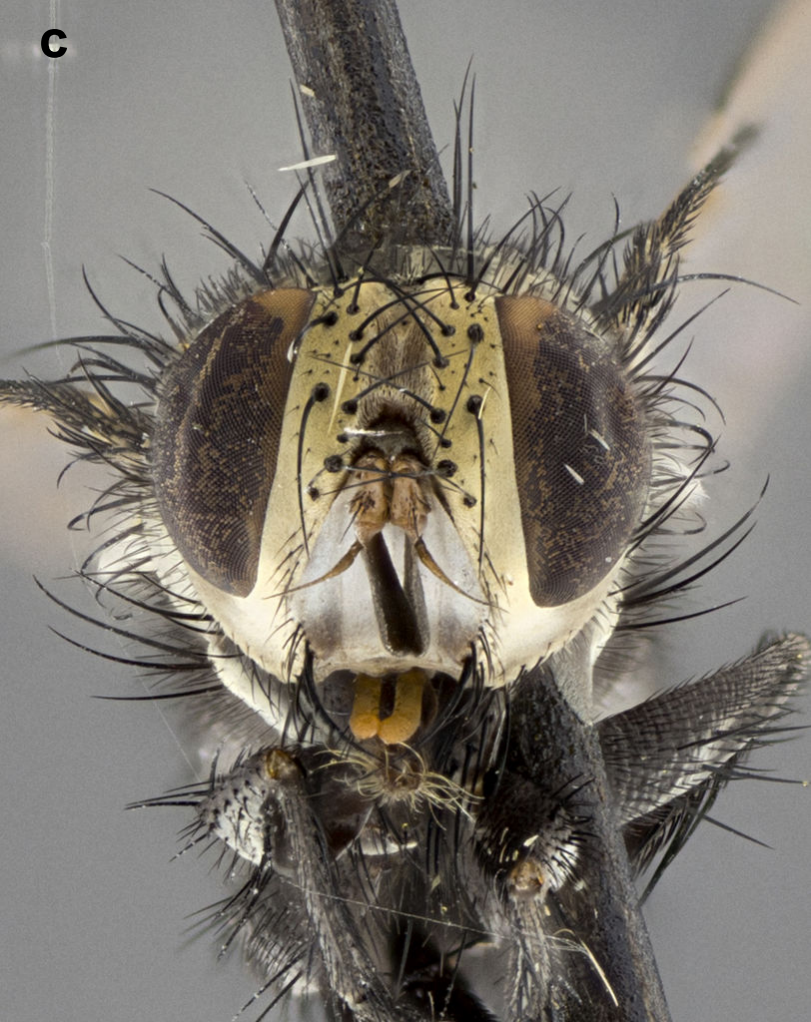
frontal view

**Figure 8d.**
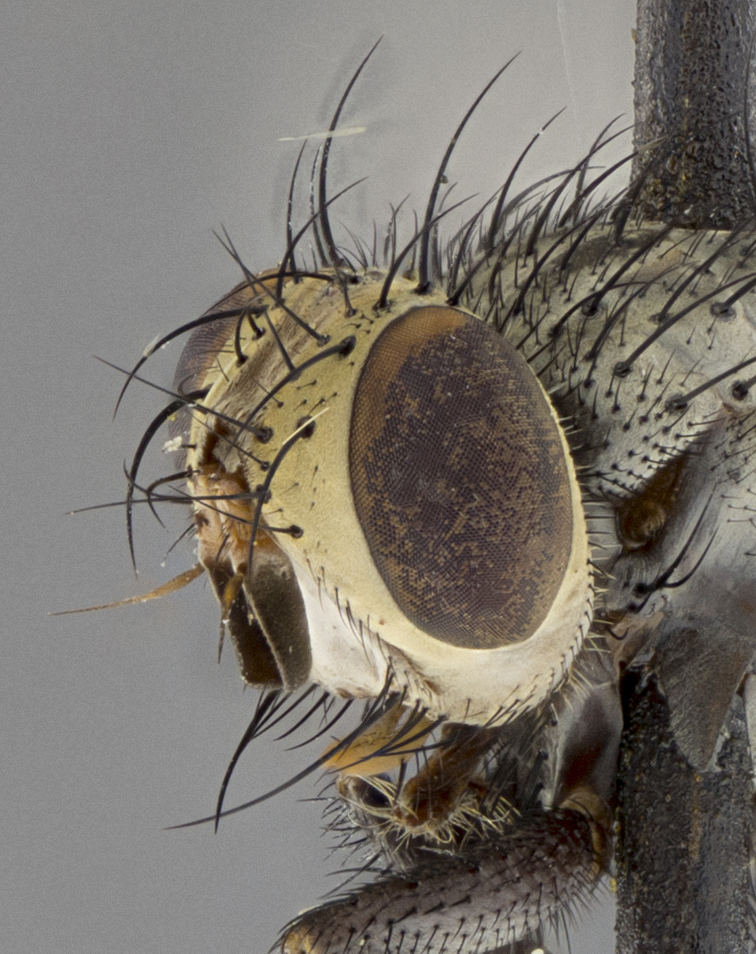
three quarters view

**Figure 9a.**
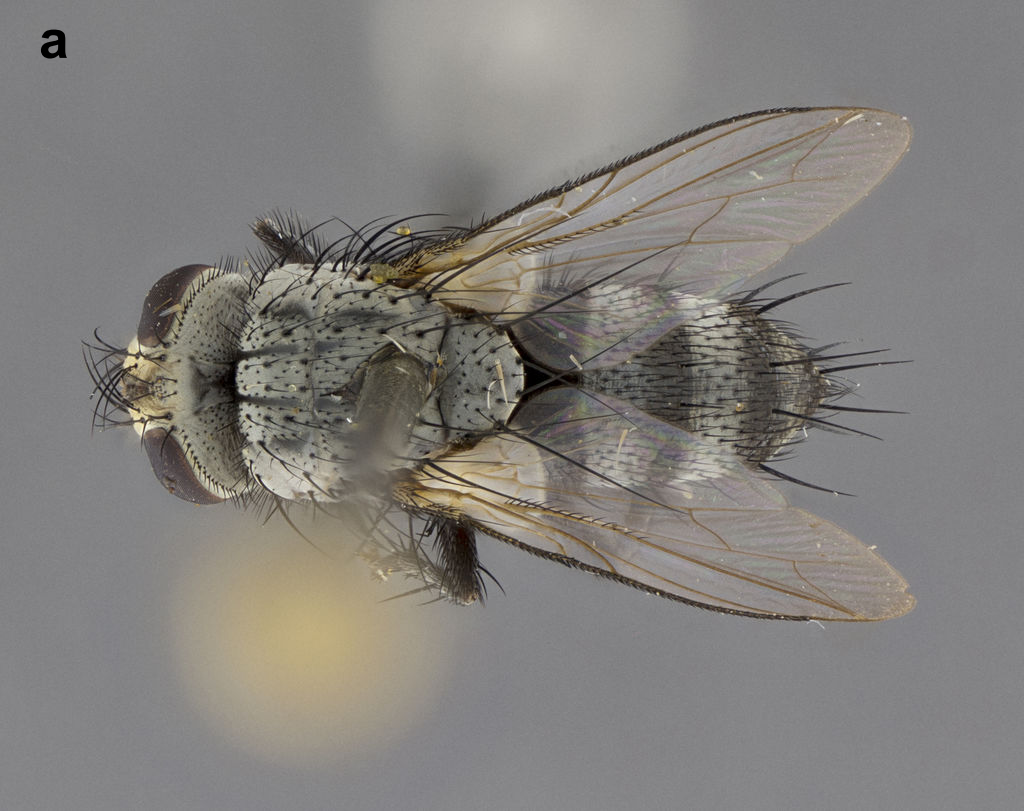
dorsal view

**Figure 9b.**
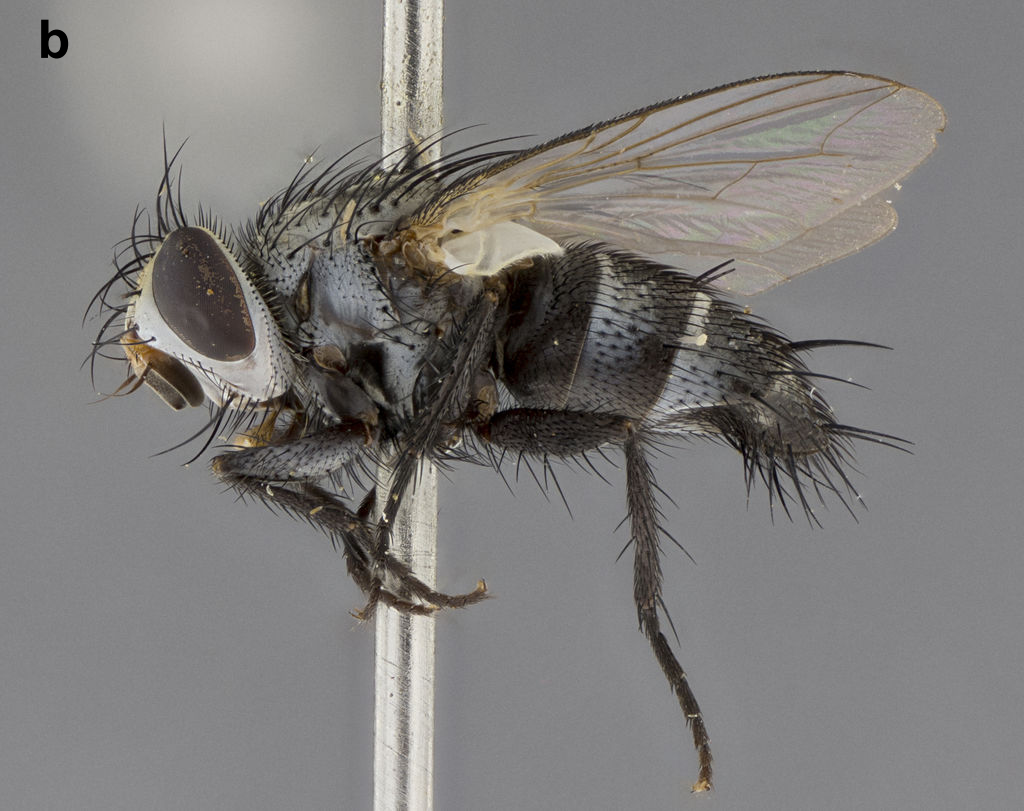
lateral view

**Figure 9c.**
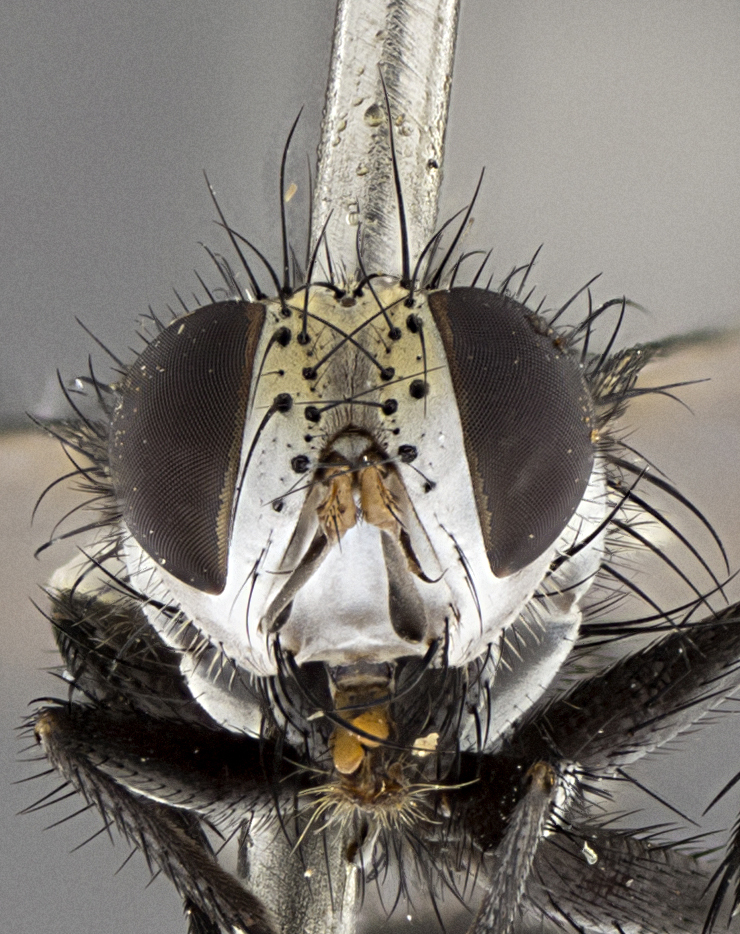
frontal view

**Figure 9d.**
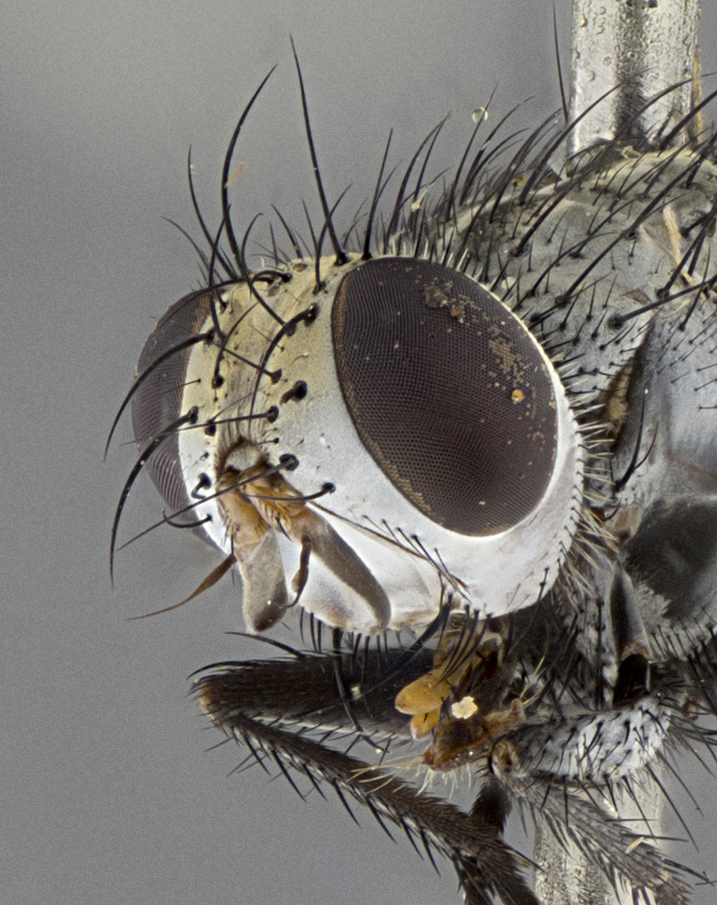
three quarters view

**Figure 10a.**
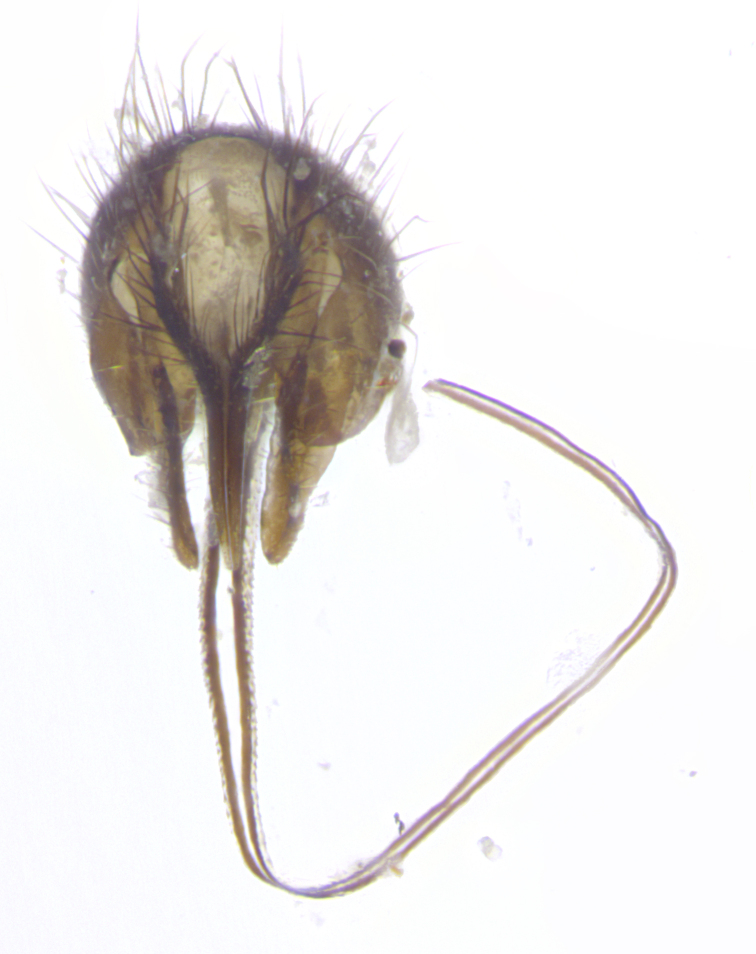
caudal view

**Figure 10b.**
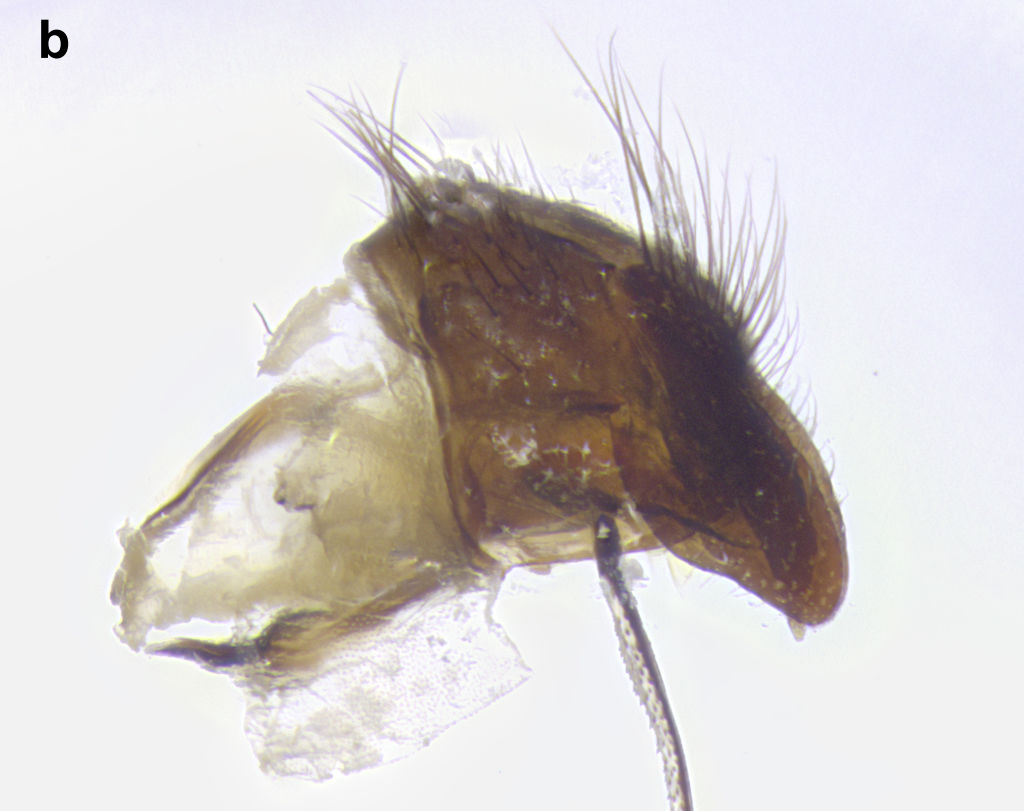
lateral view

**Figure 10c.**
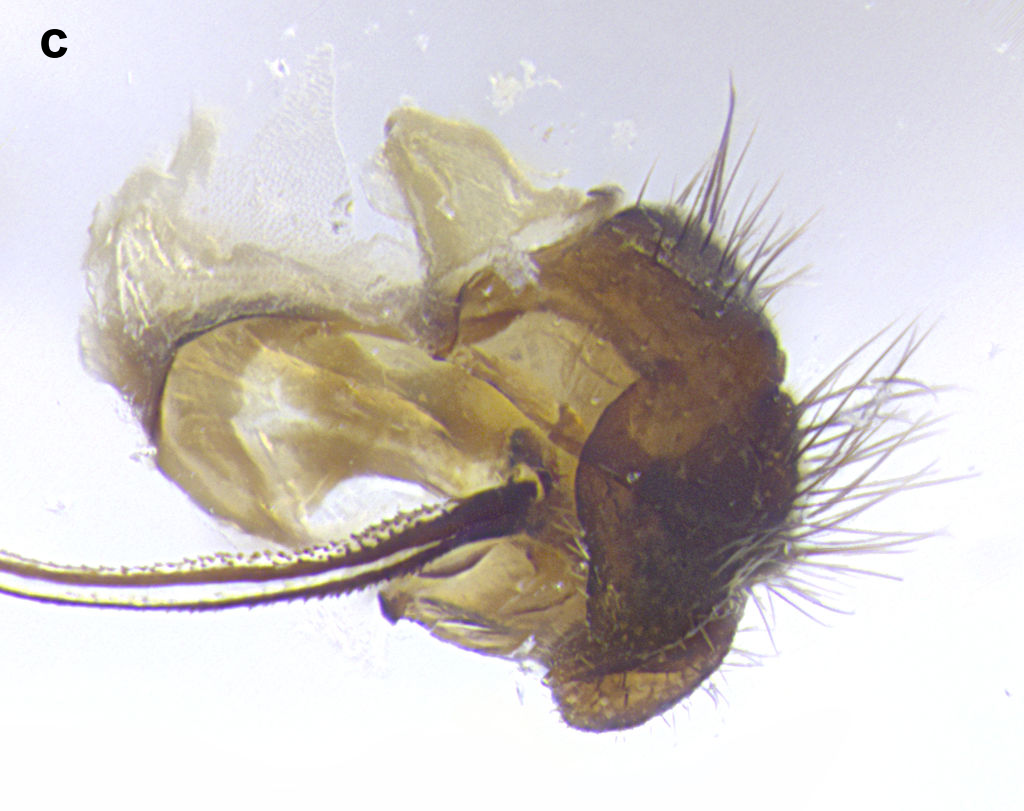
oblique view

**Figure 10d.**
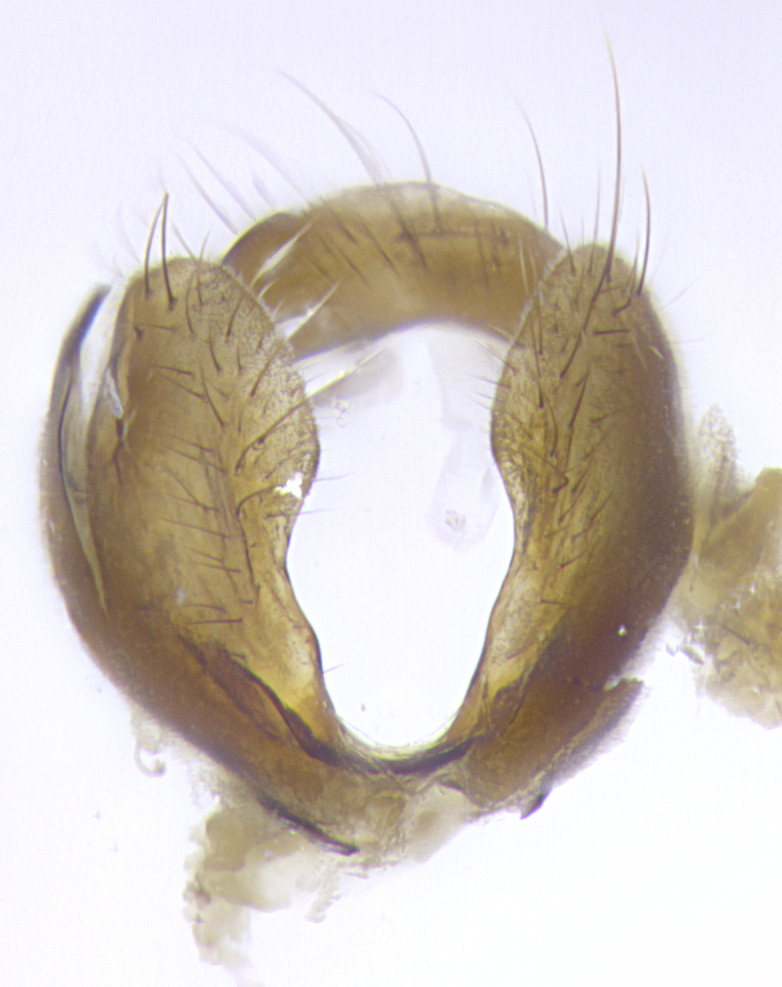
sternite 5, ventral view

**Figure 11a.**
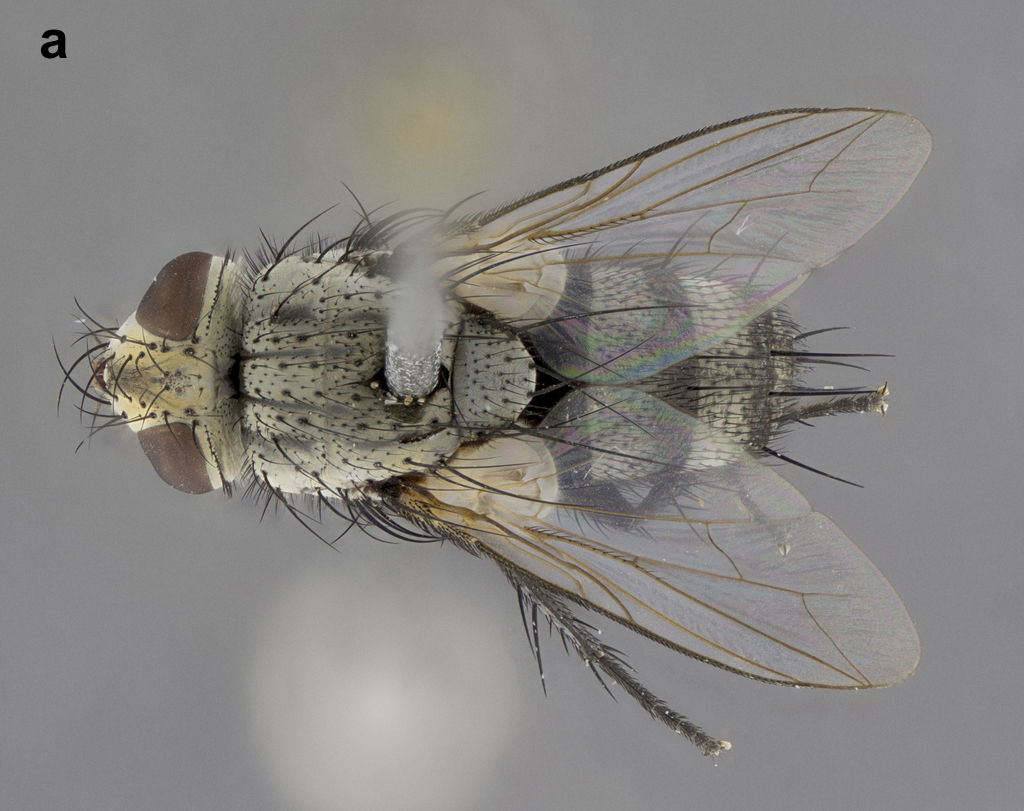
dorsal view

**Figure 11b.**
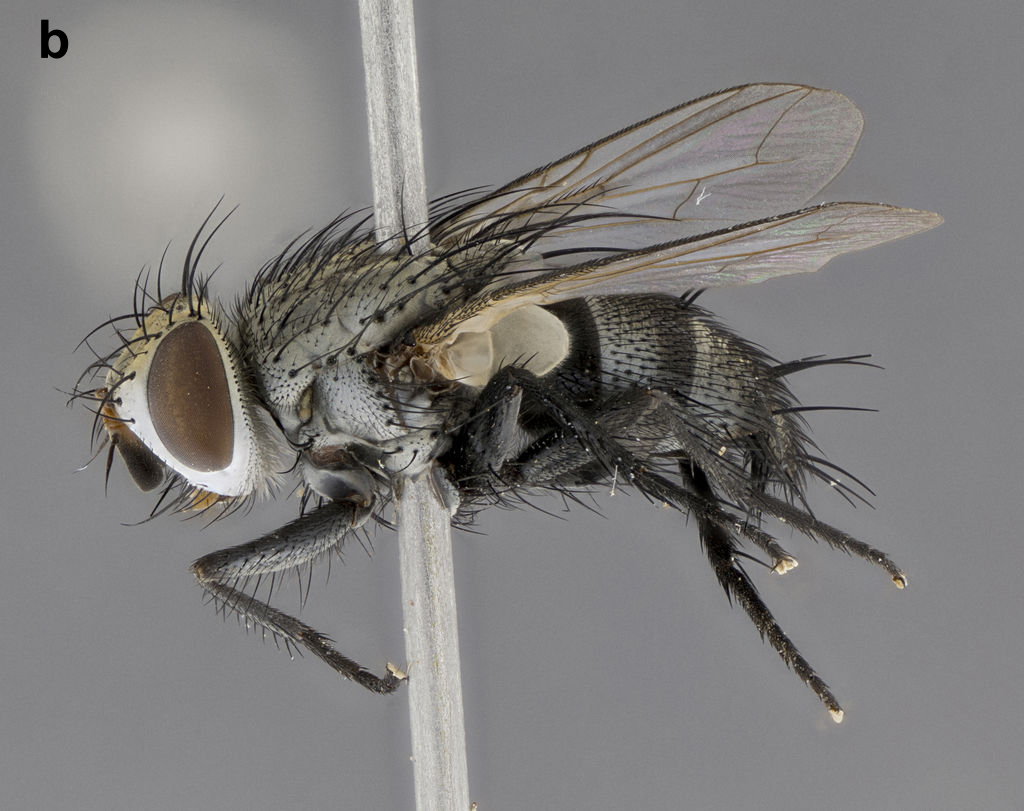
lateral view

**Figure 11c.**
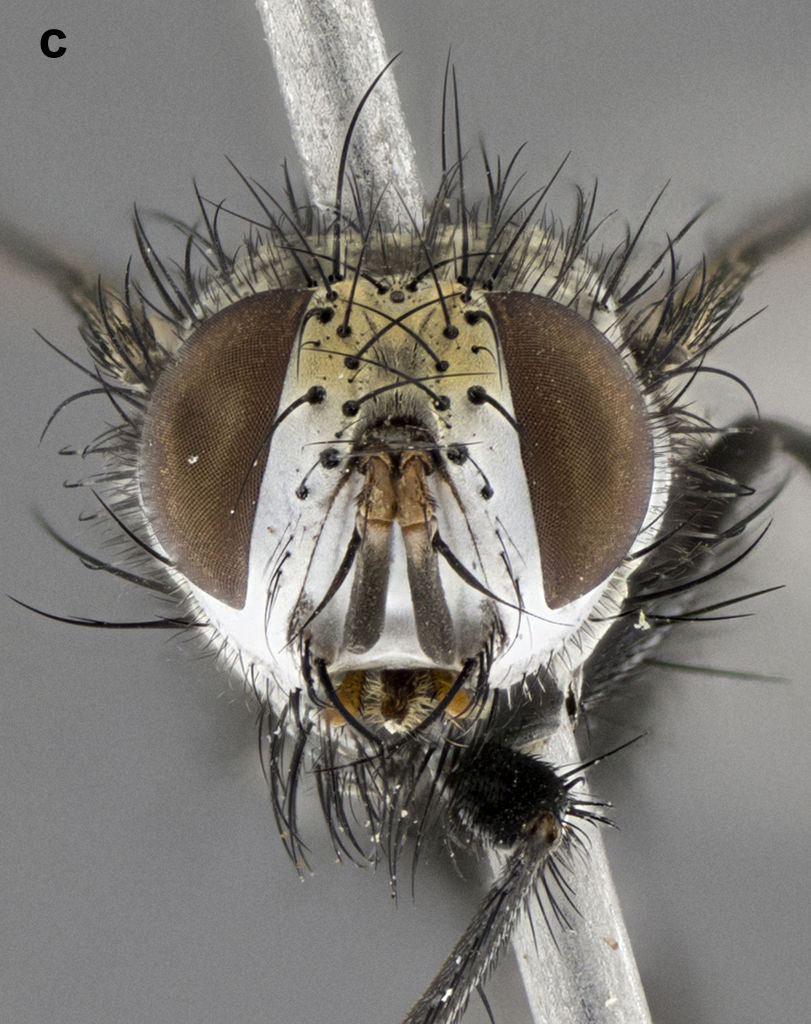
frontal view

**Figure 11d.**
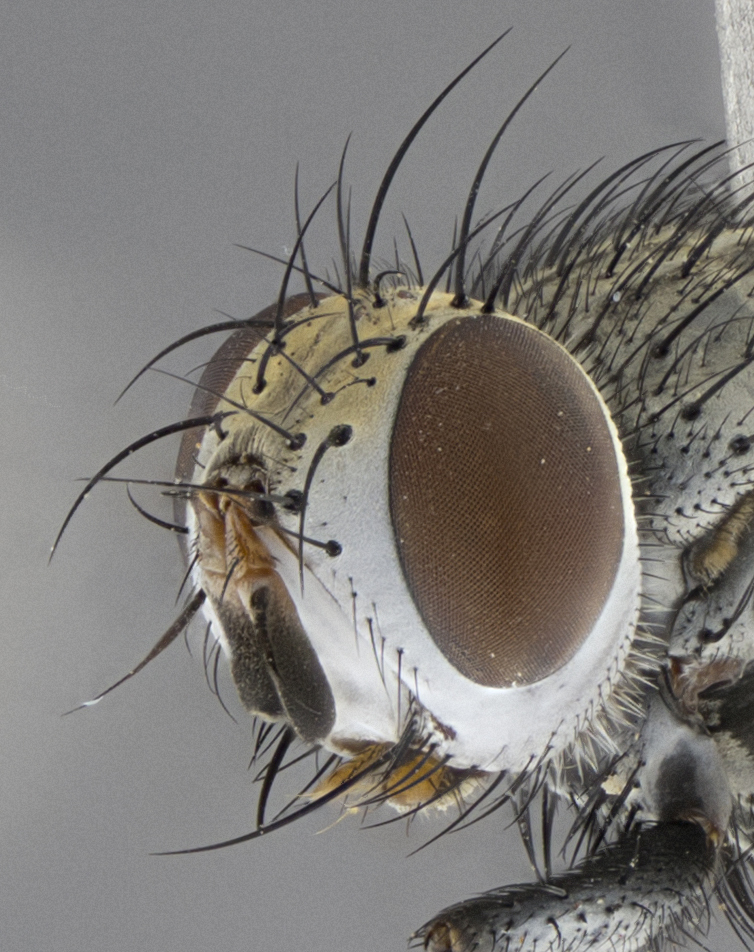
three quarters view

**Figure 12a.**
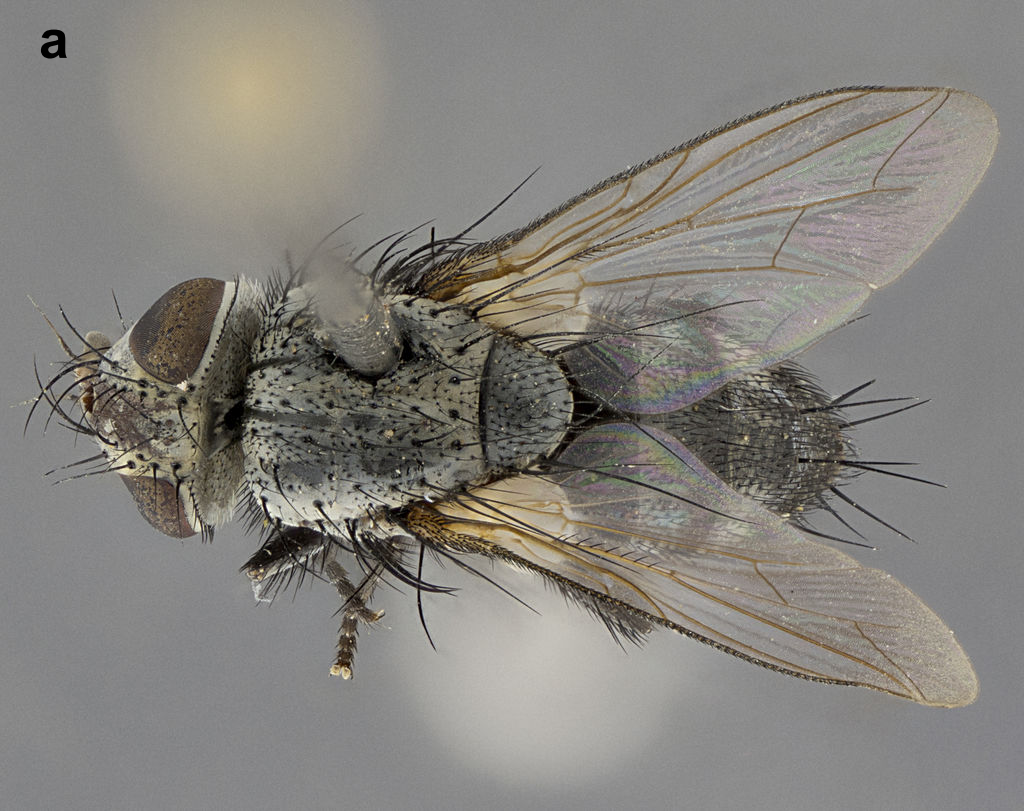
dorsal view

**Figure 12b.**
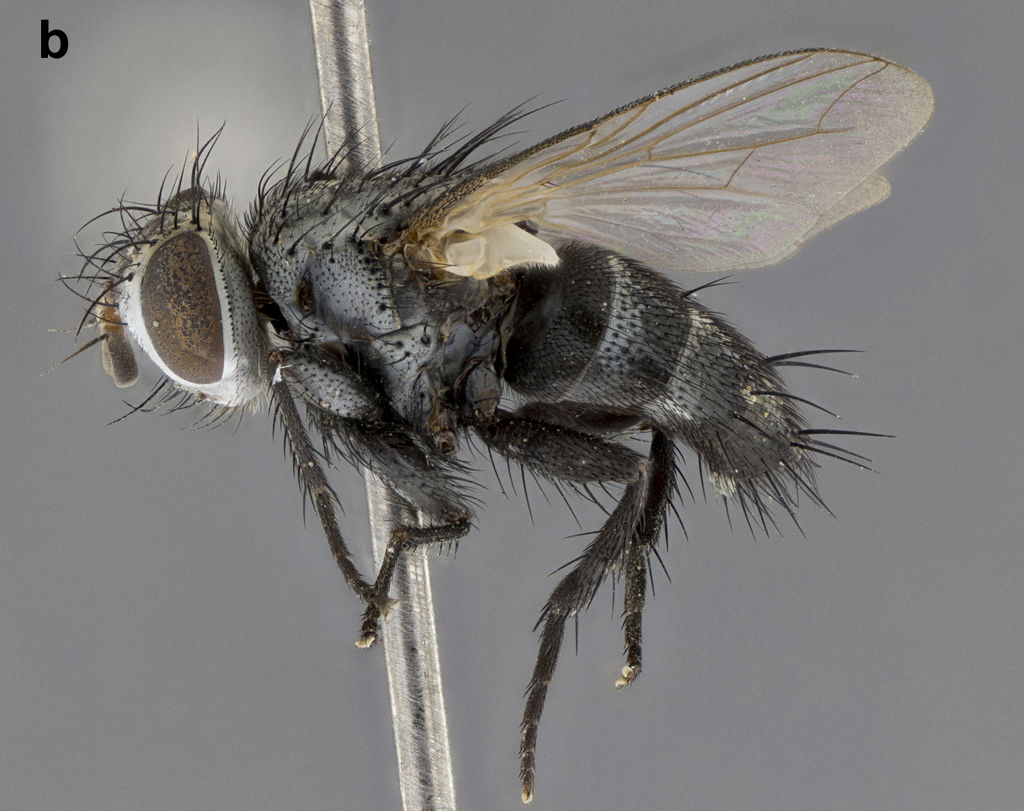
lateral view

**Figure 12c.**
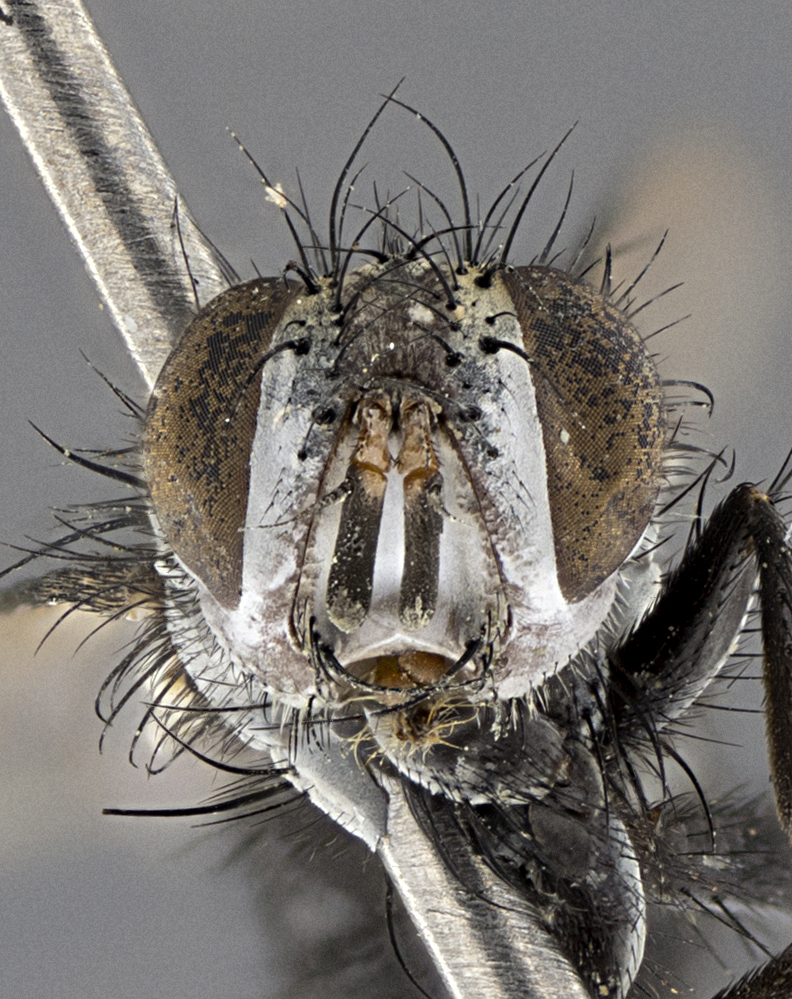
frontal view

**Figure 12d.**
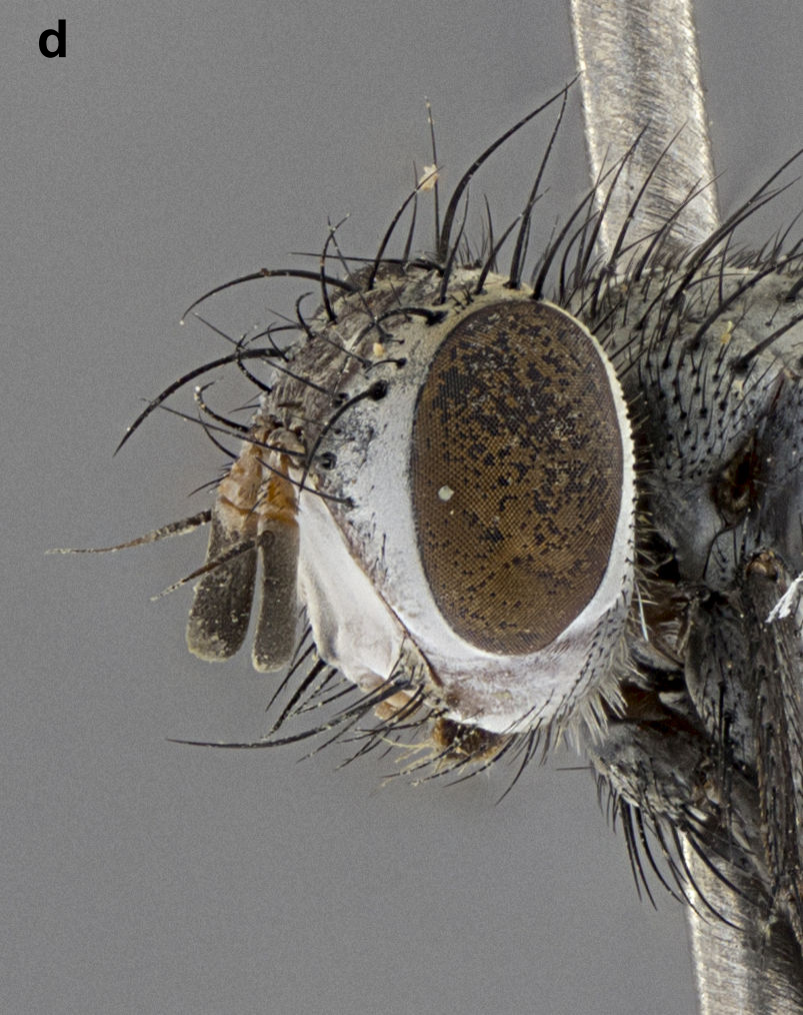
three quarters view

**Figure 13a.**
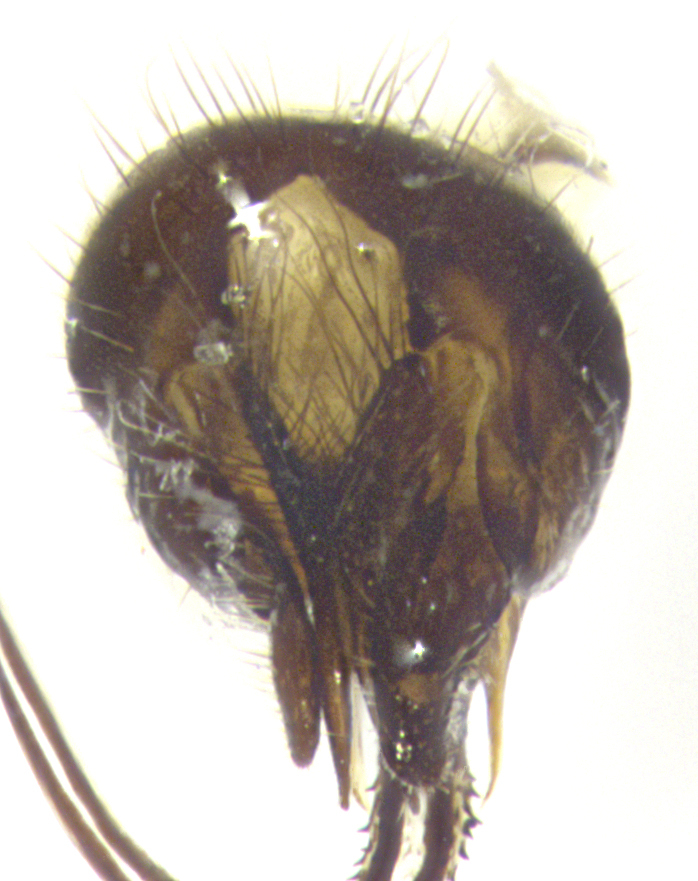
caudal view

**Figure 13b.**
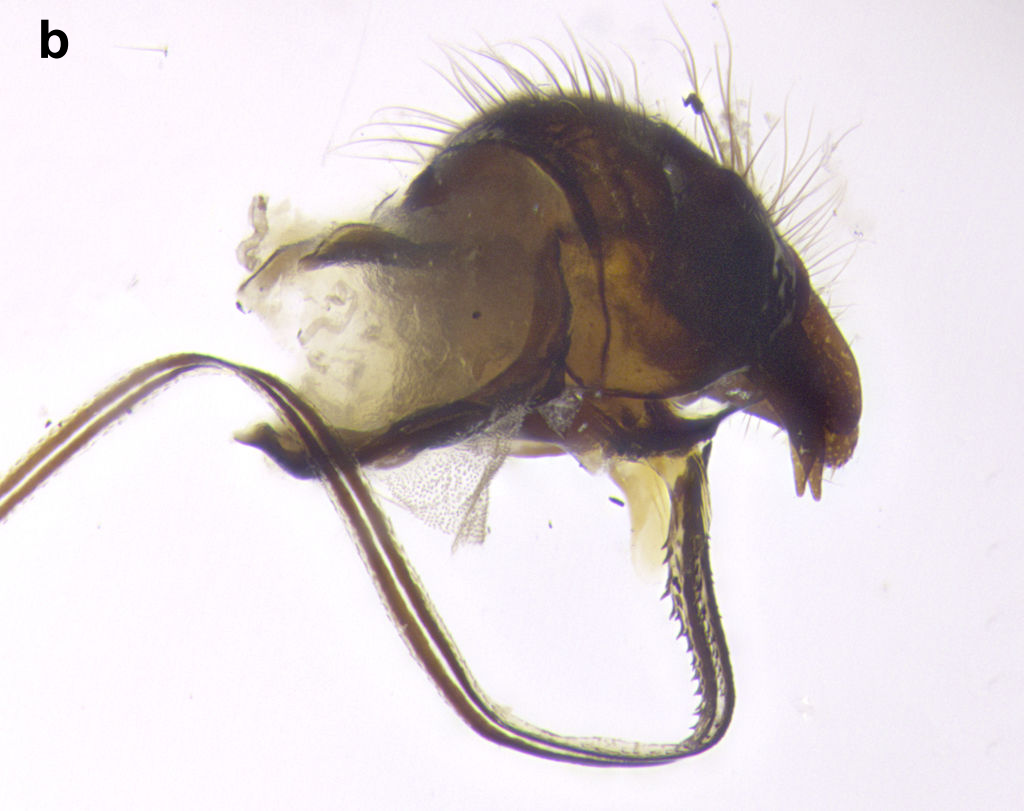
lateral view

**Figure 13c.**
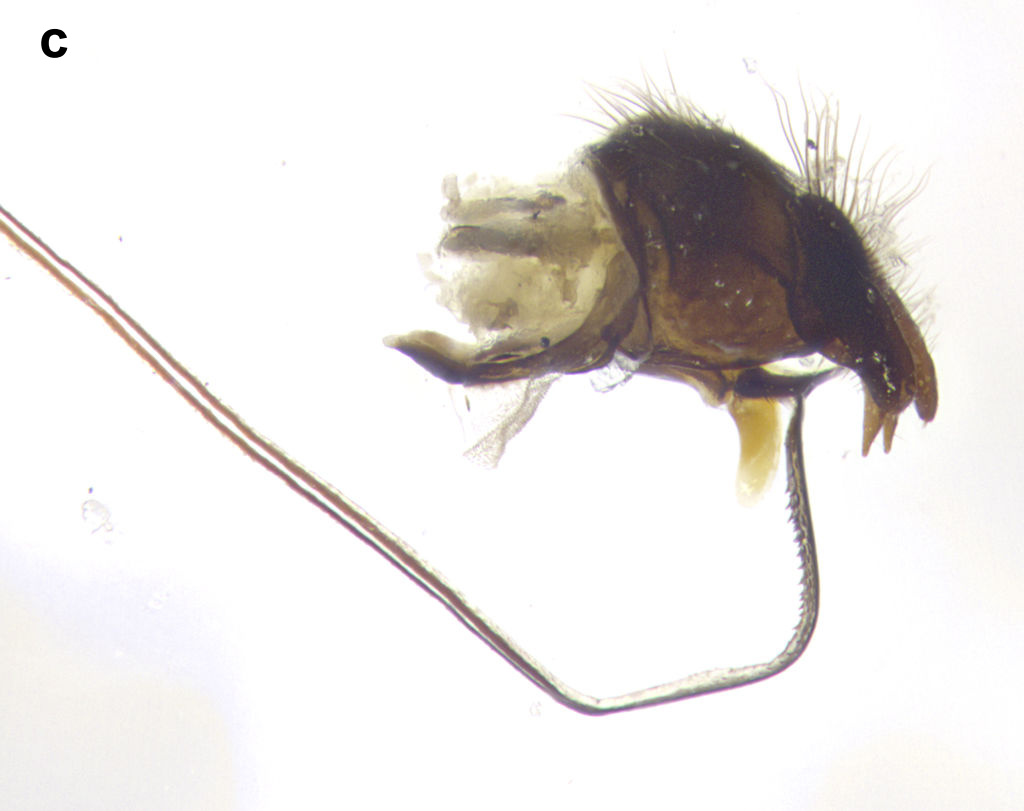
something

**Figure 13d.**
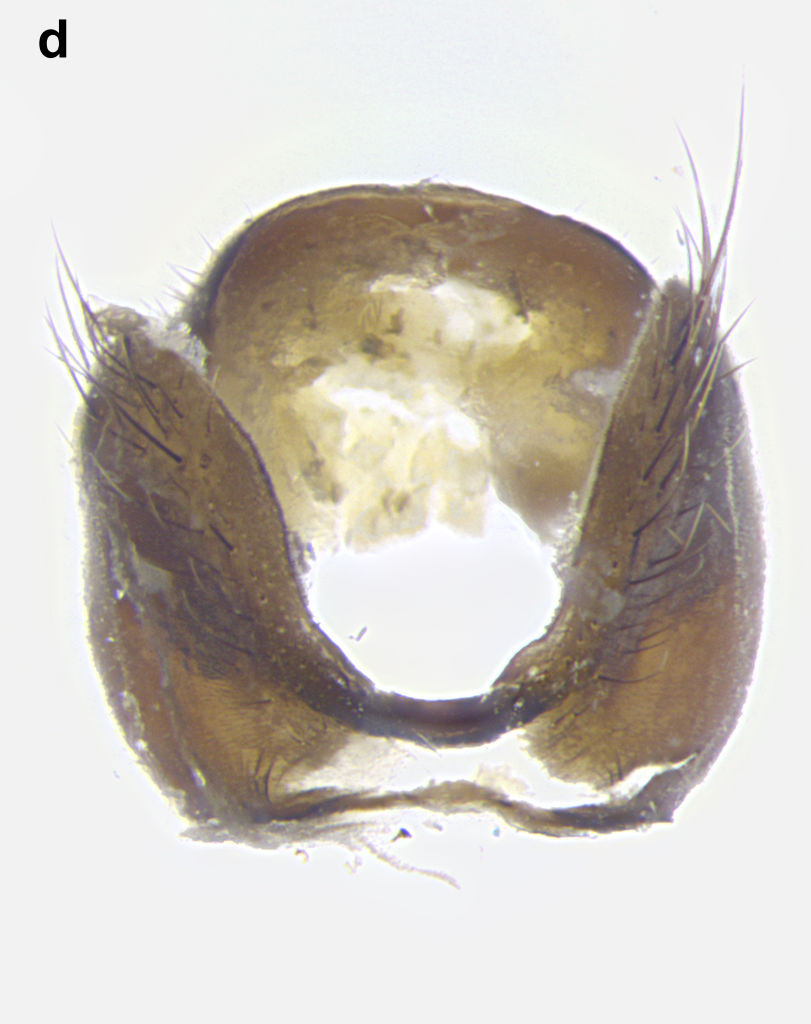
sternite 5, ventral view

**Figure 14a.**
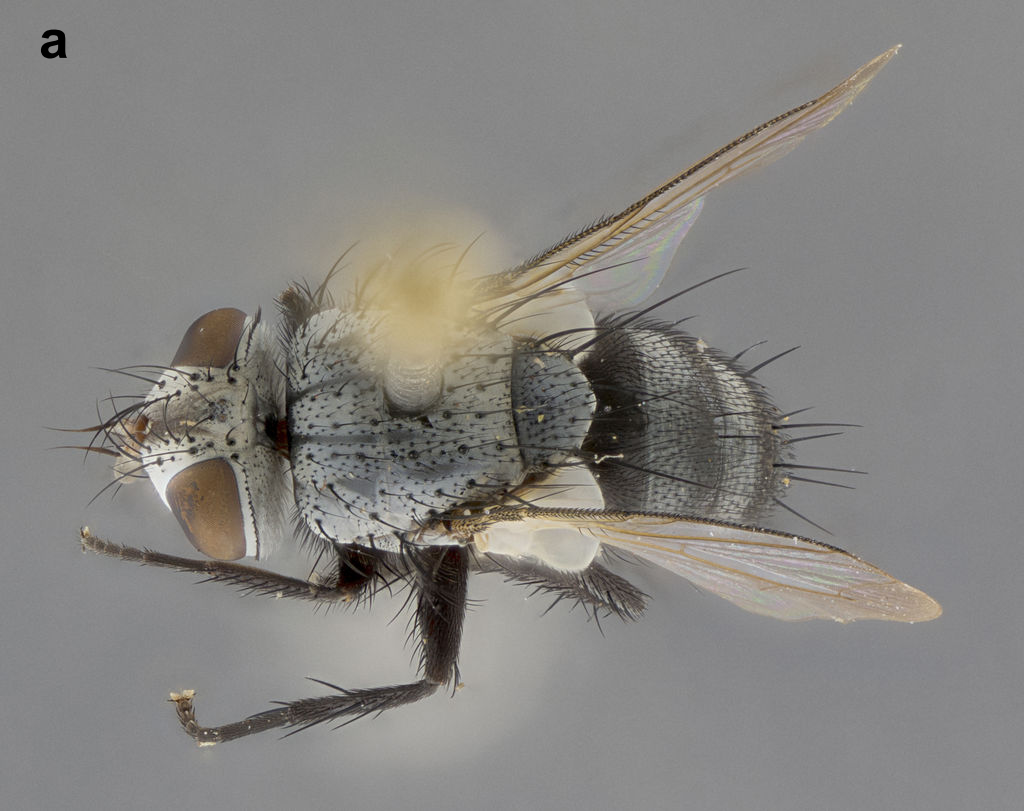
dorsal view

**Figure 14b.**
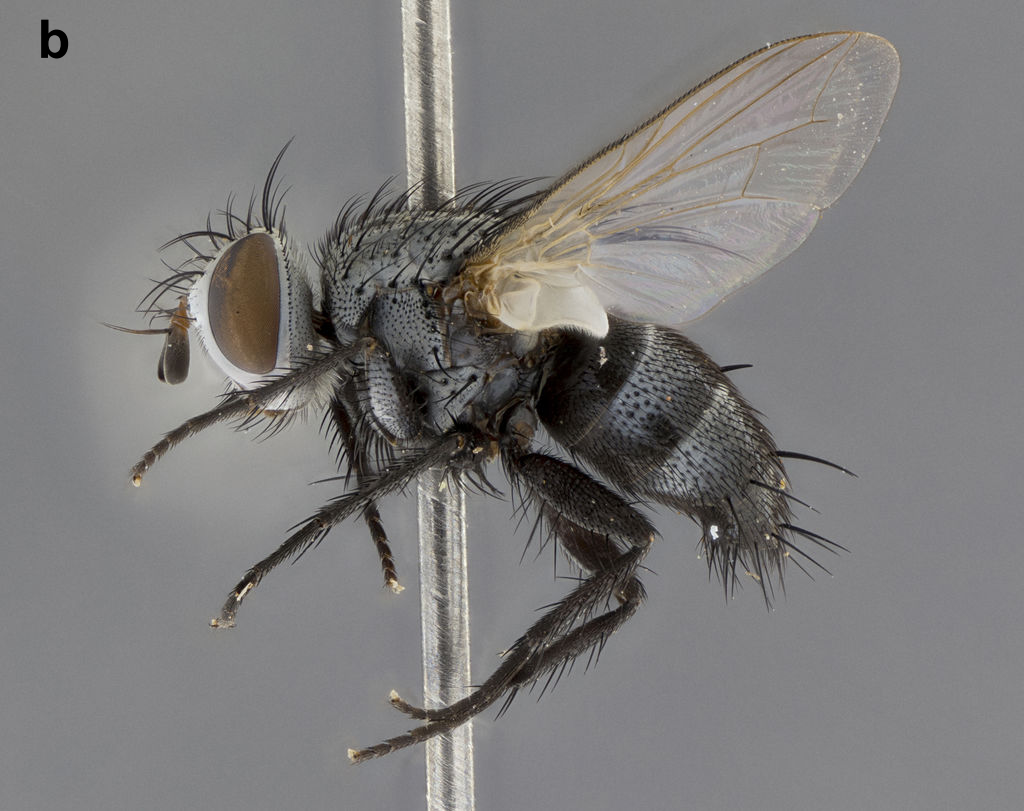
lateral view

**Figure 14c.**
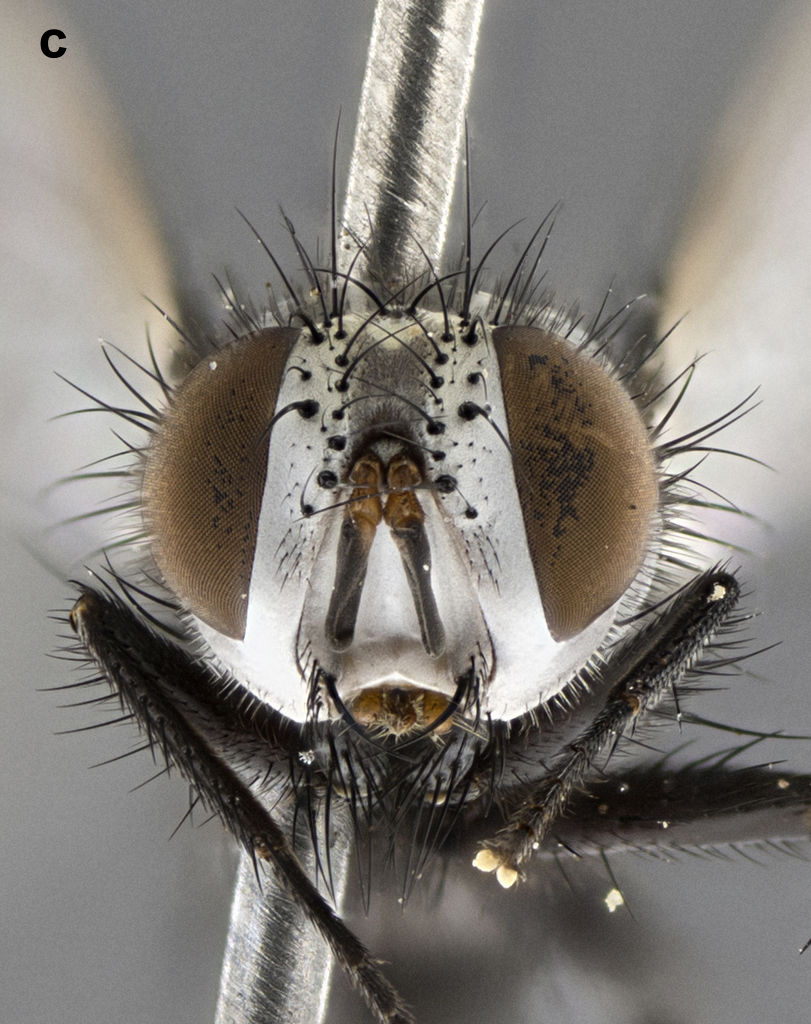
frontal view

**Figure 14d.**
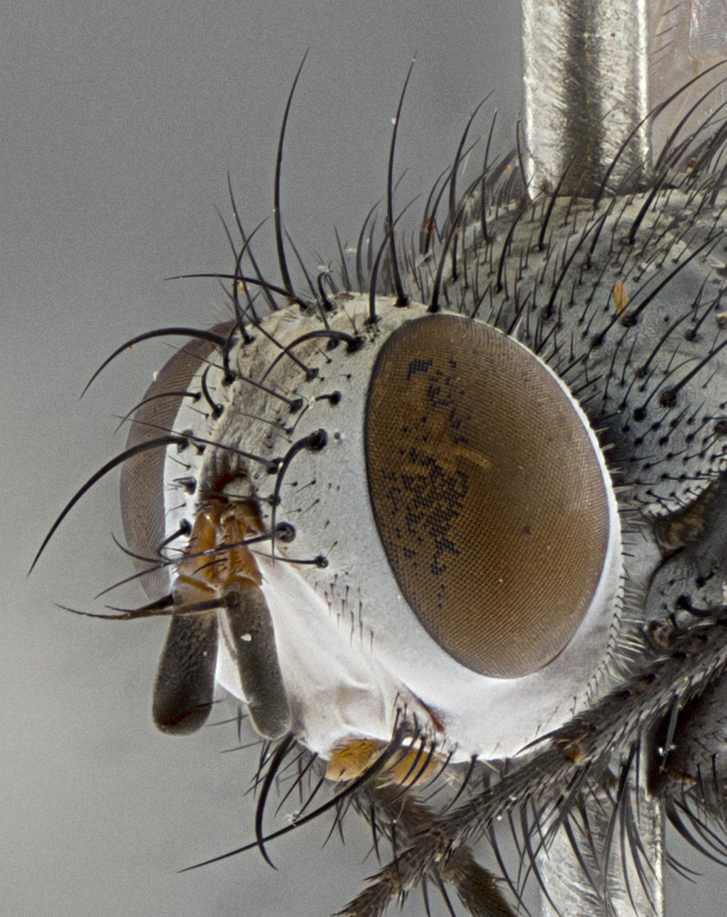
three quarters view
